# Scalable De Novo Synthesis of Aldgarose and Total Synthesis of Aldgamycin N

**DOI:** 10.1002/anie.202016477

**Published:** 2021-03-03

**Authors:** Georg Späth, Alois Fürstner

**Affiliations:** ^1^ Max-Planck-Institut für Kohlenforschung 45470 Mülheim/Ruhr Germany

**Keywords:** antibiotics, glycosidation, macrolides, monosaccharides, *trans*-hydrostannation

## Abstract

Since the accompanying study had shown that the introduction of the eponymous aldgarose sugar to the C5‐OH group of the macrocyclic aglycone of aldgamycin N is most difficult, if not even impossible, the synthesis route was revised and the glycosidation performed at an earlier stage. To mitigate the “cost” of this strategic amendment, a practical and scalable de novo synthesis of this branched octose was developed. The glycoside formation required mild conditions; it commenced with the reaction of the aglycone with the trichloroacetimidate donor to give a transient orthoester, which slowly rearranged to the desired aldgaropyranoside. The presence of the polar peripheral groups in the product did not impede the selective late‐stage functionalization of the macrolide ring itself: the contained propargylic alcohol entity was readily transformed into the characteristic acyloin motif of the target by a ruthenium‐catalyzed *trans*‐hydrostannation followed by a modified Chan‐Lam‐type coupling.

## Graphical abstract

One sugar has to be attached before the transannular ketone is revealed from an alkyne precursor to render the first successful total synthesis ever of a member of the aldgamycin family of antibiotics. Although this phasing of events implies that the precious branched monosaccharide must be carried through several steps, a practical and scalable synthesis of the required aldgarose fragment greatly mitigates the “cost” of this strategy.

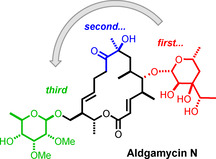

## Introduction

**Scheme 1:**
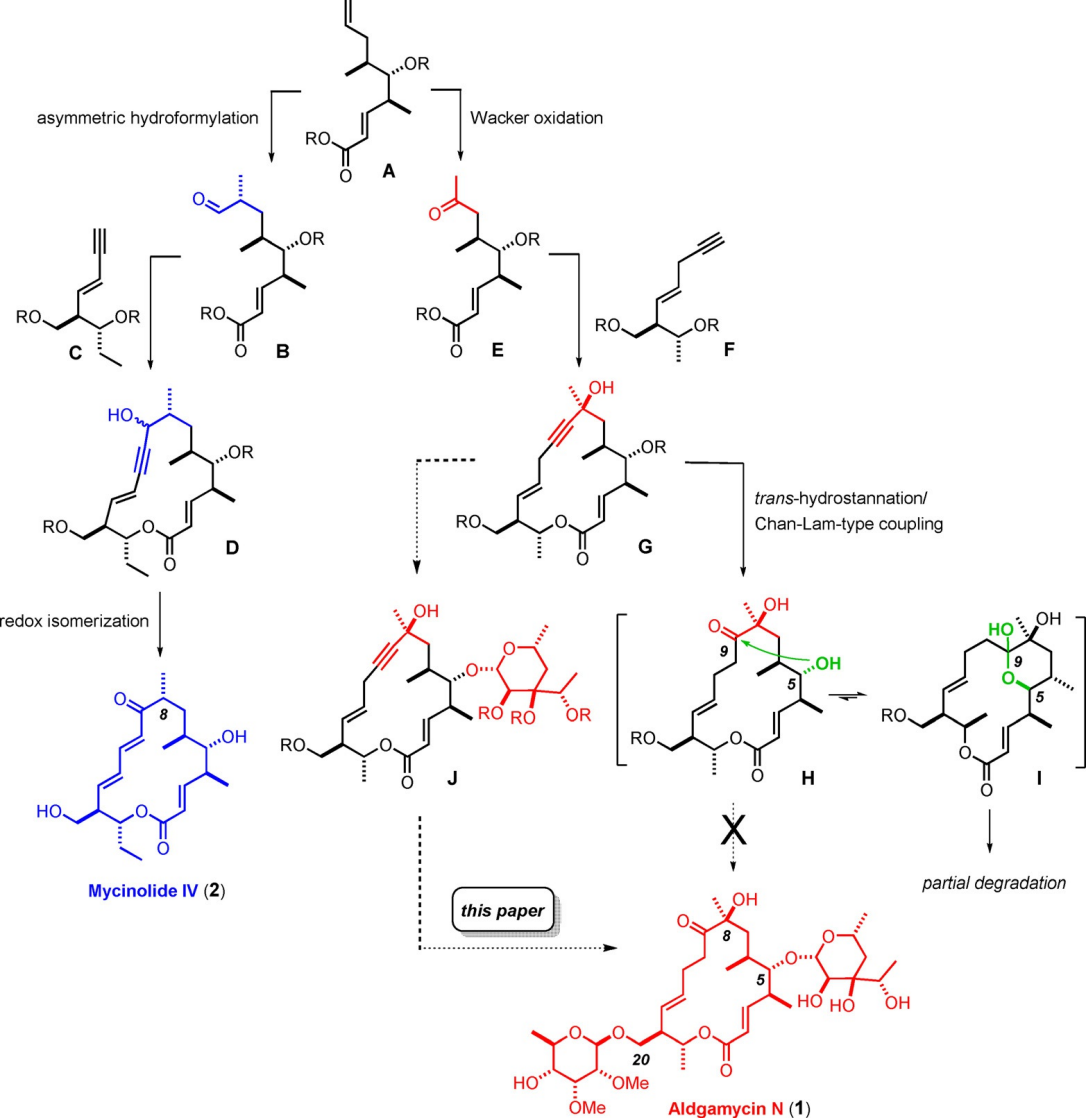
Layout of a unified approach to diverse 16‐membered macrolides: success, failure, and revision.

As outlined in the accompanying paper, we saw the opportunity to assemble a number of 16‐membered macrolide antibiotics by a unified approach that requires a single building block representing the “eastern” sector of these targets.^[1]^ Divergent functionalization of the alkene terminus of fragment **A** by either Wacker oxidation or a branch‐selective asymmetric hydroformylation opens entry into the two basic subsets of these antibiotics, which differ from each other in the oxygenation pattern at C8 (Scheme 1). Mycinolide IV (**2**) is representative for the first series distinguished by a simple methyl branch adjacent to the invariable carbonyl group at C9;^[2]^ its total synthesis is described in the accompanying paper.^[1]^ Aldgamycin N (**1**) stands for the second subset featuring a *tert*‐alcohol at this position.^[3]^ Although the viability of all key steps leading from **A** to **1** could indeed be demonstrated, the final conquest of this challenging target failed because of an unforeseen transannular cyclization engaging the C5‐OH and the ketone at C9 in lactol formation (**H**/**I**); this incident prevented the introduction of the eponymous aldgaropyranose at the proper site in the penultimate step of the synthesis prior to global deprotection.[^1^, ^4^, ^5^]

In conceptual terms, it should suffice to change the order of events to bring aldgamycin N (**1**) into reach (Scheme 1). The staging point for the introduction of the sugar, however, deserves careful consideration as it is arguably of paramount importance for the overall efficiency of the route: this unusual eight‐carbon branched monosaccharide should be carried through as few steps of the longest linear sequence as possible. In consideration thereof, the critical glycosidation was timed after closure of the macrocycle just before the carbonyl group is unveiled by formal hydration of the triple bond at the more hindered site (**G** → **J** → **1**). Even this revised plan remains “expensive” if aldgarose were to be prepared by one of the two known syntheses described in the literature.[^6^, ^7^, ^8^] As first interim goal we therefore planned to develop a practical and scalable new route to this precious branched octose.

## Results and Discussion

**Scheme 2:**
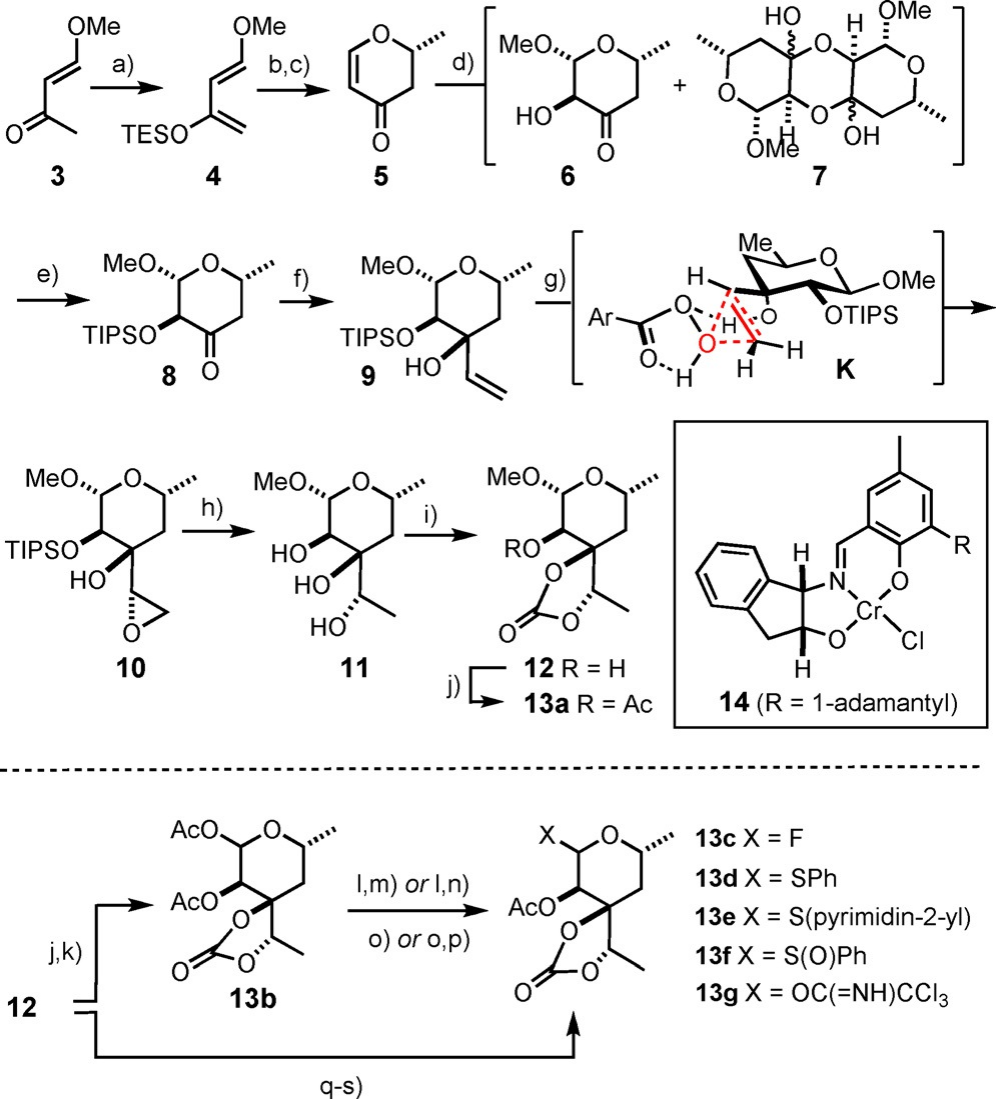
a) TESOTf, Et_3_N, Et_2_O, −20 °C, 92 %; b) **14** (1.5 mol %), MeCHO (neat), −20 °C → RT; c) TFA, CH_2_Cl_2_, 61 % (93 % *ee*); d) H_2_O_2_, MeOH, aq. NaOH, −45 °C; e) TIPSCl, imidazole, DMF; f) vinylmagnesium bromide, Et_2_O, THF, −78 °C, dr=4:1, 46 % (**9**, pure isomer, over three steps); g) *m*CPBA, CH_2_Cl_2_, 0 °C → RT, 81 % (dr=10:1); h) LiAlH_4_, Et_2_O, 0 °C → RT, 91 %; i) COCl_2_, CH_2_Cl_2_, pyridine, 0 °C, 80 %; j) Ac_2_O, Et_3_N, DMAP cat., 0 °C → RT, 81 %; k) Ac_2_O, H_2_SO_4_, 0 °C → rt, 98 %; l) BnNH_2_, THF, rt, 70 % (α:β=1:15); m) DAST, CH_2_Cl_2_, −15 °C, 78 % (**13 c**, α:β=1:2); n) Cl_3_CCN, DBU, CH_2_Cl_2_, 92 % (**13 g**, α:β=1:3); o) PhSH, SnCl_4_, CH_2_Cl_2_, 0 °C, 83 % (**13 d**, α:β≈2:3); p) *m*CPBA, CH_2_Cl_2_, 50 % (**13 f**); q) TFA/H_2_O, 100 °C, 67 % (α:β=1:8); r) PEt_3_, (pyrimdin‐2‐yl)_2_S_2_, CH_3_CN, 0 °C, 77 %; s) Ac_2_O, Et_3_N, DMAP cat., rt, 71 % (**13 e**, β‐anomer); DAST=diethyl‐ aminosulfur trifluoride; DMAP=4‐dimethylaminopyridine; *m*CPBA=*meta*‐chloroperbenzoic acid; TES=triethylsilyl; Tf=trifluoromethanesulfonyl; TFA=trifluoroacetic acid; TIPS=triisopropylsilyl.

To this end, we opted for a de novo synthesis in order to avoid any stepwise defunctionalization at the outset, which the known literature routes starting from D‐galactose pentaacetate or methyl α‐D‐glucopyranoside had to implement.[^6^, ^7^] Rather, we resorted to an asymmetric hetero‐Diels–Alder reaction between a Danishefsky diene^[9]^ and acetaldehyde catalyzed by the chiral chromium complex **14** (Scheme 2).^[10]^ For practical purposes, the more stable TES‐ether variant **4** ^[11]^ was chosen because it made the isolation of the cycloadduct from the self‐condensation products of acetaldehyde much easier on multigram scale; treatment of the crude material with trifluoroacetic acid then unveiled pyranone **5** ^[12]^ in good yield with an *ee* of 93 %. This compound was subjected to epoxidation/ring opening on exposure to H_2_O_2_ in MeOH under basic conditions to afford the 1,2‐*trans*‐configured ketol **6** and the derived dimer **7**. This product distribution was inconsequential because addition of TIPSCl and imidazole cracked the latter species and furnished compound **8** exclusively, which was immediately used in the next step. In addition to this favorable control over the product distribution, the bulky silyl group also served the addition of vinylmagnesium bromide to the carbonyl group well, in that it favored the formation of the equatorially‐branched product;^[13]^ after separation of the minor isomer, the required alcohol **9** was obtained in analytically pure form in 46 % yield over three steps on 1.8 g scale (single largest batch).

**Figure 1:**
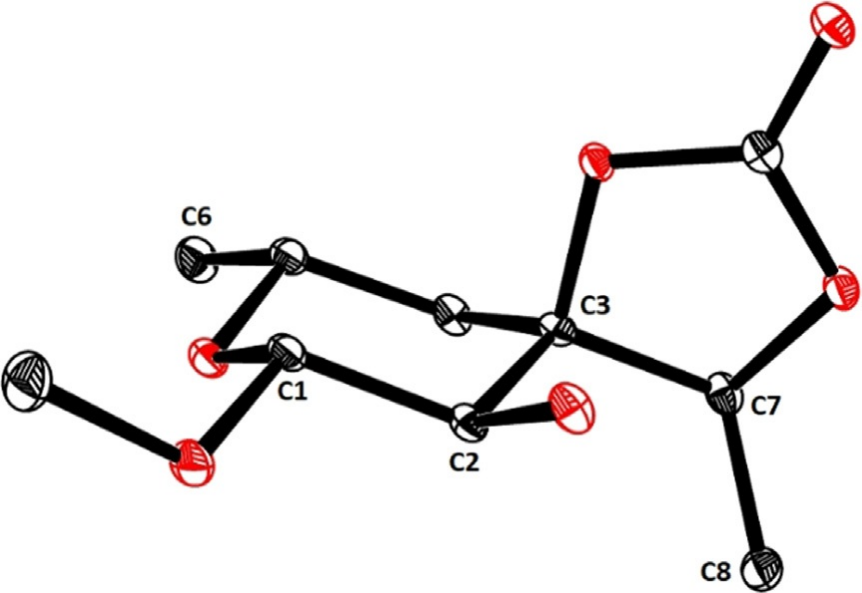
Structure of methyl β‐D‐aldgaropyranoside (**12**) in the solid state (carbohydrate numbering Scheme).^[39]^

The fact that the subsequent epoxidation of the double bond on reaction with *m*CPBA proceeded with excellent diastereoselectivity is thought to reflect a highly ordered transition state **K** in which the axial ‐OH group directs the incoming reagent to the proper π‐face via hydrogen bonding.[^14^, ^15^] With the *S*‐configured exocyclic C7 stereocenter of aldgarose set, the oxirane ring was opened on treatment with LiAlH_4_, resulting in concomitant cleavage of the adjacent TIPS‐ether.^[16]^ Therefore, the use of this silyl group, which had paid valuable dividends with regards to efficiency and selectivity, came without further cost in terms of the step count. Finally, reaction of the resulting triol **11** with phosgene in CH_2_Cl_2_/pyridine furnished methyl β‐D‐aldgaropyranoside (**12**), carrying the cyclic carbonate at the exocyclic site.^[17]^ The stereochemical and constitutional integrity of this compound was confirmed by X‐ray diffraction (Figure 1). As a necessary prelude for the upcoming glycosidation event, **12** was then acetylated to ensure anchimeric assistance before the anomeric center was transformed into a panel of glycosyl donors (**13 b**–**g**) shown in Scheme 2.

**Scheme 3:**
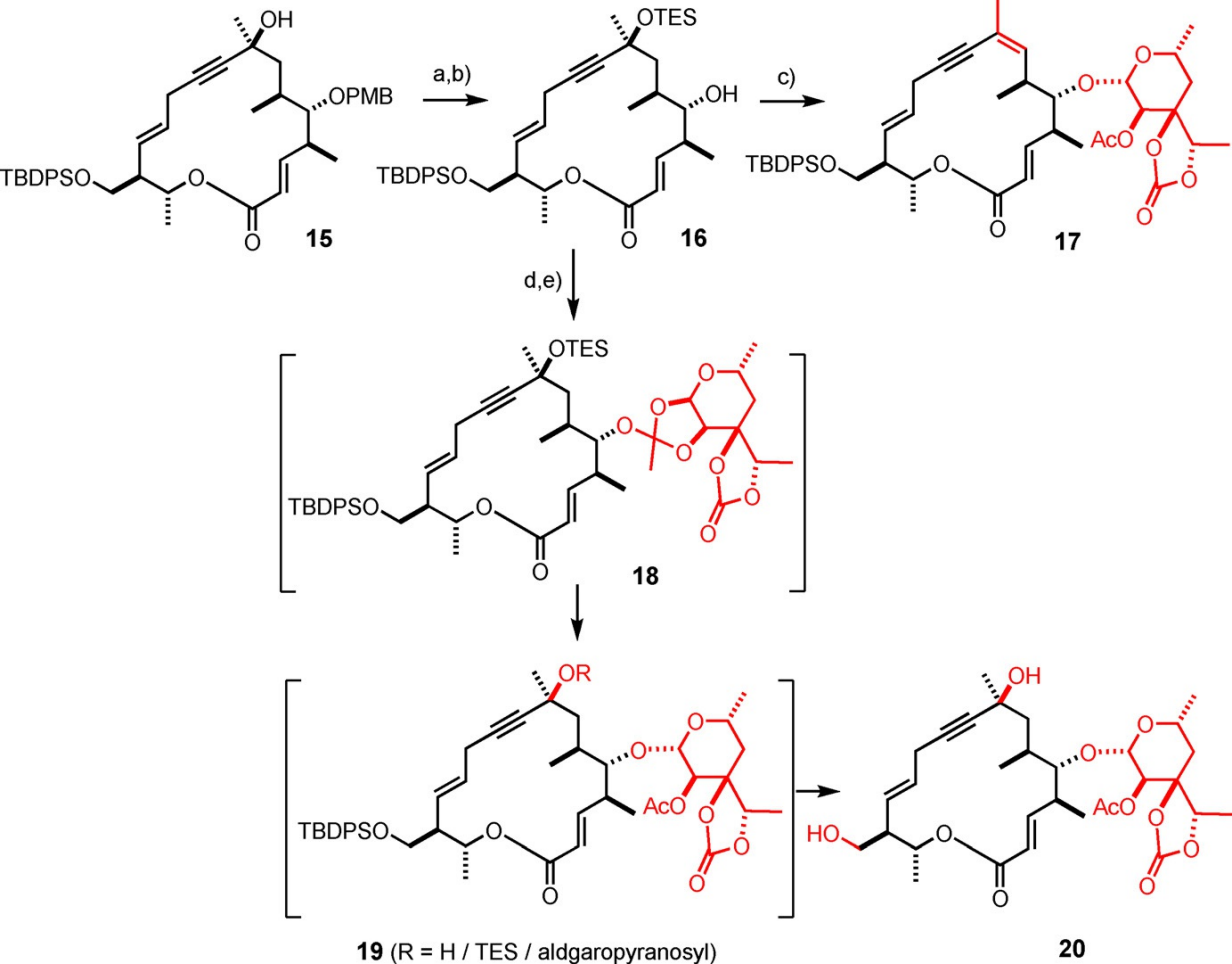
a) TESOTf, 2,6‐lutidine, CH_2_Cl_2_, −25 °C, 91 %; b) DDQ, CH_2_Cl_2_, H_2_O, 86 %; c) **13 g**, TMSOTf cat., CH_2_Cl_2_, −45 °C, 66 %; d) **13 g**, TESOTf cat., CH_2_Cl_2_, −78 °C; e) TASF, DMF, H_2_O, 0 °C → RT, 53 % (over two steps); DDQ=2,3‐dichloro‐5,6‐dicyano‐1,4‐benzoquinone; TASF=tris(dimethylamino)sulfonium difluorotrimethylsilicate.

With ample material at hand, the stage was set for the critical attachment of the aldgarose to the macrocyclic aglycone. In this context, much hope had been placed on the corresponding glycosyl fluoride **13 c** because a previous total synthesis of the related macrolide antibiotic mycinamicin IV had greatly benefitted from the use of donors of this type and the mild conditions for their activation.[^18^, ^19^] Unfortunately, however, this encouraging precedent did not substantiate in the present case; the use of various other donors (**13 b**–**f**) was to no avail either. Only the corresponding trichloroacetimidate **13 g** gave a hit, even though the final solution was also far from obvious (Scheme 3).^[20]^ First, we had to learn that the tertiary alcohol at C8 was by no means innocent: depending on the chosen conditions, it either proved unstable or sufficiently reactive to participate in glycosidation. Therefore this site was first protected as TES‐ether before the secondary ‐OH group at C5 was unveiled. When the reaction of compound **16** thus formed with **13 g** was induced with either TMSOTf or TESOTf as the promotor of choice in CH_2_Cl_2_ at −45 °C,^[21]^ the desired β‐glycosidic bond was formed exclusively but the tertiary silyl ether was eliminated to give enyne **17** as a single geometrical isomer. Upon lowering the temperature to −78 °C, the ether subsisted but the glycoside formation stalled at the orthoester stage. As expected, however, **18** slowly rearranged to the desired β‐glycoside **19** on prolonged stirring, provided that the temperature never rose above −78 °C.[^22^, ^23^, ^24^, ^25^] Even then, partial cleavage of the ‐OTES‐group could not be fully suppressed, giving the undraped tertiary alcohol once again the chance to interfere, even though this side reaction was minor. To facilitate the purification, the crude mixture was treated with TASF in aqueous DMF to take both silyl groups off;^[26]^ the resulting more polar diol could be rigorously purified to give compound **20** in analytically pure form in 53 % over both steps (120 mg scale, single largest batch). This outcome is deemed satisfactory in consideration of the very fragile nature of all intermediates and the delicacy of the maneuver.

**Scheme 4:**
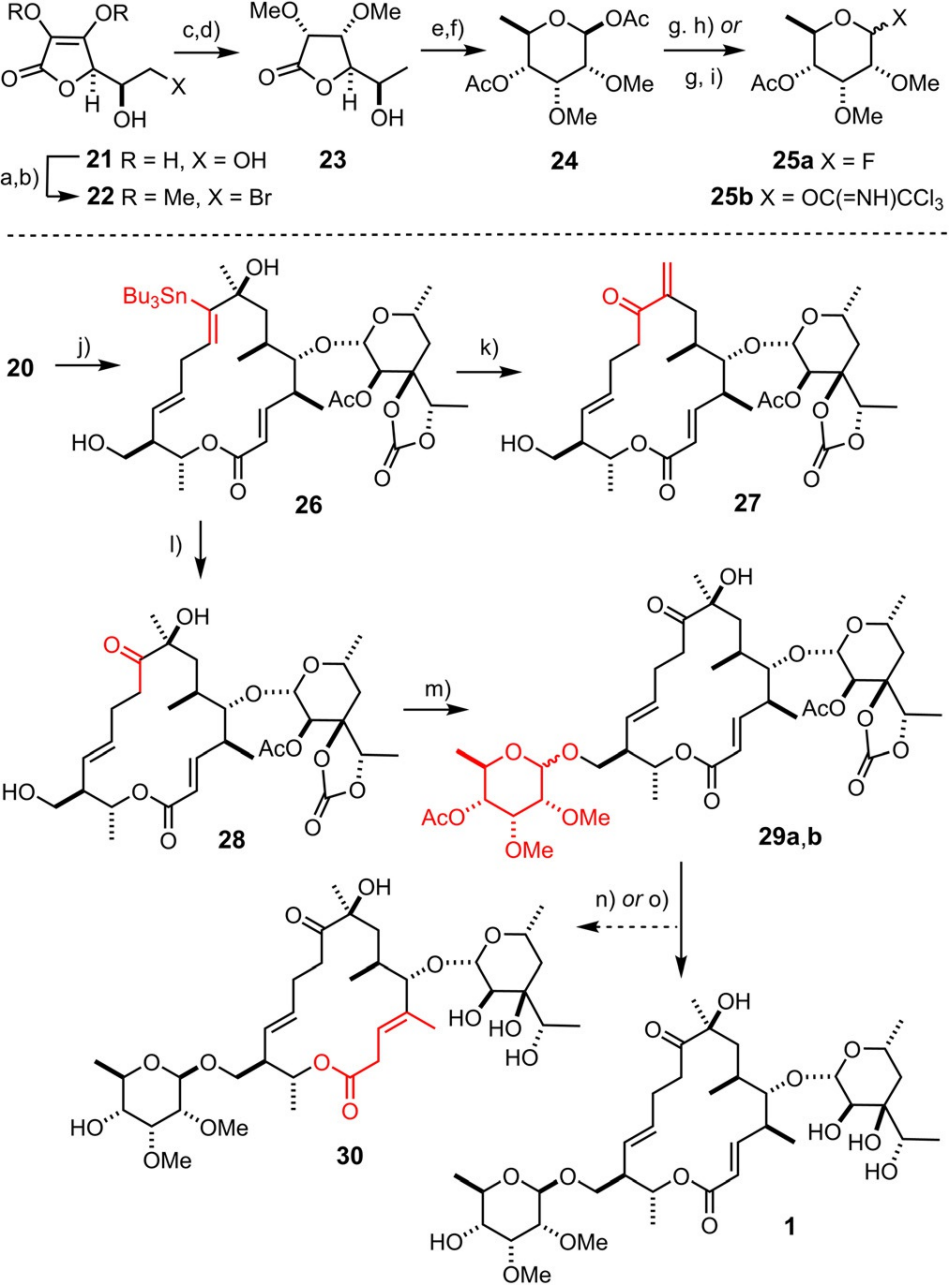
a) HBr, HOAc, then H_2_O, 84 %; b) TMSCHN_2_, toluene, MeOH, 0 °C → RT, 78 %; c) H_2_ (1 bar), Pd/C (10 % *w/w*), MeOH, Et_3_N, 89 %; d) [Rh(dppb)(cod)]BF_4_ (10 mol %), H_2_ (100 bar), CH_2_Cl_2_, 94 %; e) Dibal‐H, toluene, −78 °C → −55 °C; f) Ac_2_O, H_2_SO_4_, 0 °C → RT, 48 %; g) BnNH_2_, THF, 49 %; h) HF⋅pyridine, CH_2_Cl_2_, 0 °C, 86 % (α:β=4:1); i) Cl_3_CCN, DBU, CH_2_Cl_2_, 60 % (α:β=1:12); j) [Cp*RuCl]_4_ (10 mol %), Bu_3_SnH, CH_2_Cl_2_, 62 %; k) Cu(OAc)_2_⋅H_2_O, DMAP, DMSO, see Text; l) Cu(tfa)_2_⋅H_2_O, 2,6‐di‐*tert*‐butylpyridine, DMSO, 48 °C, 61 %; m) **25 b**, TESOTf, CH_2_Cl_2_, MeCN, −40 °C, 50 % (β‐anomer) + 27 % (α‐anomer); n) K_2_CO_3_, MeOH, 32 % (**1**) + 8 % (**30**), see main text; o) Ba(OH)_2_⋅8 H_2_O, H_2_O, THF, 69 % (**1**); cod=1,5‐cyclooctadiene; Cp*=pentamethylcyclopentadienyl; DBU=1,8‐diazabicyclo[5.4.0]undec‐7‐ene; Dibal‐H=diisobutyl‐aluminum hydride; dppb=bis(diphenylphosphino)‐butane; tfa=trifluoroacetate.

Equally gratifying was the fact that the subsequent ruthenium‐catalyzed *trans*‐hydrostannation of the triple bond remained unaffected by the dense peripheral decoration of **20** with polar substituents, furnishing product **26** as a single isomer (Scheme 4).[^27^, ^28^] Once again, the faithful delivery of the Bu_3_Sn‐ residue to the C‐atom of the triple bond proximal to the directing propargylic ‐OH group is noteworthy, as is the unorthodox *trans*‐selective course of the addition process itself that violates conventional logic.^[29]^ At this stage, the use of Cu(tfa)_2_ instead of Cu(OAc)_2_ for the subsequent Chan‐Lam‐type coupling, which has already been alluded to in the accompanying paper,[^1^, ^30^] proved instrumental: although Cu(OAc)_2_ transformed the alkenylstannane into the targeted ketone, its use inevitably leads to acylation of the adjacent hydroxy group;^[30]^ the resulting tertiary acetate, however, is unstable under the reaction conditions and succumbed to elimination as can be judged from the isolation of small amounts of the exocyclic enone **27** from one of the resulting mixtures. This fatal path is prevented with Cu(tfa)_2_ as the reagent, which furnished the desired unprotected acyloin **28** in 61 % yield^[31]^ in readiness for attachment of the yet missing mycinopyranose and completion of the total synthesis.

The second required sugar building block was obtained from D‐isoascorbic acid by following a literature procedure (Scheme 4, top).^[32]^ The only significant modification concerned the elaboration of the mixture formed upon Dibal‐H reduction of lactone **23**: whereas the literature claims a rearrangement with exclusive formation of the pyranose form in acidic medium, this transformation did not work in our hands but invariably gave rather complex product distributions, despite considerable experimentation. However, acylation of the crude material proved viable and provided the corresponding acetate **24**, although in a more modest yield of 48 %. This product was then elaborated into the corresponding glycosyl fluoride **25 a** as well as the trichloroacet‐ imidate **25 b**. The exact same anomeric fluoride had previously been used with great success in a total synthesis of mycinamicin IV for the glycosidation of the analogous position of the molecule; this step had worked in good yield and was distinguished by a truly outstanding β‐selectivity.[^18^, ^19^] Unfortunately, neither of these virtues could be harnessed when the same conditions were applied in the present case: rather, a mixture was generated that comprised both product anomers in a 1:1 ratio, as well as substantial amounts of double‐glycosylated product and unreacted starting material. Once again the trichloroacetimidate proved superior in that no competing glycosylation or elimination of the tertiary alcohol was noticed. Even though acetonitrile was used as a cosolvent to bolster the stereochemical course of the reaction,[^33^, ^34^] a modest α:β ratio of ≈1:2 was obtained; such a result is to be expected in a case like this, since the adjacent position on the mycinose donor is a methyl ether that cannot exert any anchimeric assistance.^[35]^ The anomers **29 a**,**b** were readily separable; the final deprotection was best performed with Ba(OH)_2_ in aqueous THF, whereas the use of K_2_CO_3_ in MeOH furnished substantial amounts of the positional isomer **30** (despite incomplete conversion), in which the double bond of the former enoate got deconjugated from the lactone carbonyl. The analytical and spectral data of aldgamycin N (**1**) thus formed not only matched those of the isolated material very well, but the recorded 1D ^13^C NMR spectra even show those signals that are hidden in the baseline of the original spectra due to massive line broadening.^[3]^

## Conclusion

We hence conclude that this second foray into the aldgamycin family of antibiotics based on an “early” glycosidation event proved successful. When taken together with the total synthesis of mycinolide IV described in the accompanying paper,^[1]^ the overall project goes beyond the conquest of two individual targets;[^36^, ^37^] rather, it provides the tantalizing outlook that a larger ensemble of bioactive macrolides of this challenging molecular estate can be made from a rather small number of building blocks.^[38]^ Most notably, a single fragment sufficed to cover both basic formats of their eastern sectors; the different levels of unsaturation featured in the western parts of this target class can be encoded as readily accessible alkynes. Permutation of these modules and the basic operations for their assembly, in concord with proper glycosidation events, should bring a considerable number of natural and non‐natural antibiotics of this type into reach for biological and pharmacological evaluation. We are committed to explore this possibility in more detail and will report our results in due course.

## Conflict of interest

The authors declare no conflict of interest.

## Supporting information

As a service to our authors and readers, this journal provides supporting information supplied by the authors. Such materials are peer reviewed and may be re‐organized for online delivery, but are not copy‐edited or typeset. Technical support issues arising from supporting information (other than missing files) should be addressed to the authors.

Supplementary
