# Supplementary material for: Scalable De Novo Synthesis of Aldgarose and Total Synthesis of Aldgamycin N
Source: Angew Chem Int Ed Engl. 2021 Mar 3;60(14):7900–5. doi: 10.1002/anie.202016477 (PMC8048874; doi:10.1002/anie.202016477)
Supplement: Supplementary file 1 — Supplementary [file ANIE-60-7900-s001.pdf]

Angewandte Chemie
Eine Zeitschrift der Gesellschaft Deutscher Chemiker

Supporting Information

Scalable De Novo Synthesis of Aldgarose and Total Synthesis of Aldgamycin N

*Georg Späth and Alois Fürstner**

anie_202016477_sm_miscellaneous_information.pdf

Supporting Crystallographic Information

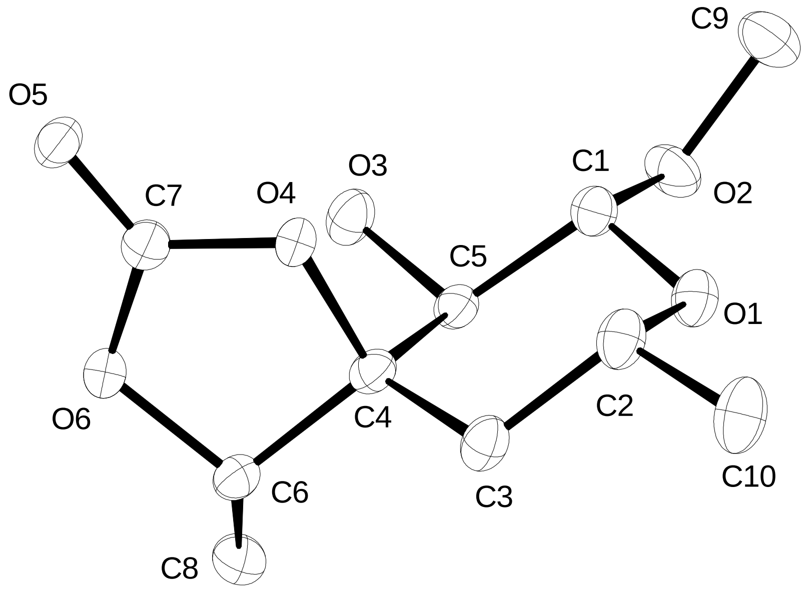

Figure S1. Structure of methyl β-D-aldgaropyranoside (**12**) in the solid state; arbitrary numbering scheme

X-ray Crystal Structure Analysis of Compound 12: $C_{10}H_{16}O_6$, M_r = 232.23 g · mol^{-1}, colourless plate, crystal size 0.781 x 0.236 x 0.228 mm^3, monoclinic, space group *P2₁* [4], a = 5.9006(2) Å, b = 10.7626(4) Å, c = 8.9193(3) Å, β = 93.5490(10)°, V = 565.34(3) Å^3, T = 100(2) K, Z = 2, D_{calc} = 1.364 g · cm^3, λ = 1.54178 Å, $\mu(Cu\text{-}K_\alpha)$ = 0.967 mm^{-1}, analytical absorption correction ($T_{\min}$ = 0.68, $T_{\max}$ = 0.85), Bruker AXS Enraf-Nonius KappaCCD diffractometer with a FR591 rotating Cu-anode X-ray source, 7.521 < θ < 72.359°, 19774 measured reflections, 2155 independent reflections, 2129 reflections with $I > 2\sigma(I)$, R_{int} = 0.0281. S = 1.194, 168 parameters, absolute structure parameter = -0.02(7), residual electron density +0.2 (0.25 Å from H3B) / -0.2 (0.81 Å from C10) e · Å^{-3}. The hydrogens at C1, C2, C4, C5 and C6 were found and refined, all other hydrogens were placed in calculated positions.

The structure was solved by *SHELXT* and refined by full-matrix least-squares (*SHELXL*) against F^2 to R_1 = 0.032 [$I > 2\sigma(I)$], wR_2 = 0.076. **CCDC-2047121**.

General.

Unless otherwise stated, all reactions were performed in oven-dried (80 °C) or flame-dried glassware in anhydrous solvents under argon, applying standard Schlenk techniques. Dry argon (>99.5%) was purchased from Air Liquide.

The following solvents were purified by distillation over the indicated drying agents and transferred under argon: tetrahydrofuran and diethyl ether (Mg/anthracene), dichloromethane (CaH_2), hexanes and toluene (Na/K), methanol (Mg, stored over 3 Å molecular sieves). Acetonitrile, dimethyl sulfoxide, dimethylformamide, pyridine and triethylamine were dried using an adsorption (molecular sieves) solvent purification system. During work up, solvents were removed under reduced pressure below 40 °C using a rotary evaporator.

Thin layer chromatography (TLC) was performed on Macherey-Nagel precoated plates (POLYGRAM® SIL/UV254); the compounds were detected by UV light (254 nm) or heating of the plate with a heat gun after treatment with stain solutions comprising either potassium permanganate or phosphomolybdic acid. Flash chromatography was performed with VWR silica gel 60 (40 – 63 µm). Automated column chromatography was conducted on a Biotage® Isolera™ or a Biotage® Selekt instrument, using the chromatography cartridges indicated in the respective procedure. Diastereomeric ratios (d.r.) of intermediates were determined by ^{1}H NMR spectroscopy from the relative integrals of sufficiently separated, characteristic signals of the respective compound.

NMR spectra were recorded on Bruker AV 400, AV 500 or AVIII 600 spectrometers in the solvents indicated. The solvent signals were used as references, chemical shifts were converted to the TMS scale and reported as follows: chemical shift in ppm (multiplicity, coupling constant *J* in Hz, number of protons). Multiplets are designated by the following abbreviations: s for singlet, d for doublet, t for triplet, q for quartet, quint for quintet, m for complex pattern (multiplet); the abbreviation br indicates a broad signal. ^{13}C NMR spectra were recorded in {^{1}H}-decoupled mode. Melting points were determined using a Büchi B-540 apparatus. IR spectra were recorded on a Bruker Alpha Platinum ATR spectrometer at room temperature. Mass spectra were recorded using the following instruments: MS (EI): Finnigan MAT 8200 (70 eV), ESI-MS: Bruker ESQ3000, accurate mass determinations: Bruker APEX III FT-MS (7 T magnet) or Finnigan MAT 95. GC-MS samples were processed on a Shimadzu GCMS-QP2010 Ultrainstrument. Specific optical rotatory power ($[\alpha]_D$) was measured with an A-Krüss Otronic Model P8000-t polarimeter at a wavelength of 589 nm. The values are given with respect to exact temperature, concentration (c/(10mg/mL)) and solvent.

De Novo Synthesis of Methyl D-Aldgaroside and the Derived Glycosyl Donor

(*E*)-Triethyl((4-methoxybuta-1,3-dien-2-yl)oxy)silane (4). In a 250-mL two-necked flask, triethylsilyl trifluoromethanesulfonate (13 mL, 57 mmol) was added over 12 min to a solution of (*E*)-4-methoxybut-3-en-2-one (**3**) (5.2 mL, 51 mmol) and triethylamine (17 mL, 100 mmol) in diethyl ether (90 mL) at −20 °C. After complete addition, the mixture was stirred for additional 10 min at −20 °C and for another 2 h at 0 °C. The mixture was diluted with hexanes (80 mL) and quickly washed with ice-cold saturated aqueous solutions of sodium bicarbonate and sodium chloride (50 mL each). The organic layer was dried over anhydrous sodium sulfate, the drying agent was filtered off, and volatile materials were removed under reduced pressure (38 °C, 20 mbar). The dark brown, oily liquid was purified by bulb-to-bulb distillation under reduced pressure (75 – 80 °C bath temperature, 10^{-3} mbar, receiving flask cooled in dry ice/acetone bath) to give the title compound as a colorless liquid (10.7 g, 97% yield). **^{1}H NMR (400 MHz, $CDCl_3$):** δ 6.89 (d, *J* = 12.3 Hz, 1H), 5.35 (d, *J* = 12.3 Hz, 1H), 4.09 – 4.04 (m, 2H), 3.59 (s, 3H), 1.03 – 0.97 (m, 9H), 0.76 – 0.68 (m, 6H). **^{13}C{^{1}H} NMR (101 MHz, $CDCl_3$):** δ 154.3, 150.4, 103.3, 90.6, 56.6, 6.89, 5.12. The analytical data are in agreement with those reported in the literature.[1]

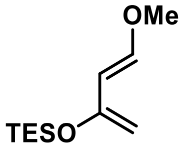

(*R*)-2-Methyl-2,3-dihydro-4*H*-pyran-4-one (5). A 25-mL Young tube was charged with the chromium complex **14** (260 mg, 0.534 mmol)[2] and powdered 4 Å molecular sieves (3.7 g). The tube was immersed into a cooling bath (–20°C) before acetaldehyde (10 mL, 178 mmol) was added (to facilitate the transfer, the flask containing the acetaldehyde was briefly cooled with dry ice). Siloxy diene **4** (7.73 g, 36.1 mmol) was then added in one portion at −20 °C. The resulting mixture was vigorously stirred in the sealed tube for 15 h while allowing the cooling bath to gradually warm to ambient temperature. The mixture was directly loaded onto a solvent-packed silica column (hexanes/EtOAc, 25:1 + 0.5 vol-% of triethylamine), which was eluted with the same solvent mixture to give the primary hetero-Diels-Alder adduct as an amber liquid (7.04 g).

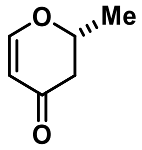

A solution of trifluoroacetic acid in dichloromethane (10% *v/v*, 3.3 mL) was added to a solution of this material in dichloromethane (35 mL) at 0°C. The cooling bath was removed and the mixture was stirred for 3 h at room temperature. The acid was quenched with triethylamine (0.65 mL) and the solvent was removed under reduced pressure. Purification of the residue by flash chromatography (pentane/*tert*-butyl methyl ether, 2:1 → 1:1) furnished the title compound as a volatile, light amber liquid (2.45 g, 61% yield, 93% *ee*). **bp** = 66-68 °C (13 mbar). **$[\alpha]_D^{20}$** = +187.2 (*c* 1.5, $CHCl_3$). **^{1}H NMR (400 MHz, $CDCl_3$):** δ 7.34 (dd, *J* = 6.0, 0.7 Hz, 1H), 5.40 (dd, *J* = 6.0, 1.1 Hz, 1H), 4.55 (dqdd, *J* = 12.7, 6.4, 4.5, 0.7 Hz, 1H), 2.51 (dd, *J* = 16.8, 12.6 Hz, 1H), 2.44 (ddd, *J* = 16.8, 4.5, 1.1 Hz, 1H), 1.46 (d, *J* = 6.3 Hz, 3H). **^{13}C{^{1}H} NMR (101 MHz, $CDCl_3$):** δ 192.8, 163.4, 107.0, 76.1, 43.6, 20.5. The enantiomeric purity was determined by GC (see below).

The racemic sample was obtained following a literature procedure.[3] The analytical data are in agreement with those reported in the literature.[4]

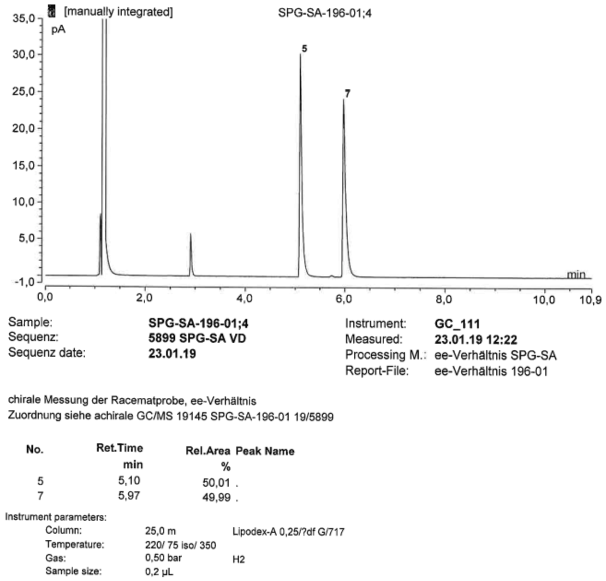

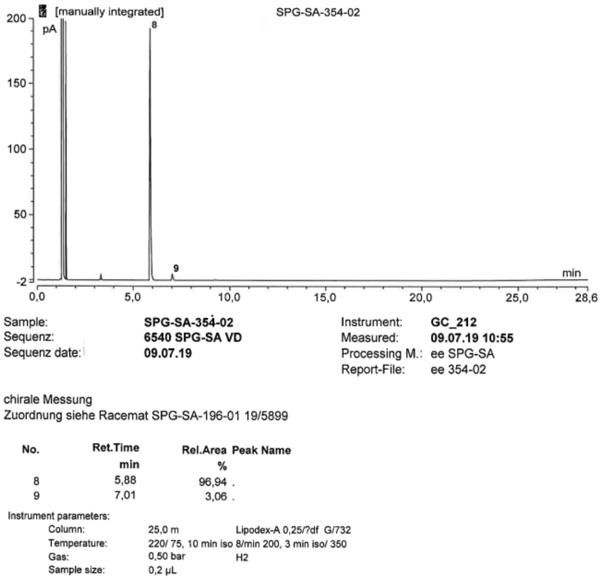

(2*R*,3*R*,4*S*,6*R*)-2-Methoxy-6-methyl-3-((triisopropylsilyl)oxy)-4-vinyltetrahydro-2*H*-pyran-4-ol (9).

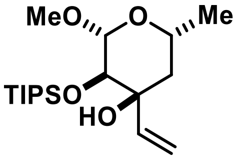

In a 100-mL two-necked flask, aqueous hydrogen peroxide solution (35% *w/w*, 2.5 mL) and aqueous sodium hydroxide solution (2 M, 0.55 mL) were sequentially added to a solution of compound **5** (1.24 g, 11.1 mmol) in methanol (52 mL) at −45 °C. Stirring was continued at that temperature for 2 h before the mixture was neutralized with acetic acid (0.07 mL) at −45 °C. Trimethyl phosphite (4.0 mL) was carefully added at this temperature and the mixture was allowed to warm to −20 °C over the course of 30 min. At this point, the same amount of trimethyl phosphite was added again. After stirring for another 30 min at −20°C, a peroxide test (Merck test strip) was negative. The mixture was warmed to ambient temperature and volatile components were removed under reduced pressure (10^{-2} mbar, 30 °C). The residue was dissolved in ethyl acetate (40 mL) and the resulting solution was dried over anhydrous sodium sulfate. The drying agent was filtered off and the solvent was removed under reduced pressure. Purification of the residue by flash chromatography (hexanes/EtOAc, 2:1) furnished a colorless gum (1.31 g), which was used in the next step without further characterization.

In a round-bottom flask, triisopropylsilyl chloride (3.4 mL, 16 mmol) and imidazole (1.28 g, 18.8 mmol) were added to a solution of this material in DMF (7.0 mL) and the resulting mixture was stirred for 15 h at room temperature. The mixture was diluted with *tert*-butyl methyl ether (40 mL) and washed with saturated aqueous sodium bicarbonate solution (40 mL). The aqueous phase was extracted with *tert*-butyl methyl

ether (4 × 15 mL) and the combined organic layers were washed with aqueous HCl (1 M, 30 mL), saturated aqueous sodium bicarbonate solution (30 mL) and brine (30 mL). The combined organic layers were dried over anhydrous sodium sulfate, the drying agent was filtered off, and the solvent was removed under reduced pressure to give crude **8** as a colorless oil (3.83 g).

Vinylmagnesium bromide (1.0 M in THF, 40 mL, 40 mmol) was added to a solution of this material in diethyl ether (45 mL) at −78 °C. After stirring for 7 h at this temperature, reaction monitoring (^{1}H NMR) indicated full consumption of the starting material. The reaction was quenched with half-saturated aqueous ammonium chloride solution (75 mL), the mixture was warmed to room temperature with vigorous stirring until a clear, biphasic mixture was obtained. The aqueous phase was extracted with *tert*-butyl methyl ether (3 × 25 mL) and the combined organic layers were dried over anhydrous sodium sulfate. The drying agent was filtered off and the solvent was removed under reduced pressure. The residue was purified by flash chromatography (hexanes/EtOAc, 90:1 → 70:1 → 60:1 → 50:1) to give the title compound as a colorless oil (1.77 g, 46% yield). $[\alpha]_D^{20}$ **=** −42.9 (*c* 1.7, $CHCl_3$). **^{1}H NMR (400 MHz, $CDCl_3$):** δ 5.82 (dd, *J* = 17.1, 10.6 Hz, 1H), 5.35 (dd, *J* = 17.1, 1.4 Hz, 1H), 5.12 (dd, *J* = 10.6, 1.3 Hz, 1H), 4.38 (d, *J* = 7.5 Hz, 1H), 3.96 (dddd, *J* = 12.5, 11.0, 6.3, 2.0 Hz, 1H), 3.50 (d, *J* = 7.5 Hz, 1H), 3.46 (s, 3H), 2.84 (d, *J* = 2.6 Hz, 1H), 1.70 (dd, *J* = 14.2, 2.1 Hz, 1H), 1.49 (ddd, *J* = 14.0, 11.1, 2.7 Hz, 1H), 1.21 (d, *J* = 6.3 Hz, 3H), 1.17 – 1.01 (m, 21H). **^{13}C{^{1}H} NMR (101 MHz, $CDCl_3$):** δ 143.5, 114.1, 102.9, 75.6, 74.7, 66.7, 56.3, 43.5, 20.7, 18.4, 13.0. **IR (film):** 3551, 2944, 2894, 2867, 1465, 1383, 1347, 1326, 1310, 1293, 1246, 1214, 1164, 1114, 1095, 1069, 1052, 1015, 923, 883, 836, 811, 681, 600, 564 cm^{-1}. **HRMS-ESI *m/z*:** $[M+Na]^+$ calcd for $C_{18}H_{36}O_4SiNa$ 367.2275; found 367.2274.

(2*R*,3*R*,4*S*,6*R*)-2-Methoxy-6-methyl-3-((triisopropylsilyl)oxy)-4-vinyltetrahydro-2*H*-pyran-4-ol (10). 3-Chloro-perbenzoic acid (77% *w/w*, 2.47 g, 11.0 mmol) was added in one portion to a solution

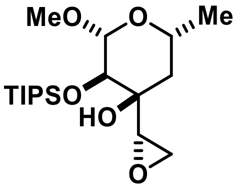

of allylic alcohol **9** (1.52 g, 4.41 mmol) in dichloromethane (40 mL) at 0°C. After stirring for 20 min at this temperature, the ice bath was removed and stirring was continued for 94 h. For work up, the flask was again immersed in an ice bath before aqueous sodium sulfite solution (10 w-%, 20 mL) was carefully added and the mixture was stirred for another 15 min. Half-saturated aqueous sodium carbonate solution (50 mL) was introduced and the mixture was warmed to room temperature under vigorous stirring. It was diluted with dichloromethane (20 mL), the layers were separated, and the organic layer was washed with half-saturated aqueous sodium carbonate solution (50 mL) and brine (20 mL). The aqueous phases were extracted with *tert*-butyl methyl ether (5 × 20 mL) and the combined organic layers dried over anhydrous sodium sulfate. The drying agent was filtered off and the solvent was removed under reduced pressure. Purification of the crude material by flash chromatography (hexanes/EtOAc, 20:1 → 10:1) furnished the title compound as a

colorless gum (1.28 g, 81% yield). $[\alpha]_D^{20}$ = −42.7 (*c* 1.2, $CHCl_3$). **^{1}H NMR (400 MHz, $CDCl_3$):** δ 4.36 (d, *J* = 7.5 Hz, 1H), 3.92 (dqd, *J* = 12.6, 6.3, 2.1 Hz, 1H), 3.65 (d, *J* = 7.6 Hz, 1H), 3.46 (s, 3H), 2.98 (dd, *J* = 3.9 Hz, 2.7 Hz, 1H), 2.81 (dd, *J* = 4.9, 2.7 Hz, 1H), 2.79 (br d, *J* = 2.4 Hz, 1H), 2.74 (dd, *J* = 4.9, 3.9 Hz, 1H), 1.70 (dd, *J* = 14.0, 2.1 Hz, 1H), 1.38 (ddd, *J* = 13.7, 11.2, 2.0 Hz, 1H), 1.22 (d, *J* = 6.3 Hz, 3H), 1.23 – 1.12 (m, 3H), 1.11 – 1.06 (m, 18H). **^{13}C{^{1}H} NMR (101 MHz, $CDCl_3$):** δ 102.5, 74.3, 72.1, 66.4, 56.2, 56.1, 44.3, 38.4, 20.8, 18.4, 13.1. **IR (film):** 3545, 2943, 2893, 2866, 1465, 1383, 1327, 1314, 1297, 1246, 1215, 1164, 1103, 1070, 1054, 1017, 999, 967, 919, 886, 842, 816, 795, 681, 654, 547, 484, 464 cm^{-1}. **HRMS-ESI *m/z*:** $[M+Na]^+$ calcd for $C_{18}H_{36}O_5SiNa$ 383.2224; found 383.2224.

(2*R*,3*R*,4*S*,6*R*)-2-Methoxy-6-methyl-3-((triisopropylsilyl)oxy)-4-vinyltetrahydro-2*H*-pyran-4-ol (11). In a 250-mL two-necked flask, a solution of epoxide **10** (1.36 g, 3.77 mmol) in diethyl ether (20 mL) was slowly added to lithium aluminum hydride (345 mg, 9.09 mmol) in diethyl ether (60 mL) at 0 °C. Once the addition was complete, the cooling bath was removed and stirring was continued for 2 h at room temperature. The mixture was cooled on ice and the reaction was quenched by the addition of ethyl acetate (5 mL). Saturated aqueous Rochelle's salt solution (80 mL) was introduced and stirring continued for 14 h at room temperature to give a clear biphasic mixture. The aqueous phase was extracted with ethyl acetate (10 × 20 mL) and the combined organic layers were dried over anhydrous sodium sulfate. The drying agent was filtered off and the solvent was removed under reduced pressure. Purification of the crude product by flash chromatography (dichloromethane/methanol, 15:1) furnished the title compound as a colorless oil (710 mg, 91% yield), which crystallized upon standing at −20 °C. $[\alpha]_D^{20}$ = −43.3 (*c* 0.58, $CHCl_3$). **^{1}H NMR (400 MHz, $CDCl_3$):** δ 4.52 (d, *J* = 7.7 Hz, 1H), 4.02 (dqd, *J* = 12.6, 6.3, 2.3 Hz, 1H), 3.65 (q, *J* = 6.6 Hz, 1H), 3.59 (d, *J* = 7.7 Hz, 1H), 3.55 (s, 3H), 3.22 – 1.92 (br s, 3H), 1.55 (dd, *J* = 13.8, 2.3 Hz, 1H), 1.38 (dd, *J* = 13.7, 11.1 Hz, 1H), 1.27 (d, *J* = 6.6 Hz, 3H), 1.23 (d, *J* = 6.3 Hz, 3H). **^{13}C{^{1}H} NMR (101 MHz, $CDCl_3$):** δ 102.2, 73.8, 73.6, 72.0, 67.2, 57.1, 39.4, 21.0, 18.2. **IR (film):** 3416, 2973, 2934, 2845, 1641, 1447, 1384, 1327, 1277, 1210, 1162, 1125, 1072, 1048, 1030, 969, 930, 893, 865, 802, 730, 674, 528, 491 cm^{-1}. **HRMS-ESI *m/z*:** $[M-H]^-$ calcd for $C_9H_{17}O_5$ 205.1082; found 205.1082.

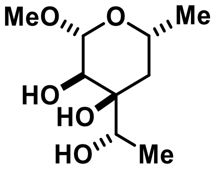

Methyl β-D-aldgaropyranoside (12). A solution of phosgene (20% *w/w* in toluene, 2.6 mL, 4.9 mmol) was added over 5 min to a vigorously stirred solution of triol **11** (823 mg, 3.99 mmol) in dichloromethane (10 mL) and pyridine (10 mL) at 0°C. The white suspension was vigorously stirred for 2 h at this temperature before it was diluted with dichlormethane (25 mL) and washed with aqueous HCl (2 M, 1 × 20 mL, 2 × 10 mL). The aqueous phases were extracted with chloroform (5 × 15 mL) and the combined organic layers were dried over anhydrous sodium sulfate. The drying agent was filtered off and the filtrate was concentrated

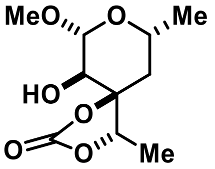

under reduced pressure. Remaining pyridine was largely removed by co-evaporation with toluene. The residue was purified by flash column chromatography (hexanes/EtOAc, 3:2 → 1:1 → 1:2) to give the title compound as colorless crystals (737 mg, 80% yield). Single crystals suitable for X-ray diffraction were obtained by recrystallization from diethyl ether/dichloromethane (4:1). **mp** = 175 – 176 °C. $[\alpha]_D^{23}$ = −38.1 (*c* 1.0, MeOH). **^{1}H NMR (400 MHz, CDCl$_3$):** δ 4.51 (d, *J* = 7.8 Hz, 1H), 4.38 (q, *J* = 6.6 Hz, 1H), 3.95 (dqd, *J* = 12.6, 6.3, 2.2 Hz, 1H), 3.56 (s, 3H), 3.45 (d, *J* = 7.1 Hz, 1H), 2.42 (br s, 1H), 1.86 (dd, *J* = 14.3, 2.2 Hz, 1H), 1.59 – 1.51 (m, 4H), 1.27 (d, *J* = 6.3 Hz, 3H). **^{13}C{^{1}H} NMR (101 MHz, CDCl$_3$):** δ 154.1, 101.8, 84.6, 81.4, 71.2, 67.4, 57.4, 41.0, 20.7, 13.7. **IR (film):** 3459, 2977, 2937, 2849, 1788, 1448, 1385, 1360, 1332, 1286, 1227, 1206, 1160, 1120, 1070, 1049, 1021, 955, 936, 817, 773, 731, 680, 620, 608, 569, 544, 516, 475 cm^{-1}. **HRMS-ESI *m/z*:** $[M+Na]^+$ calcd for $C_{10}H_{16}O_6Na$ 255.0839; found 255.0834. The analytical data are in agreement with those reported in the literature.[5]

Methyl 2-O-acetyl-β-D-aldgaropyranoside (13a). A solution of phosgene (20% *w/w* in toluene, 2.4 mL) was added to a vigorously stirred solution of triol **11** (617 mg, 2.99 mmol) in dichloromethane (7.5 mL) and pyridine (7.5 mL) at 0°C. The resulting white suspension was vigorously stirred for 2 h at this temperature before it was diluted with dichloromethane (25 mL) and washed with aqueous HCl (2 M, 2 × 15 mL). The aqueous phases were extracted with dichloromethane (4 × 10 mL) and the combined organic layers were dried over anhydrous sodium sulfate. The drying agent was filtered off and the filtrate was concentrated under reduced pressure. Remaining pyridine was removed by co-evaporation with toluene (8 mL).

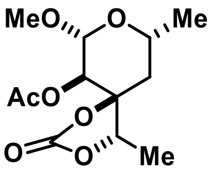

Triethylamine (1.3 mL, 9.3 mmol), 4-dimethylaminopyridine (73.6 mg, 0.602 mmol) and acetic anhydride (1.5 mL, 16 mmol) were added to a solution of this material in dichloromethane (30 mL) at 0°C. The resulting mixture was stirred for 3 h at room temperature before it was diluted with dichloromethane (15 mL) and washed with saturated aqueous sodium bicarbonate solution (25 mL). The aqueous phase was extracted with dichloromethane (5 × 10 mL) and the combined organic layers were dried over anhydrous sodium sulfate. The drying agent was filtered off and the solvent was removed under reduced pressure. Purification of the residue by flash chromatography (hexanes/EtOAc, 3:1 → 2:1) furnished the title compound as a white, crystalline solid (482 mg, 59% yield). **mp** = 112-113 °C. $[\alpha]_D^{20}$ = −74.8 (*c* 1.2, $CHCl_3$). **^{1}H NMR (400 MHz, CDCl$_3$):** δ 4.91 (d, *J* = 7.9 Hz, 1H), 4.57 (d, *J* = 7.9 Hz, 1H), 4.39 (q, *J* = 6.8 Hz, 1H), 3.97 (dqd, *J* = 12.4, 6.2, 2.2 Hz, 1H), 3.48 (s, 3H), 2.12 (s, 3H), 1.91 (dd, *J* = 14.3, 2.2 Hz, 1H), 1.63 (dd, *J* = 14.3, 11.2 Hz, 1H), 1.36 (d, *J* = 6.8 Hz, 3H), 1.29 (d, *J* = 6.2 Hz, 3H). **^{13}C{^{1}H} NMR (101 MHz, CDCl$_3$):** δ 169.4, 153.7, 100.8, 85.4, 81.3, 70.0, 67.0, 57.2, 41.3, 21.1, 20.6, 13.3. **IR (film):** 2975, 2939, 2915, 2881, 2849, 1830, 1803, 1753, 1448, 1396, 1378, 1331, 1308,

1281, 1234, 1218, 1205, 1179, 1161, 1145, 1119, 1084, 1070, 1053, 1018, 1007, 956, 938, 914, 824, 773, 687, 615, 563, 548 cm^{-1}. **HRMS-ESI *m/z*:** $[M+Na]^+$ calcd for $C_{12}H_{18}O_7Na$ 297.0945; found 297.0939.

1,2-Di-O-acetyl-D-aldgaropyranose (13b). In a 10-mL round-bottom flask, a solution of concentrated sulfuric acid in acetic anhydride (1:99 *v/v*, 6.0 mL) was added to a solution of methyl glycoside **13a** (482 mg, 1.76 mmol) in acetic anhydride (6.0 mL) at 0°C. After 5 min, the ice bath was removed and the mixture stirred for 1 h at room temperature. For work up, the mixture was diluted with ethyl acetate (30 mL) and carefully poured onto ice-cold water (25 mL) in a separatory funnel. After careful shaking of the two layers, saturated aqueous sodium bicarbonate solution was slowly added until all acid was destroyed (ca. 40 mL). After the gas formation had ceased, the layers were separated and the aqueous phase was extracted with ethyl acetate (6 × 10 mL). The combined organic layers were dried over anhydrous sodium sulfate, the drying agent was filtered off, and the filtrate was concentrated under reduced pressure. Most of the remaining acetic anhydride was destroyed by co-evaporation with ethanol (2 × 2 mL) followed by azeotropic removal with toluene (2 mL). Purification of the residue by flash chromatography (hexanes/EtOAc, 3:1 → 2:1) furnished the title compound as a colorless foam after another co-evaporation with toluene/ethanol (2 × 10 mL) and drying at 10^{-3} mbar (517 mg, 98% yield; α/β = 2:3). $[\alpha]_D^{20}$ **=** +11.4 (*c* 0.79, $CHCl_3$). **^{1}H NMR (400 MHz, $CDCl_3$):** signals of the α-anomer: δ 6.30 (d, *J* = 4.0 Hz, 1H), 5.00 (d, *J* = 4.0 Hz, 1H), 4.45 – 4.35 (m, 1H), 4.35 (q, *J* = 6.8 Hz, 1H), 2.16 (s, 3H), 2.068 (s, 3H), 2.03 – 1.90 (m, 1H), 1.72 – 1.62 (m, 1H), 1.36 (d, *J* = 6.7 Hz, 3H), 1.25 (d, *J* = 6.3 Hz, 3H); signals of the β-anomer: δ 5.87 (d, *J* = 8.1 Hz, 1H), 5.05 (d, *J* = 8.1 Hz, 1H), 4.41 (q, *J* = 6.8 Hz, 1H), 4.17 – 4.07 (m, 1H), 2.10 (s, 3H), 2.074 (s, 3H), 2.03 – 1.92 (m, 1H), 1.72 – 1.62 (m, 1H), 1.38 (d, *J* = 6.8 Hz, 3H), 1.29 (d, *J* = 6.2 Hz, 3H). **^{13}C{^{1}H} NMR (101 MHz, $CDCl_3$):** δ 170.0, 169.5, 169.2, 169.1, 153.5, 153.3, 91.7, 88.8, 85.0, 82.4, 81.7, 81.1, 69.1, 68.1, 67.2, 63.5, 40.9, 40.5, 21.2, 21.0, 20.9, 20.6, 20.5, 13.6, 13.4. **IR (film):** 2983, 2936, 1805, 1755, 1432, 1377, 1309, 1283, 1217, 1158, 1144, 1120, 1078, 1063, 1015, 917, 850, 811, 772, 689, 606, 559, 534 cm^{-1}. **HRMS-ESI *m/z*:** $[M+Na]^+$ calcd for $C_{13}H_{18}O_8Na$ 325.0894; found 325.0889.

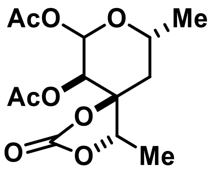

2-O-Acetyl-D-aldgaropyranose (S1). Benzylamine (0.91 mL, 8.3 mmol) was added to a solution of acetate **13b** (503 mg, 1.66 mmol) in THF (14 mL). The resulting mixture was stirred for 17 h at room temperature before it was diluted with ethyl acetate (25 mL) and washed with aqueous HCl (1 M, 25 mL). The aqueous phase was extracted with ethyl acetate (4 × 10 mL) and the combined organic layers were dried over anhydrous sodium sulfate. The drying agent was filtered off and the filtrate was concentrated under reduced pressure. Purification of the residue by flash chromatography (hexanes/EtOAc, 1:1) furnished the title compound as a white solid (303 mg, 70% yield; α/β = 1:15). **mp** = 144 – 146 °C.$[\alpha]_D^{20}$ **=** −39.3 (*c* 0.75, $CHCl_3$). **^{1}H NMR**

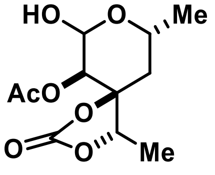

(400 MHz, CDCl$_3$, signals of the *β*-anomer only): δ 4.92 (dd, *J* = 9.1, 7.9 Hz, 1H), 4.79 (d, *J* = 7.9 Hz, 1H), 4.41 (q, *J* = 6.7 Hz, 1H), 4.03 (dqd, *J* = 12.5, 6.2, 2.2 Hz, 1H), 3.08 (d, *J* = 9.1 Hz, 1H), 2.17 (s, 3H), 1.93 (dd, *J* = 14.4, 2.2 Hz, 1H), 1.66 (dd, *J* = 14.4, 11.3 Hz, 1H), 1.37 (d, *J* = 6.7 Hz, 3H), 1.30 (d, *J* = 6.2 Hz, 3H). **^{13}C{^{1}H} NMR (101 MHz, CDCl$_3$,** signals of the *β*-anomer only)**:** δ 170.9, 153.4, 94.3, 85.0, 81.0, 72.3, 67.5, 41.3, 21.1, 20.7, 13.4. **IR (film):** 3416, 2978, 2924, 2855, 1790, 1750, 1639, 1448, 1431, 1378, 1326, 1284, 1228, 1157, 1115, 1069, 1051, 1013, 961, 938, 914, 871, 828, 774, 733, 688, 645, 616, 602, 561, 535, 508, 488, 460, 430 cm^{-1}. **HRMS-ESI *m/z*:** [M+Na]$^+$ calcd for $C_{11}H_{16}O_7Na$ 283.0788; found 283.0786.

1-Deoxy-1-fluoro-2-O-acetyl-D-aldgaropyranoside (13c). In a 10-mL Schlenk tube, a solution of diethylaminosulfur trifluoride (0.13 M in dichloromethane, 3.1 mL, 0.40 mmol) was added to a solution of lactol **13a** (51.0 mg, 0.196 mmol) in dichloromethane (1.3 mL) at −15 °C. After stirring for 1.5 h at this temperature, the reaction mixture was diluted with *tert*-butyl methyl ether (10 mL) and washed with saturated aqueous sodium bicarbonate solution (10 mL). The aqueous phase was extracted with *tert*-butyl methyl ether (4 × 10 mL). The combined organic layers were dried over anhydrous sodium sulfate, the drying agent was filtered off, and the solvent was removed under reduced pressure. The residue was purified by flash chromatography (hexanes/EtOAc, 2:1) to furnish the title compound as a colorless gum (40.3 mg, 78% yield; 1:2 mixture of α/β anomers), which formed a white wax upon standing. For analytical purposes, an aliquot was re-subjected to flash chromatography to give a pure sample of the β-anomer. The signals of the α-anomer were then assigned from the NMR spectrum of the mixture containing both anomers. Spectral data of the α-anomer: **^{1}H NMR (400 MHz, CDCl$_3$):** δ 5.71 (dd, *J* = 53.2, 3.3 Hz, 1H), 4.82 (dd, *J* = 23.4, 3.3 Hz, 1H), 4.52 – 4.40 (m, 1H), 4.36 (q, *J* = 6.7 Hz, 1H), 2.19 (s, 3H), 2.02 (dd, *J* = 14.2, 2.4 Hz, 1H), 1.70 (dd, *J* = 14.4, 11.5 Hz, 1H), 1.37 (d, *J* = 6.7 Hz, 3H), 1.28 (d, *J* = 6.2 Hz, 3H). **^{13}C{^{1}H} NMR (101 MHz, CDCl$_3$):** δ 169.7, 153.3, 103.0 (d, *J* = 232 Hz), 82.1, 81.4, 68.0 (d, *J* = 25.1 Hz), 63.3 (d, *J* = 3.5 Hz), 40.2, 20.8, 20.3, 13.7. **^{19}F{^{1}H} NMR (282 MHz, CDCl$_3$):** δ −147.2. Spectral data of the β-anomer: **^{1}H NMR (400 MHz, CDCl$_3$):** δ 5.40 (dd, *J* = 52.6, 7.2 Hz, 1H), 5.03 (dd, *J* = 11.3, 7.2 Hz, 1H), 4.43 (qd, *J* = 6.8, 1.7 Hz, 1H), 4.16 – 4.05 (m, 1H), 2.16 (s, 3H), 1.95 (dt, 14.5, 2.4 Hz, 1H), 1.71 (dd, *J* = 14.5, 11.3 Hz, 1H), 1.39 (d, *J* = 6.8 Hz, 3H), 1.35 (d, *J* = 6.2 Hz, 3H). **^{13}C{^{1}H} NMR (101 MHz, CDCl$_3$):** δ 169.1, 153.2, 107.0 (d, *J* = 214 Hz), 81.1 (d, *J* = 8.7 Hz), 81.0, 69.8 (d, *J* = 24.1 Hz), 68.0 (d, *J* = 4.2 Hz), 40.7, 20.9, 20.4, 13.6. **^{19}F{^{1}H} NMR (282 MHz, CDCl$_3$):** δ −147.3. **IR (film):** 2983, 2924, 2851, 1803, 1756, 1450, 1374, 1285, 1228, 1180, 1158, 1147, 1079, 1064, 1022, 943, 919, 828, 803, 773, 691 cm^{-1}. **HRMS-ESI *m/z*:** [M+Na]$^+$ calcd for $C_{11}H_{15}FO_6Na$ 285.0745; found 285.0743.

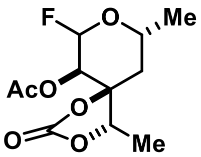

1-Deoxy-1-(phenylthio)-2-O-acetyl-D-aldgaropyranoside (13d). In a 10-mL Schlenk tube, tin(IV) chloride (1 M in dichloromethane, 0.25 mL, 0.25 mmol) was added to a solution of acetate **13b** (83.8 mg, 0.277 mmol) and thiophenol (50 µL, 0.49 mmol) in dichloromethane (3.0 mL) at 0 °C (ice bath). After stirring for 1.5 h at this temperature, the mixture was diluted with *tert*-butyl methyl ether (10 mL) and washed with saturated aqueous sodium carbonate solution (10 mL). The aqueous phase was extracted with *tert*-butyl methyl ether (3 × 5 mL). The combined organic layers were dried over anhydrous sodium sulfate, the drying agent was filtered off, and the solvent was removed under reduced pressure. The residue was purified by flash chromatography (hexanes/EtOAc, 2:1) to furnish the title compound as yellowish gum (90.0 mg, 83% yield; ca. 2:3 mixture of α/β anomers). Characteristic data: **^{1}H NMR (400 MHz, $CDCl_3$):** δ 7.49 – 7.41 (m, 2H), 7.34 – 7.23 (m, 3H), 5.84 (d, J = 6.1 Hz, 1H, *single anomer*), 5.05 – 4.95 (m, 1H), 4.75 – 4.65 (m, 1H, *single anomer*), 4.43 – 4.33 (m, 1H), 4.03 – 3.93 (m, 1H, *single anomer*), 2.18 (s, 3H, *single anomer*), 2.16 (s, 3H, *single anomer*), 2.02 – 1.92 (m, 1H), 1.74 – 1.63 (m, 1H), 1.40 (d, J = 6.7 Hz, 3H, *single anomer*), 1.38 (d, J = 6.8 Hz, 3H, *single anomer*). 1.30 (d, J = 6.3 Hz, 3H, *single anomer*), 1.27 (d, J = 6.3 Hz, 3H, *single anomer*). **HRMS-ESI *m/z*:** $[M+Na]^+$ calcd for $C_{17}H_{20}O_6SNa$ 375.0873; found 375.0870.

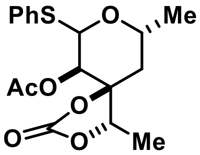

D-Aldgaropyranose. Methyl glycoside **12** (419 mg, 1.80 mmol) was dissolved in trifluoroacetic acid and tetrahydrofuran (1:2 *v/v*, 8.5 mL) and the reaction mixture was stirred for 4 h at 100 °C. The mixture was cooled to room temperature and the solvents were removed under reduced pressure (10^{-3} mbar, 30 – 40 °C). The resulting yellowish gum was dissolved in ethyl acetate (30 mL), the solution dried over anhydrous sodium sulfate, and concentrated under reduced pressure. The residue was purified by flash chromatography (dichloromethane/methanol, 25:1 → 20:1) to furnish the title compound as a white solid (263 mg, 67% yield; ca. 1:8 mixture of α/β anomers). **mp** 169 – 170 °C. $[\alpha]_D^{20}$ = −7.3 (*c* 0.82, MeOH). **^{1}H NMR (400 MHz, [D_4]-methanol):** δ 4.72 (d, J = 7.8 Hz, 1H), 4.49 (q, J = 6.6 Hz, 1H), 3.90 (dqd, J = 12.5, 6.2, 2.1 Hz, 1H), 3.35 (d, J = 7.7 Hz, 1H), 1.92 (dd, J = 14.5, 2.2 Hz, 1H), 1.61 (dd, J = 14.5, 11.3 Hz, 1H), 1.55 (d, J = 6.6 Hz, 3H), 1.22 (d, J = 6.2 Hz, 3H). **^{13}C{^{1}H} NMR (101 MHz, $CDCl_3$):** δ 156.5, 96.3, 87.3, 83.1, 72.4, 68.3, 41.9, 21.0, 13.7. **IR (film):** 3417, 2979, 2917, 1777, 1550, 1449, 1384, 1324, 1289, 1219, 1157, 1140, 1114, 1072, 1023, 960, 936, 902, 824, 774, 681, 629, 594 cm^{-1}. **HRMS-ESI *m/z*:** $[M+Na]^+$ calcd for $C_9H_{14}O_6Na$ 241.0683; found 241.0680.

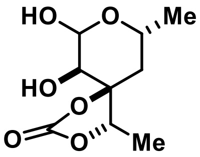

1-Deoxy-1-(pyrimidin-2-ylthio)-2-O-acetyl-β-D-aldgaropyranoside (13e). In a 25-mL Schlenk

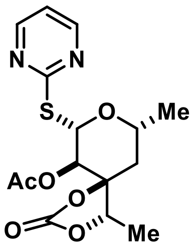

tube, triethylphosphine (0.38 mL, 2.6 mmol) was added to a mixture of lactol **12** (286 mg, 1.31 mmol) and bis(pyrimidin-2-yl) disulfide (350 mg, 1.57 mmol)[6] in acetonitrile (4.5 mL) at 0 °C (ice bath). The mixture immediately turned yellow with formation of a precipitate. After stirring for 2 h at 0 °C, the mixture was diluted with chloroform (25 mL) and washed with saturated aqueous solutions of sodium chloride and sodium carbonate (25 mL each). The aqueous phases were combined and extracted with chloroform (4 × 10 mL). The organic phases were dried over anhydrous sodium sulfate, the drying agent was filtered off, and the solvent was removed under reduced pressure. The residue was purified by flash chromatography (hexanes/acetone, 3:2) to furnish a yellow syrup (316 mg, 77% yield), which was used in the next step without full characterization.

Triethylamine (0.38 mL, 2.7 mmol), 4-(dimethylamino)pyridine (31 mg, 0.25 mmol) and acetic anhydride (0.19 mL, 2.0 mmol) were added at room temperature to a solution of this alcohol in dichloromethane (9.5 mL). After stirring for 1 h, the mixture was diluted with chloroform (15 mL) and washed with saturated aqueous sodium bicarbonate solution (20 mL). The aqueous phase was extracted with chloroform (5 × 10 mL) and the combined organic layers were dried over anhydrous sodium sulfate. The drying agent was filtered off and the solvent was removed under reduced pressure. The residue was purified by flash chromatography (hexanes/EtOAc, 1:1 → 2:3) to furnish the title compound as a pale yellowish solid (255 mg, 71% yield). **mp** 179 – 180 °C. $[\alpha]_D^{20}$ = +169.7 (*c* 1.0, $CHCl_3$). **^{1}H NMR (400 MHz, $CDCl_3$):** δ 8.56 (d, *J* = 4.8 Hz, 2H), 7.01 (t, *J* = 4.9 Hz, 1H), 6.93 (d, *J* = 6.3 Hz, 1H), 5.19 (d, *J* = 6.3 Hz, 1H), 4.53 – 4.42 (m, 1H), 4.39 (q, *J* = 6.7 Hz, 1H), 2.09 (s, 3H), 1.99 (dd, *J* = 14.4, 2.2 Hz, 1H), 1.71 (dd, *J* = 14.4, 11.4 Hz, 1H), 1.42 (d, *J* = 6.7 Hz, 3H), 1.24 (d, *J* = 6.3 Hz, 3H). **^{13}C{^{1}H} NMR (101 MHz, $CDCl_3$):** δ 170.8, 169.4, 157.7, 152.9, 117.4, 82.8, 81.3, 80.1, 68.6, 63.7, 40.8, 21.0, 20.6, 13.6. **IR (film):** 2979, 2937, 1804, 1752, 1565, 1551, 1384, 1285, 1226, 1203, 1181, 1144, 1072, 1015, 917, 811, 772, 751, 639, 605, 532 cm^{-1}. **HRMS-ESI *m/z*:** $[M+Na]^+$ calcd for $C_{15}H_{18}N_2O_6SNa$ 377.0778; found 377.0774.

1-Deoxy-1-(phenylsulfoxyl)-2-O-acetyl-D-aldgaropyranoside (13f). In a 10-mL Schlenk tube,

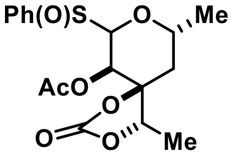

3-chloroperbenzoic acid (6.0 mg, 0.024 mmol) was added to a solution of thioglycoside **13d** (7.0 mg, 0.020 mmol) in dichloromethane (0.2 mL) at −70 °C. The mixture was gradually warmed to −40 °C over 1.5 h and then directly subjected to flash chromatography (hexanes/EtOAc, 2:1 → 1:2) to give three fractions containing separate isomers of the title compound. While the first fraction predominantly contained the byproduct 3-chlorobenzoic acid, washing of the remaining two fractions with saturated aqueous sodium carbonate solution furnished clean samples of single diastereomers of the title compound, which showed the following

spectral data: Isomer with R_f = 0.19 (hexanes/EtOAc, 1:1), colorless gum (2.0 mg, 27% yield). **^{1}H NMR (400 MHz, $CDCl_3$):** δ 7.74 – 7.66 (m, 2H), 7.58 – 7.50 (m, 3H), 5.30 (d, *J* = 6.0 Hz, 1H), 4.63 (d, *J* = 6.1 Hz, 1H), 4.46 (q, *J* = 6.7 Hz, 1H), 4.00 – 3.91 (m, 1H), 2.24 (s, 3H), 2.00 (dd, *J* = 14.3, 2.1 Hz, 1H), 1.68 (dd, *J* = 14.3, 11.3 Hz, 1H), 1.44 (d, *J* = 6.6 Hz, 3H), 0.92 (d, *J* = 6.2 Hz, 3H). Isomer with R_f = 0.12 (hexanes/EtOAc, 1:1), white solid (1.7 mg, 23% yield). **^{1}H NMR (400 MHz, $CDCl_3$):** δ 7.67 – 7.61 (m, 2H), 7.55 – 7.50 (m, 3H), 5.50 (d, *J* = 9.7 Hz, 1H), 4.43 (q, *J* = 6.8 Hz, 1H), 4.16 (d, *J* = 9.7 Hz, 1H), 3.78 (dqd, *J* = 12.4, 6.2, 2.2 Hz, 1H), 2.23 (s, 3H), 1.91 (dd, *J* = 14.4, 2.2 Hz, 1H), 1.75 (dd, *J* = 14.4, 11.2 Hz, 1H), 1.44 (d, *J* = 6.8 Hz, 3H), 1.15 (d, *J* = 6.2 Hz, 3H).

Trichloroacetimidate 13g. Trichloroacetonitrile (0.56 mL, 5.6 mmol) and DBU (42 µL, 0.28 mmol) were sequentially added to a solution of lactol **S1** (292 mg, 1.12 mmol) in dichloromethane (8.0 mL) at 0°C. The originally colorless solution turned slightly brown upon the addition of DBU and the color intensified over the course of the reaction. After 1 h, the mixture was warmed to room temperature before it was concentrated under reduced pressure. The residue was immediately subjected to flash chromatographic purification (hexanes/EtOAc, 3:1 + 0.1 vol-% of triethylamine) to give the title compound as a pale yellowish/white solid (416 mg, 92% yield; *α*/*β* = 1:3). **mp** = 123 – 124 °C.$[\boldsymbol{\alpha}]_{\mathbf{D}}^{\mathbf{20}}$ **=** +12.2 (*c* 0.97, $CHCl_3$). **^{1}H NMR (400 MHz, $CDCl_3$):** signals of the *α*-anomer: δ 8.49 (s, 1H), 6.55 (d, *J* = 4.1 Hz, 1H), 5.04 (d, *J* = 4.1 Hz, 1H), 4.55 – 4.45 (m, 1H), 4.36 (q, *J* = 6.6, 6.1 Hz, 1H), 2.07 (s, 3H), 2.04 (dd, 14.4, 2.2 Hz, 1H), 1.73 (dd, *J* = 14.4, 11.8 Hz, 1H), 1.38 (d, *J* = 6.7 Hz, 3H), 1.28 (d, *J* = 6.3 Hz, 3H); signals of the *β*-anomer: δ 8.70 (s, 1H), 6.00 (d, *J* = 8.2 Hz, 1H), 5.26 (d, *J* = 8.2 Hz, 1H), 4.44 (q, *J* = 6.8 Hz, 1H), 4.18 (dqd, *J* = 12.4, 6.2, 2.2 Hz, 1H), 2.08 (s, 3H), 1.98 (dd, *J* = 14.4, 2.2 Hz, 1H), 1.74 (dd, *J* = 14.4, 11.3 Hz, 1H), 1.41 (d, *J* = 6.8 Hz, 3H), 1.33 (d, *J* = 6.2 Hz, 3H). **^{13}C{^{1}H} NMR (101 MHz, $CDCl_3$):** δ 168.9, 161.1, 153.4, 95.5, 92.5, 85.3, 81.8, 81.4, 81.2, 68.9, 68.3, 67.6, 63.8, 41.0, 40.4, 21.0, 20.62, 20.57, 20.52, 13.6, 13.4. **IR (film):** 3336, 2982, 2936, 1805, 1757, 1676, 1447, 1427, 1380, 1336, 1286, 1223, 1158, 1084, 1062, 1012, 968, 935, 914, 836, 797, 771, 757, 688, 646, 610, 543 cm^{-1}. **HRMS-ESI *m/z*:** $[M+Na]^+$ calcd for $C_{13}H_{16}NO_7Cl_3Na$ 425.9885; found 425.9885.

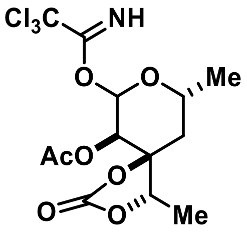

Synthesis of the D-Mycinopyranosyl Donor

6-Bromo-6-deoxy-D-isoascorbic acid (S2). In a round-bottom flask, D-isoascorbic acid (**21**) (15.8 g, 89.6 mmol) was added to a solution of hydrogen bromide in acetic acid (33% *w/w*, 70 mL) at room temperature. The flask was covered with aluminum foil and the mixture stirred for 16 h before it was carefully poured onto ice-cold water (350 mL). Stirring was then continued for 78 h at room temperature. All volatile materials were removed under high vacuum at 40 °C to give a very dark brown residue, which was subjected to flash chromatography (dichloromethane/methanol, 10:1); a second purification by flash chromatography (hexanes/acetone, 2:1) was necessary to obtain the title compound as a dark orange wax (18.1 g, 84% yield). $[\alpha]_D^{25}$ = −20.3 (*c* 0.92, MeOH). **^{1}H NMR (400 MHz, [D$_4$]-methanol):** δ 4.86 (d, *J* = 3.3 Hz, 1H), 4.12 (ddd, *J* = 8.0, 4.8, 3.3 Hz, 1H), 3.59 (dd, *J* = 10.7, 4.9 Hz, 1H), 3.47 (dd, *J* = 10.6, 7.7 Hz, 1H). **^{13}C{^{1}H} NMR (101 MHz, [D$_4$]-methanol):** δ 172.8, 154.5, 120.2, 77.9, 72.5, 32.9. **IR (film):** 3224, 1756, 1668, 1341, 1295, 1204, 1134, 1092, 1009, 844, 819, 759, 615, 517 cm^{-1}. **HRMS-ESI *m/z*:** [M+H]$^+$ calcd for $C_6H_8O_5Br$ 238.9550; found 238.9550.

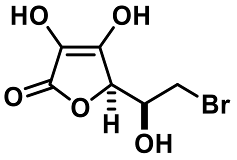

6-Bromo-6-deoxy-2,3-di-O-methyl-D-isoascorbic acid (22). In a round-bottom flask, a solution of trimethylsilyl diazomethane in diethyl ether (2 M, 100 mL, 200 mmol) was added via a dropping funnel over 30 min to a solution of enediol **S2** (11.5 g, 48.0 mmol) in toluene/methanol (7:2, 495 mL) at 0°C. After 10 min, the ice bath was removed and stirring continued for 4.5 d at room temperature. The reaction was quenched by the sequential, dropwise addition of acetic acid and methanol (both 18 mL) at 0°C. After stirring for 2 h at room temperature, the mixture was concentrated under reduced pressure and the residue subjected to flash chromatography (hexanes/EtOAc, 3:1 → 1:1) to give the title compound as a yellowish syrup (10.0 g, 78% yield). $[\alpha]_D^{20}$ = −17.1 (*c* 1.1, $CHCl_3$). **^{1}H NMR (400 MHz, $CDCl_3$):** δ 4.79 (br d, *J* = 4.5 Hz, 1H), 4.17 (s, 3H), 4.14 – 4.08 (m, 1H), 3.85 (s, 3H), 3.59 – 3.48 (m, 2H). **^{13}C{^{1}H} NMR (101 MHz, $CDCl_3$):** δ 168.6, 157.5, 123.3, 75.3, 71.6, 60.5, 59.9, 32.8. **IR (film):** 3428, 2956, 1765, 1673, 1463, 1434, 1330, 1233, 1213, 1136, 1099, 1052, 984, 956, 768 cm^{-1}. **HRMS-ESI *m/z*:** [M+Na]$^+$ calcd for $C_8H_{11}O_5BrNa$ 288.9682; found 288.9682.

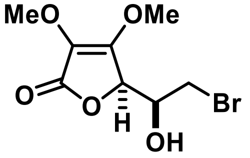

6-Deoxy-2,3-di-O-methyl-D-isoascorbic acid (S3). In a 100-mL two-necked flask, triethylamine (2.8 mL, 20 mmol) and a solution of bromide **S2** (2.66 g, 9.96 mmol) in methanol (20 mL) were added to a vigorously stirred suspension of Pd/C (10% *w/w*, 211 mg, 0.198 mmol) in methanol (20 mL) under hydrogen atmosphere. After vigorous stirring for 3 h at room temperature, the mixture was filtered through a short plug of Celite®, which was rinsed with dichloromethane (5 × 10 mL). The combined filtrates were

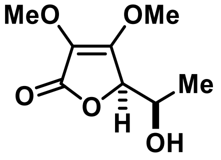

concentrated and the residue was loaded onto Celite®. Purification by flash chromatography (hexanes/EtOAc, 1:1) gave the title compound as a colorless syrup (1.67 g, 89% yield). $[\alpha]_D^{20}$ = +15.6 (*c* 1.2, $CHCl_3$). **^{1}H NMR (400 MHz, $CDCl_3$):** δ 4.60 (d, *J* = 3.9 Hz, 1H), 4.15 (s, 3H), 4.07 (qd, *J* = 6.5, 3.8 Hz, 1H), 3.85 (s, 3H), 2.08 (br s, 1H), 1.22 (d, *J* = 6.6 Hz, 3H). **^{13}C{^{1}H} NMR (101 MHz, $CDCl_3$):** δ 169.2, 158.0, 123.2, 78.3, 68.0, 60.6, 59.7, 17.0. **IR (film):** 3418, 2979, 2942, 2841, 1758, 1673, 1463, 1380, 1346, 1332, 1231, 1214, 1183, 1132, 1093, 1049, 1022, 1005, 985, 955, 936, 849, 803, 769, 722, 671 cm^{-1}. **HRMS-ESI *m/z*:** $[M+Na]^+$ calcd for $C_8H_{12}O_5Na$ 211.0577; found 211.0578.

(3*R*,4*R*,5*R*)-5-((*R*)-1-hydroxyethyl)-3,4-dimethoxydihydrofuran-2(3*H*)-one (23). A 100-mL

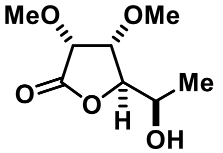

steel autoclave with glass inlay and magnetic stirring bar was charged with solutions of alkene **S3** (1.05 g, 5.58 mmol) and [Rh(dppb)(cod)]BF_4 (426 mg, 0.588 mmol) in dichloromethane (35 mL and 20 mL) under an argon atmosphere. Then, a hydrogen atmosphere was applied (100 bar total pressure) and the mixture was stirred for 24 h at room temperature. For work up, the pressure was carefully released and the mixture was concentrated under reduced pressure. The residue was loaded onto Celite® and subjected to flash chromatography (hexanes/acetone, 5:2) to furnish the title compound as a white, crystalline solid (1.00 g, 94% yield). $[\alpha]_D^{20}$ = +17.3 (*c* 1.1, $CHCl_3$). **^{1}H NMR (400 MHz, $CDCl_3$):** δ 4.29 (dd, *J* = 3.3, 2.4 Hz, 1H), 4.21 (d, *J* = 5.8 Hz, 1H), 4.17 – 4.09 (m, 2H), 3.65 (s, 3H), 3.47 (s, 3H), 1.94 (br s, 1H), 1.31 (d, *J* = 6.6 Hz, 3H). **^{13}C{^{1}H} NMR (101 MHz, $CDCl_3$):** δ 173.2, 85.4, 76.4, 75.4, 66.8, 59.4, 58.0, 18.9. **IR (film):** 3496, 2997, 2977, 2941, 2904, 2884, 2840, 1760, 1463, 1449, 1393, 1258, 1199, 1155, 1142, 1110, 1066, 1041, 1011, 993, 983, 955, 933, 866, 841, 787, 758, 644, 564, 478 cm^{-1}. **HRMS-ESI *m/z*:** $[M+Na]^+$ calcd for $C_8H_{14}O_5Na$ 190.0836; found 190.0837. **mp** 129 – 130 °C.

1,4-Di-O-acetyl-D-mycinopyranose (24). In a 250-mL Schlenk flask with cooling jacket, a solution of

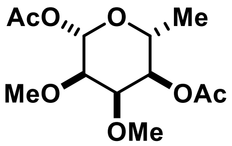

diisobutylaluminum hydride in hexanes (1.0 M, 16.5 mL, 23 mmol) was added over 10 min to a suspension of lactone **23** (876 mg, 4.61 mmol) in toluene (50 mL) at −78 °C. After complete addition, the mixture was stirred for 5 min at −78 °C and for another 4 h at −55 °C. The reaction was quenched by slow addition of ethyl acetate (10 mL) at −55 °C. Saturated aqueous Rochelle's salt solution (80 mL) and ethyl acetate (50 mL) were added and the mixture was vigorously stirred for 14 h at room temperature until a clean separation of the layers was reached. The aqueous phase was extracted with ethyl acetate (10 × 15 mL), the combined organic layers were dried over anhydrous sodium sulfate, the drying agent was filtered off, and the solvent was removed under reduced pressure to give a thick, colorless gum.

A solution of sulfuric acid in acetic anhydride (1:99 *v/v*, 5 mL) was added to a solution of this material in acetic anhydride (10 mL) at 0°C. The ice bath was removed and stirring continued at ambient temperature

for 40 min. The dark amber solution was diluted with ethyl acetate (20 mL) and poured onto ice-cold water (20 mL) in a separatory funnel. The layers were separated and the organic layer was washed with saturated aqueous sodium bicarbonate solution (20 mL). The combined aqueous phases were extracted with ethyl acetate (6 × 10 mL), the organic layers were dried over anhydrous sodium sulfate, the drying agent was filtered off and the filtrate was concentrated under reduced pressure. Remaining acetic anhydride was removed under high vacuum (10^{-2} mbar, warm water bath, ca. 3 h). Purification of the crude material by flash chromatography (hexanes/ethyl acetate, 4:1 → 3:1 → 5:2) furnished the title compound as a colorless, crystalline solid (606 mg, 48% yield; single anomer). **mp** = 99 – 101 °C. $[\alpha]_D^{20}$ = +28.0 (*c* 0.64, $CHCl_3$). **^{1}H NMR (400 MHz, $CDCl_3$):** δ 5.89 (d, *J* = 8.3 Hz, 1H), 4.45 (dd, *J* = 9.9, 2.6 Hz, 1H), 4.10 (dq, *J* = 10.0, 6.3 Hz, 1H), 4.00 (t, *J* = 2.6 Hz, 1H), 3.54 (s, 3H), 3.47 (s, 3H), 3.22 (dd, *J* = 8.3, 2.7 Hz, 1H), 2.134 (s, 3H), 2.126 (s, 3H), 1.18 (d, *J* = 6.2 Hz, 3H). **^{13}C{^{1}H} NMR (101 MHz, $CDCl_3$):** δ 170.4, 169.4, 92.0, 80.0, 76.5, 74.6, 68.5, 61.5, 58.7, 21.3, 21.1, 17.5. **IR (film):** 2983, 2940, 2834, 1755, 1451, 1370, 1224, 1173, 1139, 1100, 1060, 1000, 966, 935, 909, 886, 709, 562 cm^{-1}. **HRMS-ESI *m/z*:** $[M+Na]^+$ calcd for $C_{12}H_{20}O_7Na$ 299.1101; found 299.1101.

4-O-Acetyl-D-mycinopyranose (S4). Benzylamine (0.55 mL, 5.0 mmol) was added to a solution of acetate **24** (276 mg, 0.999 mmol) in THF (8.5 mL). The resulting mixture was stirred for 15 h at room temperature before it was diluted with ethyl acetate (20 mL) and washed with aqueous HCl (1 M, 20 mL). The aqueous phase was extracted with ethyl acetate (4 × 10 mL) and the combined organic layers were dried over anhydrous sodium sulfate. The drying agent was filtered off and the filtrate was concentrated under reduced pressure. Purification of the residue by flash chromatography (hexanes/EtOAc, 3:1 → 1:1) gave the title compound as a colorless gum (114 mg, 49% yield; *α*/*β* ≈ 3:4). $[\alpha]_D^{20}$ = +58.5 (*c* 1.2, $CHCl_3$). **^{1}H NMR (400 MHz, $CDCl_3$):** signals of the *α*-anomer: δ 5.23 – 5.14 (m, 1H), 4.47 (dt, *J* = 10.1, 2.2 Hz, 1H), 4.18 (dq, *J* = 10.1, 6.2 Hz, 1H), 4.06 – 4.03 (m, 1H), 3.57 (s, 3H), 3.47 (s, 3H), 3.30 (t, *J* = 2.8 Hz, 1H), 2.13 (s, 3H), 1.20 (t, *J* = 6.2 Hz, 3H); signals of the *β*-anomer: δ 5.01 (d, *J* = 7.8 Hz, 1H), 4.47 (dt, *J* = 10.2, 2.3 Hz, 1H), 4.00 (m, 1H), 3.98 (t, *J* = 2.8 Hz, 1H), 3.54 (s, 3H), 3.53 (s, 3H), 3.05 (dd, *J* = 7.9, 2.7 Hz, 1H), 2.13 (s, 3H), 1.19 (d, *J* = 6.3 Hz, 3H). **^{13}C{^{1}H} NMR (101 MHz, $CDCl_3$):** signals of the *α*-anomer: δ 170.3, 92.2, 78.9, 76.8, 74.1, 62.2, 60.9, 60.0, 21.1, 17.4; signals of the *β*-anomer: δ 170.3, 94.3, 82.1, 76.5, 74.1, 67.8, 61.6, 58.6, 21.1, 17.4. **IR (film):** 3464, 2982, 2937, 2835, 1740, 1453, 1375, 1236, 1199, 1158, 1131, 1097, 1081, 1047, 1003, 963, 921, 834, 796, 712, 603, 561, 528, 495 cm^{-1}. **HRMS-ESI *m/z*:** $[M+Na]^+$ calcd for $C_{10}H_{18}O_6Na$ 257.0996; found 257.0997.

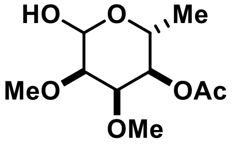

1-Deoxy-1-fluoro-4-O-acetyl-D-mycinopyranoside (25a). In a 60-mL Nalgene™ screw-capped

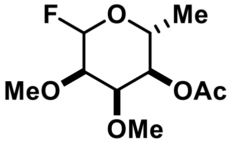

reaction vessel, hydrogen fluoride pyridine complex (70% HF *w/w*, 1.7 mL) was added to a solution of acetate **24** (316 mg, 1.14 mmol) in dichloromethane (12 mL) at 0 °C (ice bath). The reaction mixture, which turned pale yellowish upon the addition, was stirred for 2 h at 0 °C. For work-up, the cold reaction mixture was poured onto a saturated aqueous solution of sodium bicarbonate (30 mL). The layers were separated and the aqueous phase was extracted with dichloromethane (4 × 10 mL). The combined organic layers were dried over anhydrous sodium sulfate, the drying agent was filtered off, and the solvent was removed under reduced pressure. The residue was purified by flash chromatography (hexanes/EtOAc, 4:1 → 3:1) to furnish the anomers of the title compound inseparate fractions.

Analytical and spectroscopic data of the α-anomer: colorless oil, giving a white solid upon standing (184 mg, 68% yield); $[\alpha]_D^{20}$ = +122.0 (*c* 0.59, $CHCl_3$). **^{1}H NMR (400 MHz, $CDCl_3$):** δ 5.60 (ddt, *J* = 53.7, 3.2, 0.9 Hz, 1H), 4.54 (dd, *J* = 10.2, 2.5 Hz, 1H), 4.38 (dq, *J* = 10.3, 6.3 Hz, 1H), 4.04 – 4.01 (m, 1H), 3.56 (s, 3H), 3.49 (s, 3H), 3.31 (dt, *J* = 26.7, 3.2 Hz, 1H), 2.14 (s, 3H), 1.20 (d, *J* = 6.3 Hz, 3H). **^{13}C{^{1}H} NMR (101 MHz, $CDCl_3$):** δ 170.3, 104.3 (d, *J* = 234 Hz), 77.7 (d, *J* = 23.1 Hz), 75.4 (d, *J* = 1.7 Hz), 73.8, 63.7 (d, *J* = 2.5 Hz), 61.7, 57.5, 21.1, 17.0. **^{19}F{^{1}H} NMR (282 MHz, $CDCl_3$):** δ −144.9. **IR (film):** 2982, 2939, 2834, 1738, 1453, 1376, 1328, 1234, 1204, 1180, 1138, 1120, 1088, 1049, 992, 937, 891, 867, 832, 786, 713, 660, 549 cm^{-1}. **HRMS-ESI *m/z*:** $[M+Na]^+$ calcd for $C_{10}H_{17}FO_5Na$ 259.0952; found 259.0952.

Analytical and spectroscopic data of the β-anomer: colorless oil (48.9 mg, 18% yield); $[\alpha]_D^{20}$ = +39.4 (*c* 0.70, $CHCl_3$). **^{1}H NMR (400 MHz, $CDCl_3$):** δ 5.46 (dd, *J* = 54.1, 7.2 Hz, 1H), 4.52 (dd, *J* = 9.6, 2.6 Hz, 1H), 4.09 (dq, *J* = 9.5, 6.3 Hz, 1H), 3.96 (dt, *J* = 4.0, 2.8 Hz, 1H), 3.54 – 3.52 (m, 6H), 3.21 (ddd, *J* = 11.6, 7.2, 2.9 Hz, 1H), 2.13 (s, 3H), 1.25 (d, *J* = 6.2 Hz, 3H). **^{13}C{^{1}H} NMR (101 MHz, $CDCl_3$):** δ 170.3, 108.4 (d, *J* = 212 Hz), 80.5 (d, *J* = 20.6 Hz), 77.0 (d, *J* = 9.5 Hz), 74.05, 68.6 (d, *J* = 5.0 Hz), 61.5, 59.1 (d, *J* = 2.0 Hz), 21.1, 17.6. **^{19}F{^{1}H} NMR (282 MHz, $CDCl_3$):** δ −145.9. **IR (film):** 2984, 2937, 2835, 1743, 1453, 1373, 1313, 1234, 1202, 1138, 1100, 1074, 1051, 999, 966, 907, 709, 563, 485 cm^{-1}. **HRMS-ESI *m/z*:** $[M+Na]^+$ calcd for $C_{10}H_{17}FO_5Na$ 259.0952; found 259.0951.

4-O-Acetyl-D-mycinospyranosyl trichloroacetimidate (25b). Trichloroacetonitrile (0.17 mL,

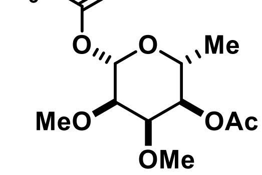

1.7 mmol) and DBU (12 µL, 80 µmol) were sequentially added to a solution of lactol **S4** (78.0 mg, 0.333 mmol) in dichloromethane (2.2 mL) at room temperature. The originally colorless solution turned slightly brown upon the addition of DBU and the color intensified over the course of the reaction. After 2 h, the mixture was concentrated under reduced pressure and the residue was immediately purified by flash

chromatography (hexanes/EtOAc, 4:1 + 0.2 vol-% of triethylamine). The title compound was obtained as a colorless syrup (75.1 mg, 60% yield; *α*/*β* ≈ 1:12 mixture). **$[\alpha]_D^{20}$ =** +12.2 (*c* 0.97, $CHCl_3$). **^{1}H NMR (400 MHz, $CDCl_3$,** signals of the *β*-anomer only): δ 8.64 (s, 1H), 6.04 (d, *J* = 8.1 Hz, 1H), 4.53 (dd, *J* = 9.8, 2.6 Hz, 1H), 4.14 (dq, *J* = 9.7, 6.3 Hz, 1H), 4.01 (t, *J* = 2.7 Hz, 1H), 3.56 (s, 3H), 3.54 (s, 3H), 3.37 (dd, *J* = 8.2, 2.8 Hz, 1H), 2.13 (s, 3H), 1.22 (d, *J* = 6.3 Hz, 3H). **^{13}C{^{1}H} NMR (101 MHz, $CDCl_3$):** δ 170.4, 161.4, 96.6, 91.2 (br), 80.2, 77.2, 74.4, 68.8, 61.6, 59.5, 21.1, 17.5. **IR (film):** 3339, 2983, 2936, 2835, 1732, 1673, 1451, 1373, 1294, 1232, 1199, 1172, 1140, 1099, 1045, 1001, 966, 906, 836, 795, 734, 713, 645, 603, 574, 507, 475, 432 cm^{-1}. **HRMS-ESI *m/z*:** $[M+Na]^+$ calcd for $C_{12}H_{18}NO_6Cl_3Na$ 400.0092; found 400.0092.

Fragment Coupling and Completion of the Total Synthesis of Aldgamycin N

Protected Macrolactone (S5). In a 10-mL Schlenk tube, triethylsilyl trifluoromethanesulfonate (0.10 mL, 0.44 mmol) was added to a solution of compound **15** (222 mg, 0.313 mmol) and 2,6-lutidine (73 µL, 0.63 mmol) in dichloromethane (3.1 mL) at –25 °C. After 1 h, the reaction was quenched with saturated aqueous sodium bicarbonate solution (2 mL) at –25 °C. The mixture was warmed to room temperature, diluted with *tert*-butyl methyl ether (20 mL) and washed with saturated aqueous sodium bicarbonate solution (15 mL). The aqueous layer was extracted with *tert*-butyl methyl ether (4 × 10 mL) and the combined organic layers were dried over anhydrous sodium sulfate. The drying agent was filtered off and the solvent was removed under reduced pressure. Purification of the residue by flash chromatography (hexanes/EtOAc, 15:1) furnished the title compound as a colorless gum (235 mg, 91% yield) **$[\alpha]_D^{20}$ =** +32.8 (*c* 1.0, $CHCl_3$). **^{1}H NMR (400 MHz, $CDCl_3$):** δ 7.67 – 7.60 (m, 4H), 7.45 – 7.33 (m, 6H), 7.30 – 7.26 (m, 2H), 6.90 – 6.85 (m, 2H), 6.72 (dd, *J* = 15.7, 9.5 Hz, 1H), 5.79 – 5.65 (m, 1H), 5.74 (dd, *J* = 15.7, 0.8 Hz, 1H), 5.49 – 5.39 (m, 1H), 5.22 (quint, *J* = 6.2 Hz, 1H), 4.61 (d, *J* = 10.6 Hz, 1H), 4.44 (d, *J* = 10.6 Hz, 1H), 3.80 (s, 3H), 3.67 (dd, *J* = 10.2, 6.6 Hz, 1H), 3.59 (dd, *J* = 10.2, 5.5 Hz, 1H), 3.10 (dd, *J* = 9.7, 2.1 Hz, 1H), 2.97 – 2.79 (m, 2H), 2.56 (tq, *J* = 9.6, 6.7 Hz, 1H), 2.37 – 2.14 (m, 2H), 1.77 (br d, *J* = 14.1 Hz, 1H), 1.54 (dd, *J* = 14.2, 9.1 Hz, 1H), 1.44 (s, 3H), 1.23 (d, *J* = 6.3 Hz, 3H), 1.13 (d, *J* = 6.6 Hz, 3H), 1.11 (d, *J* = 6.1 Hz, 3H), 1.04 (s, 9H), 0.94 (t, *J* = 7.9 Hz, 9H), 0.72 – 0.56 (m, 6H). **^{13}C{^{1}H} NMR (101 MHz, $CDCl_3$):** δ 166.1, 159.3, 150.3, 135.76, 135.74, 133.7, 133.6, 131.2, 129.81, 129.78, 129.4, 128.0, 127.8, 121.5, 113.9, 89.1, 89.0, 81.2, 69.8, 69.5, 64.3, 55.5, 51.5, 40.5, 27.0, 22.3, 19.4, 18.8, 18.5, 7.28, 6.37. **IR (film):** 3071, 3045, 2956, 2932, 2874, 1816, 1717, 1655, 1613, 1588, 1514, 1461, 1428, 1370, 1302, 1249, 1199, 1163, 1111, 1081, 1040, 1001, 986, 823, 785, 741,

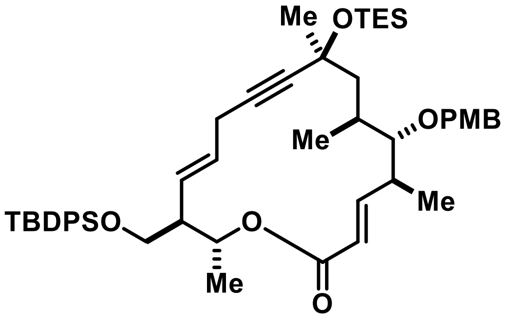

703, 613, 505, 488, 431 cm^{-1}. **HRMS-ESI *m/z*:** $[M+Na]^+$ calcd for $C_{50}H_{70}O_6Si_2Na$ 845.4603; found 845.4608.

Glycosyl Acceptor 16. In a 10-mL round-bottomed flask, DDQ (129 mg, 0.568 mmol) was added in one portion to a vigorously stirred solution of compound **S5** (235 mg, 0.286 mmol) in dichloromethane (4.0 mL) and water (1.0 mL). After stirring for 1 h, the reaction mixture, which had turned from very dark green to brown, was diluted with *tert*-butyl methyl ether (20 mL) and washed with saturated aqueous sodium bicarbonate solution (20 mL, then 10 mL). The combined aqueous phases were extracted with *tert*-butyl methyl ether (4 × 10 mL) and the organic layers were dried over anhydrous sodium sulfate. The drying agent was filtered off and the solvent was removed under reduced pressure. Purification of the crude material by flash chromatography (hexanes/EtOAc, 15:1 → 9:1) furnished the title compound as a colorless gum (173 mg, 86% yield). $[\alpha]_D^{20}$ = +45.0 (*c* 0.78, $CHCl_3$). **^{1}H NMR (400 MHz, $CDCl_3$):** δ 7.67 – 7.59 (m, 4H), 7.46 – 7.34 (m, 6H), 6.77 (dd, *J* = 15.7, 9.3 Hz, 1H), 5.76 (dd, *J* = 15.7 Hz, 0.8 Hz, 1H), 5.57 (dd, *J* = 15.4, 9.2 Hz, 1H), 5.38 (ddd, *J* = 15.3, 8.0, 4.1 Hz, 1H), 5.23 (dq, *J* = 8.0, 6.3 Hz, 1H), 3.68 – 3.60 (m, 2H), 3.30 – 3.23 (m, 1H), 2.97 – 2.78 (m, 2H), 2.53 – 2.41 (m, 1H), 2.24 (tt, *J* = 9.4, 5.2 Hz, 1H), 2.11 – 2.00 (m, 1H), 1.59 (dd, *J* = 13.8, 2.7 Hz, 1H), 1.51 – 1.43 (m, 4H), 1.21 (d, *J* = 6.3 Hz, 3H), 1.15 (d, *J* = 6.5 Hz, 3H), 1.11 (d, *J* = 6.9 Hz, 3H), 1.05 (s, 9H), 0.94 (t, *J* = 7.9 Hz, 9H), 0.71 – 0.55 (m, 6H). **$^{13}C\{^1H\}$ NMR (101 MHz, $CDCl_3$):** δ 166.0, 150.2, 135.8, 133.62, 133.56, 129.85, 129.81, 129.76, 127.89, 127.83, 127.80, 121.5, 81.9, 81.5, 69.9, 69.4, 64.3, 52.0, 40.6, 34.4, 27.0, 22.6, 19.4, 18.9, 18.9, 18.0, 7.24, 6.36. **IR (film):** 3473, 3070, 3050, 2956, 2932, 2875, 1717, 1655, 1460, 1428, 1362, 1271, 1231, 1163, 1112, 1062, 1005, 985, 878, 824, 740, 703, 606, 505, 435, 420 cm^{-1}. **HRMS-ESI *m/z*:** $[M+Na]^+$ calcd for $C_{42}H_{62}O_5Si_2Na$ 725.4028; found 725.4034.

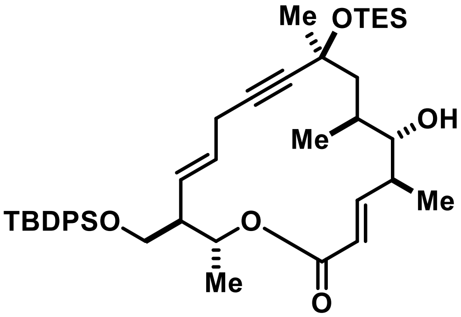

Propargylic Alcohol 20. In a 25-mL Schlenk tube, a solution of triethylsilyl trifluoromethanesulfonate in dichloromethane (0.088 M, 0.29 mL, 0.026 mmol) was added to a solution of alcohol **16** (121 mg, 0.172 mmol) and trichloroacetimidate **13g** (91.4 mg, 0.226 mmol) in dichloromethane (6.9 mL) at −78 °C. The resulting mixture was stirred for 5 h at −78 °C before the reaction was quenched by the addition of triethylamine (0.10 mL) at this temperature. Saturated aqueous sodium bicarbonate solution (5 mL) and ethyl acetate (5 mL) were added and the mixture was

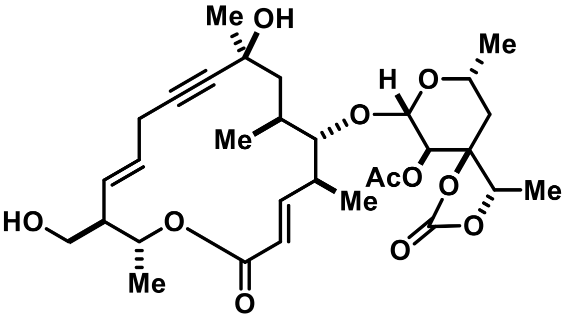

warmed to room temperature while stirring. It was washed with saturated aqueous sodium bicarbonate solution (15 mL) and the aqueous phase was extracted with ethyl acetate (5 × 10 mL). The combined organic layers were dried over anhydrous sodium sulfate, the drying agent was filtered off, and the solvent was removed under reduced pressure to give a slightly yellowish oil (256 mg).

In a 25-mL Schlenk tube, TASF (0.51 M solution in DMF, 2.1 mL, 1.1 mmol) was added to a solution of this material in anhydrous DMF (5.8 mL) at 0°C. After stirring for 1 h at this temperature, water (35 µL, 1.9 mmol) was added and stirring continued at room temperature for 15 h. The mixture was then diluted with ethyl acetate (30 mL) and washed with half-saturated aqueous sodium bicarbonate solution (50 mL) and brine (2 × 10 mL). The combined aqueous phases were extracted with ethyl acetate (5 × 15 mL) and the organic layers were dried over anhydrous sodium sulfate. The drying agent was filtered off and the filtrate was concentrated under reduced pressure. Remaining DMF was removed by co-evaporation with toluene (4 × 3 mL, 38 °C, 20 mbar) to leave a thick, amber gum. Purification of this crude material by flash chromatography (hexanes/EtOAc, 1:1 → 2:3) furnished the title compound as a thick, colorless gum (53.5 mg, 53% yield). $[\alpha]_D^{20}$ = +14.3 (*c* 1.6, $CHCl_3$). **^{1}H NMR (400 MHz, $CDCl_3$):** δ 6.93 (dd, *J* = 15.8, 7.3 Hz, 1H), 5.77 (dd, *J* = 15.8, 1.4 Hz, 1H), 5.72 – 5.49 (m, 2H), 5.18 (qd, *J* = 6.4, 4.7 Hz, 1H), 4.95 (d, *J* = 7.8 Hz, 1H), 4.70 (d, *J* = 7.8 Hz, 1H), 4.38 (q, *J* = 6.7 Hz, 1H), 3.92 (dqd, *J* = 12.4, 6.1, 2.0 Hz, 1H), 3.55 – 3.42 (m, 2H), 3.38 – 3.27 (m, 1H), 3.06 – 2.89 (m, 2H), 2.62 – 2.48 (m, 1H), 2.37 (qd, *J* = 7.2, 4.6 Hz, 1H), 2.23 – 2.10 (m, 1H), 2.08 (s, 3H), 1.96 – 1.85 (m, 1H), 1.89 (dd, *J* = 14.3, 2.2 Hz, 1H), 1.63 (dd, *J* = 14.3, 11.3 Hz, 1H), 1.55 – 1.48 (m, 1H), 1.48 (m, 3H), 1.37 (d, *J* = 6.8 Hz, 3H), 1.28 (d, *J* = 6.4 Hz, 3H), 1.26 (d, *J* = 6.2 Hz, 3H), 1.12 (d, *J* = 6.7 Hz, 3H), 1.07 (d, *J* = 7.0 Hz, 3H). **^{13}C{^{1}H} NMR (101 MHz, $CDCl_3$):** δ 168.9, 166.2, 153.7, 150.0, 129.1, 127.7, 121.2, 99.9 (br), 87.8, 85.5, 81.6 (br), 81.4, 81.1 (br), 70.3, 69.0, 68.75 (br), 68.66, 66.9, 63.0, 62.8, 51.3, 41.3, 39.6, 34.3, 22.3, 21.1, 20.6, 18.9, 18.0 (br), 16.6, 13.3. **IR (film):** 3481, 2976, 2933, 2881, 1804, 1754, 1707, 1656, 1452, 1378, 1328, 1278, 1229, 1197, 1157, 1116, 1087, 1069, 1051, 1010, 982, 914, 865, 826, 754, 687, 666, 618, 554, 513 cm^{-1}. **HRMS-ESI *m/z*:** $[M+Na]^+$ calcd for $C_{31}H_{44}O_{11}Na$ 615.2776; found 615.2775.

Alkenyl Stannane 26. In a 10-mL Schlenk tube, a solution of $[Cp^*RuCl]_4$ in dichloromethane (30 mg/mL, 0.43 mL, 12 µmol) was added to propargylic alcohol **20** (69.8 mg, 118 µmol) at room temperature, which caused the very dark green color of the catalyst solution to turn dark brown. A solution of tributyltin hydride in dichloromethane (0.28 M, 0.47 mL, 0.13 mmol) was then added over 45 min at room temperature. After complete addition, the dark red/brown

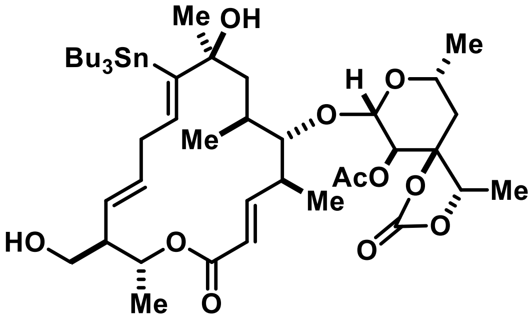

solution was stirred for further 10 min and then loaded directly onto silica gel (packed with hexanes/EtOAc, 4:1), eluting with hexanes (50 mL) and hexanes/EtOAc (4:1 → 3:1 → 2:1) to give the title compound as a slightly beige, amorphous solid (64.8 mg, 62% yield). **mp** = 140 – 143 °C. $[\alpha]_D^{20}$ = −46.9 (*c* 1.6, $CHCl_3$). **^{1}H NMR (400 MHz, $CDCl_3$):** δ 6.94 (dd, *J* = 15.9, 5.8 Hz, 1H), 5.89 (dd, *J* = 8.9, 5.8 Hz, 1H), 5.78 (dd, *J* = 15.9, 1.8 Hz, 1H), 5.78 – 5.69 (m, 1H), 5.37 – 5.27 (m, 1H), 5.25 (qd, *J* = 6.4, 2.3 Hz, 1H), 4.99 (d, *J* = 7.8 Hz, 1H), 4.72 (d, *J* = 7.8 Hz, 1H), 4.39 (q, *J* = 6.8 Hz, 1H), 3.95 (dqd, *J* = 12.5, 6.1, 2.0 Hz, 1H), 3.42 – 3.33 (m, 1H), 3.33 – 3.24 (m, 1H), 3.19 (dd, *J* = 9.0, 2.8 Hz, 1H), 2.81 – 2.71 (m, 1H), 2.71 – 2.60 (m, 2H), 2.60 – 2.50 (m, 1H), 2.36 (tdd. *J* = 9.3, 6.6, 2.4 Hz, 1H), 2.11 (s, 3H), 2.10 – 2.01 (m, 1H), 1.92 (dd, *J* = 14.4, 2.1 Hz, 1H), 1.80 – 1.71 (m, 1H), 1.67 (dd, *J* = 14.4, 11.2 Hz, 1H), 1.49 – 1.40 (m, 6H), 1.40 – 1.34 (m, 5H), 1.33 – 1.22 (m, 20H), 1.10 (d, *J* = 6.6 Hz, 3H), 1.06 (d, *J* = 7.2 Hz, 3H), 0.87 (t, *J* = 7.3 Hz, 9H). **^{13}C{^{1}H} NMR (101 MHz, $CDCl_3$):** δ 169.0, 166.4, 153.5, 149.1, 134.41 (br), 134.36, 126.2, 121.1, 100.9 (br), 88.5, 85.2, 81.3, 78.4, 70.2, 68.9, 67.3, 63.1, 51.4, 41.3, 38.5, 35.2 (br), 34.6 (br), 29.4, 28.0, 27.7, 21.1, 20.5, 19.0, 17.1, 15.7, 13.9, 13.3, 12.9. **^{119}Sn{^{1}H} NMR (149 MHz, $CDCl_3$):** δ −58.7. **IR (film):** 3513, 2956, 2924, 2872, 2853, 1810, 1757, 1703, 1638, 1458, 1416, 1376, 1331, 1279, 1228, 1196, 1158, 1145, 1117, 1087, 1069, 1052, 1007, 983, 939, 914, 866, 771, 757, 688, 666, 628, 596, 533, 504 cm^{-1}. **HRMS-ESI *m/z*:** $[M+Na]^+$ calcd for $C_{43}H_{72}O_{11}SnNa$ 907.3989; found 907.3997.

Hydroxy Ketone 28. In a 10-mL Schlenk tube, 2,6-di-*tert*-butylpyridine (39 µL, 0.17 mmol) and copper(II) trifluoroacetate monohydrate (43.0 mg, 0.14 mmol) were added to a solution of alkenyl stannane **26** (64.4 mg, 72.9 µmol) in anhydrous DMSO (0.97 mL, previously degassed with a stream of argon) at room temperature to give a green, homogeneous solution. The flask was immersed into a preheated oil bath at 48 °C and the mixture stirred at this temperature for 50 min. During this time, the color of the originally green solution slowly faded to become turbid yellow; eventually, a fine, amber suspension was obtained. At this point, the mixture was diluted with ethyl acetate (10 mL) and washed with saturated aqueous ammonium chloride solution (2 × 10 mL). The combined aqueous phases were extracted with ethyl acetate (5 × 10 mL) and the organic layers dried over anhydrous sodium sulfate. The drying agent was filtered off and the filtrate was concentrated under reduced pressure. Most of the remaining DMSO was removed by co-evaporation with toluene (2 × 3 mL, 38 °C, 20 mbar), followed by drying under high vacuum (10^{-3} mbar, 1 h) to leave a thick, amber gum. The residue was purified by flash chromatography (hexanes/EtOAc, 3:2 → 1:2) to give allene **S6** (8.9 mg, 21% yield) and hydroxy ketone **28**, still containing small amounts of stannoxane byproducts and DMSO. A second chromatographic purification of the latter sample with dichloromethane/methanol (60:1) as eluent removed

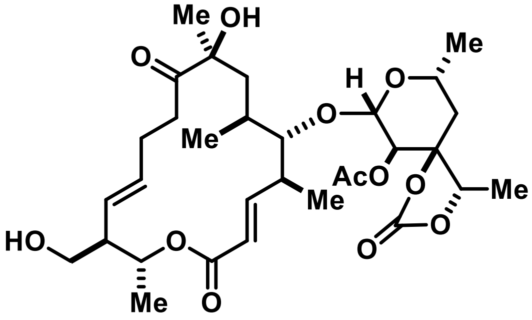

these impurities and furnished the title compound as a colorless foam (27.3 mg, 61% yield). $[\alpha]_D^{20}$ = −21.1 (*c* 0.37, $CHCl_3$). **^{1}H NMR (400 MHz, $CDCl_3$):** δ 6.77 (dd, *J* = 15.8, 7.9 Hz, 1H), 5.75 (dd, *J* = 15.8, 1.2 Hz, 1H), 5.53 (ddd, *J* = 15.4, 8.8, 4.5 Hz, 1H), 5.29 (dd, *J* = 15.5, 9.3 Hz, 1H), 5.10 (dq, *J* = 8.0, 6.3 Hz, 1H), 4.94 (d, *J* = 7.7 Hz, 1H), 4.67 (d, *J* = 7.7 Hz, 1H), 4.39 (q, *J* = 6.8 Hz, 1H), 3.91 (dqd, *J* = 12.1, 5.9, 2.0 Hz, 1H), 3.70 (s, 1H), 3.60 – 3.42 (m, 2H), 3.27 (d, *J* = 8.1, 2.5 Hz, 1H), 2.72 – 2.48 (m, 2H), 2.45 – 2.25 (m, 3H), 2.25 – 2.15 (m, 1H), 2.09 (s, 3H), 1.90 (dd, *J* = 14.3, 2.2 Hz, 1H), 1.88 – 1.75 (m, 2H), 1.72 – 1.65 (m, 1H), 1.63 (dd, *J* = 14.3, 11.2 Hz, 1H), 1.39 – 1.34 (m, 1H), 1.37 (d, *J* = 6.8 Hz, 3H), 1.31 (s, 3H), 1.28 (d, *J* = 6.4 Hz, 3H), 1.25 (d, *J* = 6.2 Hz, 3H), 1.10 (d, *J* = 6.8 Hz, 3H), 0.99 (d, *J* = 6.6 Hz, 3H). **^{13}C{^{1}H} NMR (101 MHz, $CDCl_3$):** δ 212.9, 168.9, 165.7, 153.7, 150.0, 133.6, 128.6 (br), 121.3, 99.4 (br), 87.5, 85.5, 81.3, 79.0, 70.4, 69.2, 66.9, 62.8, 52.6, 41.3, 40.2, 38.2 (br), 35.9, 33.3, 28.2, 26.1, 21.1, 20.5, 18.96, 18.88 (br), 17.2, 13.3. **IR (film):** 3488, 2971, 2932, 2881, 1807, 1754, 1707, 1652, 1597, 1456, 1379, 1328, 1281, 1229, 1196, 1157, 1143, 1115, 1088, 1070, 1052, 1008, 982, 957, 914, 868, 827, 755, 687, 667, 616, 561, 547 cm^{-1}. **HRMS-ESI *m/z*:** $[M+Na]^+$ calcd for $C_{31}H_{46}O_{12}Na$ 633.2881; found 633.2880.

Analytical Data of the allene side product (**S6**): $[\alpha]_D^{20}$ = +72.3 (*c* 0.57, $CHCl_3$). **^{1}H NMR (400 MHz, $CDCl_3$):** δ 6.78 (dd, *J* = 15.7, 8.7 Hz, 1H), 5.73 (dd, *J* = 15.7, 1.1 Hz, 1H), 5.64 (dt, *J* = 15.6, 5.7 Hz, 1H), 5.31 (ddt, *J* = 15.6, 9.0, 1.7 Hz, 1H), 5.14 – 5.03 (m, 2H), 4.95 (d, *J* = 7.8 Hz, 1H), 4.71 (d, *J* = 7.8 Hz, 1H), 4.38 (q, *J* = 6.8 Hz, 1H), 3.92 (dqd, *J* = 12.3, 6.2, 2.1 Hz, 1H), 3.60 – 3.43 (m, 2H), 3.29 (dd, *J* = 7.7, 3.1 Hz, 1H), 2.73 – 2.67 (m, 2H), 2.60 – 2.49 (m, 1H), 2.38 – 2.29 (m, 1H), 2.23 – 2.15 (m, 1H), 2.08 (s, 3H), 1.90 (dd, *J* = 14.3, 2.2 Hz, 1H), 1.84 – 1.74 (m, 2H), 1.66 (d, *J* = 2.9 Hz, 3H), 1.64 (dd, *J* = 14.3, 11.3 Hz, 1H), 1.56 – 1.46 (m, 1H), 1.37 (d, *J* = 6.8 Hz, 3H), 1.28 (d, *J* = 6.4 Hz, 3H), 1.25 (d, *J* = 6.2 Hz, 3H), 1.12 (d, *J* = 6.7 Hz, 3H), 0.88 (d, *J* = 6.7 Hz, 3H). **^{13}C{^{1}H} NMR (101 MHz, $CDCl_3$):** δ 201.3, 169.0, 165.9, 153.7, 149.9, 133.5, 127.5, 121.4, 100.1, 99.3 (br), 89.2 (br), 87.2, 85.6, 81.4, 70.5, 69.2, 66.9, 63.0, 51.9, 41.4, 40.8, 35.1 (br), 34.8, 32.2, 21.1, 20.6, 20.4 (br), 18.9, 17.4, 16.4 (br), 13.3. **IR (film):** 3500, 2973, 2930, 1809, 1756, 1712, 1654, 1457, 1378, 1281, 1229, 1196, 1157, 1143, 1088, 1070, 1052, 1010, 914, 772, 733, 687, 619 cm^{-1}. **HRMS-ESI *m/z*:** $[M+H]^+$ calcd for $C_{31}H_{45}O_{10}$ 577.3007; found 577.3009.

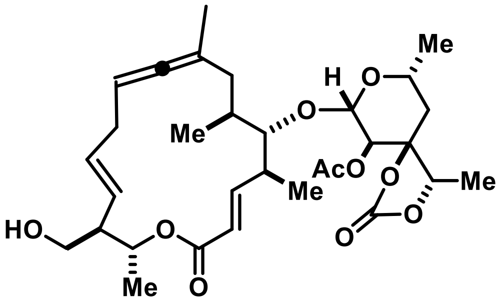

Glycosylated Macrolide 29. In a 10-mL Schlenk tube with cooling jacket, a solution of alcohol **28** (25.2 mg, 41.3 µmol) and trichloroacetimidate **25b** (34 mg, 90 mmol) in dichloromethane/acetonitrile (1:1, 1.40 mL) was cooled to −40 °C before a solution of triethylsilyl

trifluoromethanesulfonate in dichloromethane (0.088 M, 0.11 mL, 9.7 µmol) was added. After 1.5 h, a second batch of the same triethylsilyl trifluoromethane-sulfonate solution (0.10 mL, 8.8 µmol) was introduced and stirring was continued for another 4.5 h. The reaction was quenched by the addition of triethylamine (58 µL, 0.42 mmol) at –40 °C. Saturated aqueous sodium bicarbonate solution (1 mL) and ethyl acetate (3 mL) were added and the mixture was warmed to room temperature while stirring. It was washed with saturated aqueous sodium bicarbonate solution (10 mL) and the aqueous phase was extracted with ethyl acetate (5 × 10 mL). The combined organic layers were dried over anhydrous sodium sulfate, the drying agent was filtered off, and the solvent was removed under reduced pressure. Purification of the residue by flash chromatography (hexanes/acetone, 4:1 → 3:1) furnished the title compound as a colorless wax (17.1 mg, 50% yield; for analytical purposes, the sample was re-chromatographed with dichloromethane/methanol (90:1) to remove trace impurities (14.8 mg, 43% yield). A second fraction contained the corresponding α-anomer (9.1 mg, 27% yield).

Analytical and spectral data of the β–anomer **29a**: $[\alpha]_D^{20}$ = −9.7 (*c* 0.72, $CHCl_3$). **^{1}H NMR (400 MHz, $CDCl_3$):** δ 6.68 (dd, *J* = 15.6, 9.4 Hz, 1H), 5.75 (dd, *J* = 15.6, 0.9 Hz, 1H), 5.45 – 5.29 (m, 2H), 5.08 (dq, *J* = 9.2, 6.3 Hz, 1H), 4.94 (d, *J* = 7.7 Hz, 1H), 4.67 (d, *J* = 7.7 Hz, 1H), 4.61 (d, *J* = 8.0 Hz, 1H), 4.44 (dd, *J* = 9.9, 2.6 Hz, 1H), 4.38 (q, *J* = 6.8 Hz, 1H), 3.97 – 3.85 (m, 4H), 3.68 (br s, 1H), 3.53 – 3.51 (m, 6H), 3.46 (dd, *J* = 9.5, 6.3 Hz, 1H), 3.26 (br d, *J* = 9.2 Hz, 1H), 3.05 (dd, *J* = 8.0, 2.9 Hz, 1H), 2.70 – 2.48 (m, 2H), 2.40 – 2.13 (m, 4H), 2.12 – 2.09 (m, 6H), 1.89 (dd, *J* = 14.3, 2.2 Hz, 1H), 1.74 – 1.66 (m, 2H), 1.62 (dd, *J* = 14.4, 11.2 Hz, 1H), 1.56 – 1.43 (br m, 1H), 1.37 (d, *J* = 6.8 Hz, 3H), 1.32 (s, 3H), 1.29 (d, *J* = 6.3 Hz, 3H), 1.24 (d, *J* = 6.1 Hz, 3H), 1.16 (d, *J* = 6.3 Hz, 3H), 1.08 (d, *J* = 6.7 Hz, 3H), 0.99 (d, *J* = 6.8 Hz, 3H).

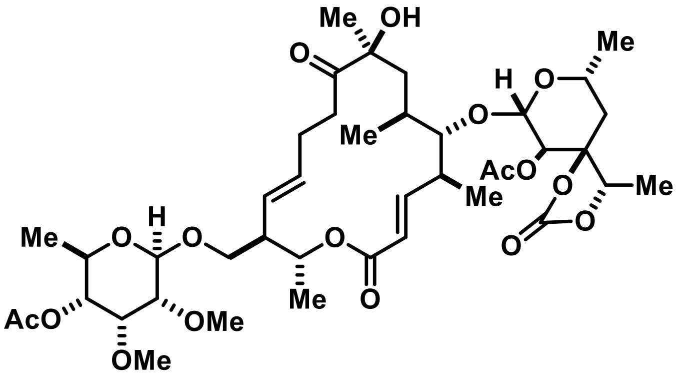

^{13}C{^{1}H} NMR (101 MHz, $CDCl_3$): δ 212.8, 170.3, 168.9, 165.5, 153.7, 150.1, 132.0, 129.9 (br), 121.7, 101.2, 99.7, 87.7, 85.6, 81.4, 80.7, 79.0, 78.0, 74.9, 70.5, 70.2, 70.0, 67.4, 66.8, 61.7, 59.8, 50.8 (br), 41.3, 40.8, 37.6 (br), 36.6 (br), 33.5, 28.3 (br), 26.1, 21.1 (2C), 20.5, 19.2 (br), 19.1, 18.1, 17.5, 13.3. **IR (film):** 3469, 2927, 2854, 1808, 1740, 1711, 1652, 1455, 1376, 1230, 1196, 1157, 1142, 1086, 1069, 1047, 1008, 963, 914, 868, 803, 754, 687, 666, 614, 549, 509 cm^{-1}. **HRMS-ESI *m/z*:** $[M+Na]^+$ calcd for $C_{41}H_{62}O_{17}Na$ 849.3879; found 849.3882.

Analytical and spectral data of the α-anomer **29b**: $[\alpha]_D^{20}$ = +20.5 (*c* 0.60, $CHCl_3$). **^{1}H NMR (400 MHz, $CDCl_3$):** δ 6.69 (dd, *J* = 15.6, 9.4 Hz, 1H), 5.76 (dd, *J* = 15.6, 0.8 Hz, 1H), 5.50 – 5.30 (m, 2H), 5.12 (dq,

J = 9.1, 6.2 Hz, 1H), 4.95 (d, J = 7.7 Hz, 1H), 4.83 (d, J = 4.1 Hz, 1H), 4.67 (d, J = 7.7 Hz, 1H), 4.47 (dd, J = 10.0, 2.7 Hz, 1H), 4.38 (q, J = 6.7 Hz, 1H), 4.20 (dq, J = 9.9, 6.3 Hz, 1H), 3.95 (t, J = 2.8 Hz, 1H), 3.95 – 3.85 (m, 1H), 3.77 – 3.66 (m, 2H), 3.52 (s, 3H), 3.48 (dd, J = 9.7, 5.3 Hz, 1H), 3.42 (s, 3H), 3.31 (dd, J = 4.1, 2.9 Hz, 1H), 3.25 (br d, J = 9.9 Hz, 1H), 2.72 – 2.61 (m, 1H), 2.61 – 2.49 (m, 1H), 2.48 – 2.38 (m, 1H), 2.37 – 2.14 (m, 3H), 2.12 (s, 3H), 2.11 (s, 3H), 1.89 (dd, J = 14.4, 2.1 Hz, 1H), 1.77 – 1.68 (m, 2H), 1.62 (dd, J = 14.3, 11.3 Hz, 1H), 1.54 – 1.44 (m, 1H), 1.38 (d, J = 6.7 Hz, 3H), 1.33 (s, 3H), 1.32 (d, J = 6.7 Hz, 3H), 1.25 (d, J = 6.2 Hz, 3H), 1.13 (d, J = 6.4 Hz, 3H), 1.09 (d, J = 6.7 Hz, 3H), 0.99 (d, J = 6.8 Hz, 3H). **$^{13}C\{^1H\}$ NMR (101 MHz, $CDCl_3$):** δ 213.0, 170.5, 168.9, 165.5, 153.7, 150.0, 131.9, 129.9 (br), 121.8, 99.8, 97.1, 87.8, 85.6, 81.4, 79.1, 78.8, 75.5, 74.5, 70.5, 70.3, 69.7, 66.8, 61.8, 61.0, 57.4, 50.5, 41.3, 40.9 (br), 37.4 (br), 36.7 (br), 33.7, 28.6 (br), 26.2 (br), 21.22, 21.12, 20.5, 19.1 (2C), 18.2, 17.1, 13.3. **IR (film):** 3420, 2973, 2930, 1809, 1754, 1740, 1712, 1651, 1597, 1456, 1377, 1327, 1280, 1231, 1197, 1179, 1157, 1108, 1088, 1070, 1050, 1010, 982, 914, 869, 831, 772, 755, 716, 687, 614, 546 cm^{-1}. **HRMS-ESI *m/z*:** $[M+Na]^+$ calcd for $C_{41}H_{62}O_{17}Na$ 849.3879; found 849.3876.

Aldgamycin N (1). Barium hydroxide octahydrate (31.0 mg, 98.3 µmol) was added in one portion to a solution of **29a** (11.3 mg, 13.7 µmol) in THF/water (1:1 v/v, 1.2 mL) and the resulting mixture was vigorously stirred at room temperature. Over the course of the reaction a fine white suspension was formed. After 2 h, the mixture was diluted with ethyl acetate (10 mL) and washed with saturated aqueous ammonium chloride solution (10 mL). The aqueous phase was extracted with ethyl acetate (5 × 10 mL) and the combined organic layers were dried over anhydrous sodium sulfate. The drying agent was filtered off and the solvent was removed under reduced pressure. Purification of the residue by flash chromatography (dichloromethane/methanol, 30:1 → 25:1) furnished the title compound as a colorless gum containing trace impurities (6.8 mg, 69% yield).

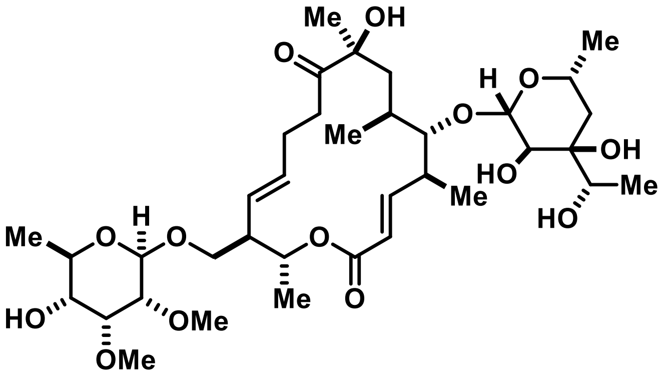

Analytically pure Aldgamycin N was obtained by preparative HPLC: Agilent 1260 Infinity pump, 150 mm length × 10 mm diameter YMC Triart C18 5 µm column, acetonitrile/water (40:60 v/v, 4.7 mL/min, 8.3 MPa, 298 K) eluent, UV-detection at 200 nm.

Analytical and spectral data of synthetic Aldgamycin N (**1**): $[\alpha]_D^{27}$ = −15.1 (*c* 0.13, $CHCl_3$) [lit.:[7] $[\alpha]_D^{27}$ = −13.2 (*c* 0.5, $CHCl_3$)]. **^1H NMR (600 MHz, $CDCl_3$):** δ 6.72 (dd, J = 15.5, 10.0 Hz, 1H, 3-H), 5.79 (d, J = 15.5 Hz, 1H, 2-H), 5.49 – 5.27 (m, 2H, 12-H, 13-H), 5.10 (dq, J = 8.9, 6.3 Hz, 1H, 15-H), 4.63 (d, J = 7.5 Hz, 1H, 1'-H), 4.56 (d, J = 7.7 Hz, 1H, 1"-H), 3.99 – 3.89 (m, 2H, 5'-H, 20-H[a]), 3.74 (t,

J = 3.1 Hz, 1H, 3”-H), 3.70 (br s, 1H, 8-OH), 3.68 – 3.60 (m, 2H, 2’-H, 7’-H), 3.61 (s, 3H, 8”-H_3), 3.53 (s, 3H, 7”-H_3), 3.53 – 3.48 (m, 1H, 5”-H), 3.45 (dd, J = 9.5, 6.4 Hz, 1H, 20-H^b), 3.36 (br d, J = 8.7 Hz, 1H, 5-H). 3.24 (d, J = 2.1 Hz, 1H, 3’-OH), 3.18 (ddd, J = 11.2, 9.3, 3.3 Hz, 1H, 4”-H), 3.04 (dd, J = 7.8, 2.9 Hz, 1H, 2”-H), 2.80 – 2.60 (m, 3H, 2’-OH, 10-H^a, 4-H), 2.45 (d, J = 8.5 Hz, 1H, 7’-OH), 2.39 – 2.32 (m, 1H, 14-H), 2.30 (d, J = 11.2 Hz, 1H, 4”-OH), 2.30 – 2.12 (m, 3H, 11-H^a, 10-H^b, 11-H^b), 1.86 – 1.71 (m, 2H, 7-H_2), 1.50 (dd, J = 13.6, 2.2 Hz, 1H, 4’-H^a), 1.40 – 1.31 (m, 1H, 4’-H^b), 1.33 (s, 3H, 19-H_3), 1.30 (d, J = 6.3 Hz, 3H, 16-H_3), 1.28 (d, J = 6.6 Hz, 3H, 8’-H_3), 1.25 (d, J = 6.3 Hz, 3H, 6”-H_3), 1.17 (d, J = 6.7 Hz, 3H, 17-H_3), 1.17 (d, J = 6.3 Hz, 3H, 6’-H_3), 1.00 (d, J = 6.9 Hz, 3H, 18-H_3). **$^{13}C\{^1H\}$ NMR (151 MHz, $CDCl_3$):** δ 212.7 (9-C), 165.5 (1-C), 150.8 (3-C), 132.0 (12-C), 129.6 (br, 13-C), 121.5 (2-C), 101.4 (1’-C), 101.0 (1”-C), 86.8 (5-C), 81.9 (2”-C), 79.9 (3”-C), 79.1 (8-C), 73.9 (7’-C), 73.8 (3’-C), 72.7 (4”-C), 72.5 (2’-C), 70.6 (5”-C), 69.9 (20-C), 69.7 (15-C), 66.8 (5’-C), 61.7 (8”-C), 59.8 (7”-C), 51.0 (br, 14-C), 41.1 (4-C), 39.4 (4’-C), 38.1 (br, 7-C), 36.7 (br, 10-C), 34.0 (6-C), 28.6 (br, 19-C), 25.9 (11-C), 20.7 (6’-C), 18.9 (16-C), 18.7 (17-C, 18-C), 18.2 (8’-C), 17.8 (6”-C). **IR (film):** 3469, 2973, 2931, 2884, 2838, 1705, 1651, 1454, 1378, 1355, 1323, 1279, 1234, 1162, 1081, 987, 963, 933, 893, 866, 806, 754, 667 cm^{-1}. **HRMS-ESI *m/z*:** $[M+Na]^+$ calcd for $C_{36}H_{60}O_{14}Na$ 739.3875; found 739.3885.

Isomer 30: This compound was formed as a side product (ca. 8% yield) in analogous reactions using K_2CO_3/MeOH instead of $Ba(OH)_2{\cdot}8H_2O$; it analyzed as follows:

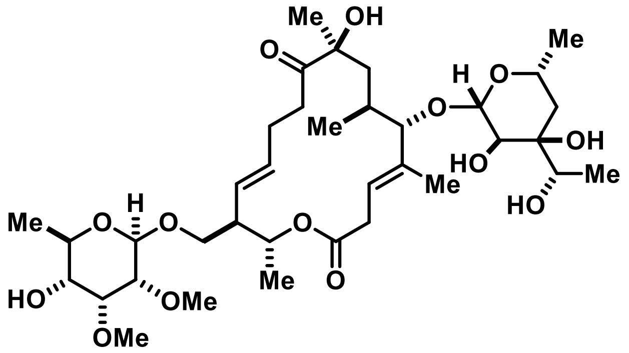

^1H NMR (600 MHz, $CDCl_3$): δ 5.64 – 5.55 (m, 2H), 5.28 (ddt, J = 15.7, 9.0, 1.6 Hz, 1H), 5.12 (qd, J = 6.2, 5.1 Hz, 1H), 4.56 (d, J = 7.7 Hz, 1H), 4.48 (d, J = 7.7 Hz, 1H), 4.20 (d, J = 5.5 Hz, 1H), 3.95 (dqd, J = 12.5, 6.2, 2.2 Hz, 1H), 3.83 (dd, J = 9.6, 7.1 Hz, 1H), 3.75 (t, J = 3.1 Hz, 1H), 3.67 – 3.60 (m, 3H), 3.62 (s, 3H), 3.55 – 3.48 (m, 1H), 3.52 (s, 3H), 3.41 (dd, J = 9.6, 6.4 Hz, 1H), 3.21 – 3.12 (m, 3H), 3.08 (ddd, J = 17.9, 5.5, 1.0 Hz, 1H), 2.88 (dt, J = 19.0, 6.4 Hz, 1H), 2.64 (dt, J = 19.0, 6.4 Hz, 1H), 2.42 – 2.26 (m, 6H), 1.93 (dd, J = 14.5, 3.2 Hz, 1H), 1.86 – 1.79 (m, 1H), 1.65 (br s, 3H), 1.61 (dd, J = 14.6, 7.5 Hz, 1H), 1.55 – 1.52 (m, 1H), 1.41 (ddd, J = 13.2, 11.2, 1.6 Hz, 1H), 1.33 (s, 3H), 1.29 (d, J = 6.6 Hz, 3H), 1.25 (d, J = 6.2 Hz, 3H), 1.22 (d, J = 6.2 Hz, 3H), 1.20 (d, J = 6.4 Hz, 3H), 0.99 (d, J = 7.0 Hz, 3H). **$^{13}C\{^1H\}$ NMR (151 MHz, $CDCl_3$):** δ 213.7, 170.6, 136.8, 132.1, 126.5, 121.5, 100.5, 98.3, 84.8, 81.9, 79.6, 79.4, 73.7, 73.5, 72.7, 72.0, 70.6, 70.3, 69.7, 67.1, 61.6, 59.6, 48.5, 40.2, 39.3, 35.9, 34.6, 34.2, 27.3, 24.9, 20.8, 18.6, 18.5, 18.2, 17.8, 14.4. **IR (film):** 3467, 2974, 2929, 1712, 1455, 1379, 1265, 1162, 1080, 1026, 961, 917, 732 cm^{-1}. **HRMS-ESI *m/z*:** $[M+Na]^+$ calcd for $C_{36}H_{60}O_{14}Na$ 739.3875; found 739.3876.

NMR Spectroscopic Comparison of Natural and Synthetic Aldgamycin N

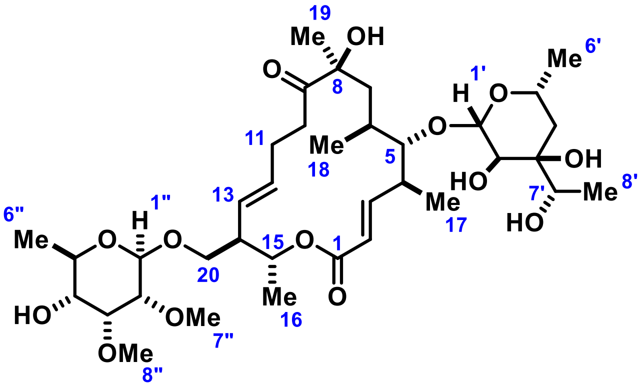

Table S1. Comparison of the NMR Data of Natural and Synthetic Aldgamycin N: Signals of the Aglycon (numbering as shown in the Insert; indiscernible signals reported without designating multiplicity)

Position	δ_C natural	δ_C synthetic	$\Delta\delta_C$	δ_H (*J*/Hz) natural	δ_H (*J*/Hz) synthetic	$\Delta\delta_H$
1	165.5	165.5	0.0	-	-	-
2	121.5	121.5	0.0	5.80 d (15.5)	5.79 d (15.5)	-0.01
3	150.7	150.8	+0.1	6.72 dd (15.5, 9.9)	6.72 dd (15.5, 10.0)	0.00
4	40.8	41.1	+0.3	2.61	2.67	+0.06
5	86.9	86.8	-0.1	3.38 br d (9.0)	3.36 br d (8.7)	-0.02
6	34.1	34.0	-0.1	1.35	* see below *	
7	38.0	38.1 (br)	+0.1	1.78	1.78	0.00
8	79.1	79.1	0.0	-	-	-
9	212.7	212.7	0.0	-	-	-
10	36.5	36.7 (br)	+0.2	2.69	2.69	0.00
				2.22	2.22	0.00
11	26.0	25.9	-0.1	2.27	2.27	0.00
				2.19	2.17	-0.02
12	132.0	132.0	0.0	5.41 ddd (15.2, 9.3, 3.0)	5.41	0.00
13	129.3	129.6 (br)	+0.3	5.36 dd (15.2, 9.3)	5.35 dd (14.7, 9.8)	-0.01
14	51.0	51.0 (br)	0.0	2.32	2.35	+0.03
15	69.8	69.7	-0.1	5.10 dq (9.0, 6.5)	5.10 dq (8.9, 6.3)	0.00
16	18.8	18.9	+0.1	1.29 d (6.5)	1.30 d (6.3)	+0.01
17	20.6	18.7	-1.9	1.18 d (6.4)	1.17 d (6.7)	-0.01
18	18.7	18.7	0.0	1.02 d (6.7)	1.00 d (6.9)	-0.02
19	28.3	28.6 (br)	+0.3	1.33 s	1.33	0.00
20	69.9	69.9	0.0	3.94 dd (9.4, 5.0)	3.95 dd (9.3, 4.7)	+0.01
				3.46 dd (9.4, 5.0)	3.45 dd (9.5, 6.4)	-0.01

Table S2. Comparison of the NMR data of Natural and Synthetic Aldgamycin N: Carbohydrate Signals (numbering as shown in the Insert; indiscernible signals reported without designating multiplicity)

Position	δ_C natural	δ_C synthetic	$\Delta\delta_C$	δ_H (*J*/Hz) natural	δ_H (*J*/Hz) synthetic	$\Delta\delta_H$
1’	101.4	101.4	0.0	4.64 d (7.6)	4.63 d (7.5 Hz)	-0.01
2’	72.7	72.5	-0.2	3.62	3.62	0.00
3’	73.9	73.8	-0.1	-	-	-
4’	39.5	39.4	-0.1	1.50 br d (13.0)	1.50 dd (13.6, 2.2)	0.00
				1.36 d	1.35	-0.01
5’	66.9	66.8	-0.1	3.95	3.95	0.00
6’	20.6	20.7	+0.1	1.18 d (6.4)	1.17 d (6.3)	-0.01
7’	73.8	73.9	+0.1	3.65	3.65	0.00
8’	18.2	18.2	0.0	1.26 d (6.2)	1.28 d (6.6)	+0.02
1”	101.0	101.0	0.0	4.56 d (7.9)	4.56 d (7.7)	0.00
2”	82.0	81.9	-0.1	3.04 dd (7.9, 2.9)	3.04 dd (7.8, 2.9)	0.00
3”	79.9	79.9	0.0	3.75 t (2.9)	3.74 t (3.1)	-0.01
4”	72.6	72.7	+0.1	3.18 dd (9.2, 2.9)	3.18 ddd (11.2, 9.3, 3.3)	0.00
5”	70.7	70.6	-0.1	3.50	3.50	0.00
6”	17.8	17.8	0.0	1.25 d (6.2)	1.25 d (6.3)	0.00
7”	59.8	59.8	0.0	3.53 s	3.53 s	0.00
8”	61.7	61.7	0.0	3.62 s	3.61 s	-0.01

For H-6 (lit. 1.35 ppm, reported as multiplet overlapping with another multiplet), an unambiguous ^{1}H NMR spectroscopic assignment was not possible because no correlation with H-5, H-7 or H-18 was found in COSY, and no correlation with C-7 or C-8 was found in HMBC either. Moreover, even though C-6 shows a sharp ^{13}C{^{1}H} resonance (34.0 ppm), no HSQC correlation was found. However, selective 1D-COSY and 1D-TOCSY experiments suggest for H-6 a very broad resonance at ~1.48 ppm (Figure S2). Noticeably, H-6 also appears as a very broad multiplet for both C-1” anomers at the stage of the still protected compound **29** (cf. copies of spectra and Figure S3).

The ^{13}C resonance for C-17 was assigned by HSQC measurement (Figure S4), indicating that C-17 is overlapping with C-18 rather than with C-6’ (however H-17 and H-6’ overlap in ^{1}H NMR spectrum).

The -OH ^{1}H resonances for the hydroxy groups at C-8, C-2', C-3', C-7' and C-4" varied for different samples. For C-7, C-10, C-13, C-14 and C-19, particularly broad $^{13}C\{^1H\}$ NMR signals were observed at 298 K (cf. copies of spectra). Measurements at 288 K and 308 K did not lead to any significant line sharpening.

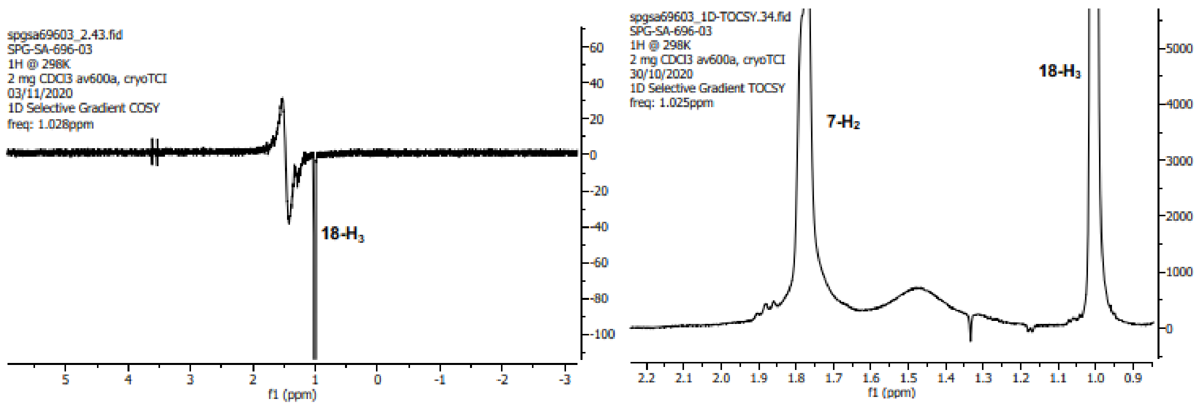

Fig. S2: Selective 1D-COSY (left) and 1D-TOCSY (right) measurement for the ^{1}H assignment of H-6 (excitation of 18-H_3), see text

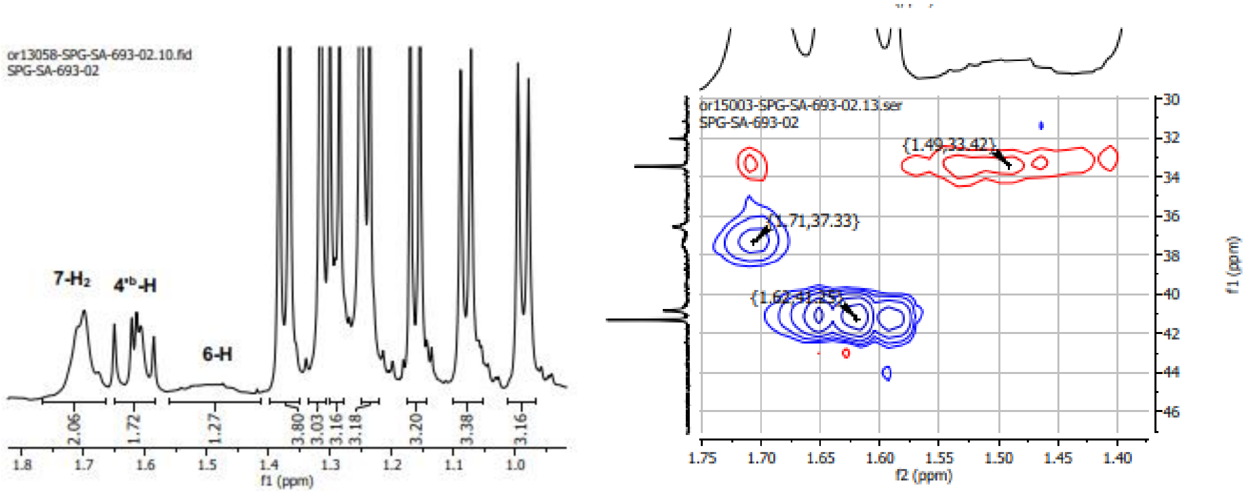

Fig. S3: H-6 exhibits a very broad ^{1}H resonance also at the stage of the protected compound **29** ($CDCl_3$, 6-H: δ_H 1.56 – 1.43 (m), 6-C: δ_C 33.4)

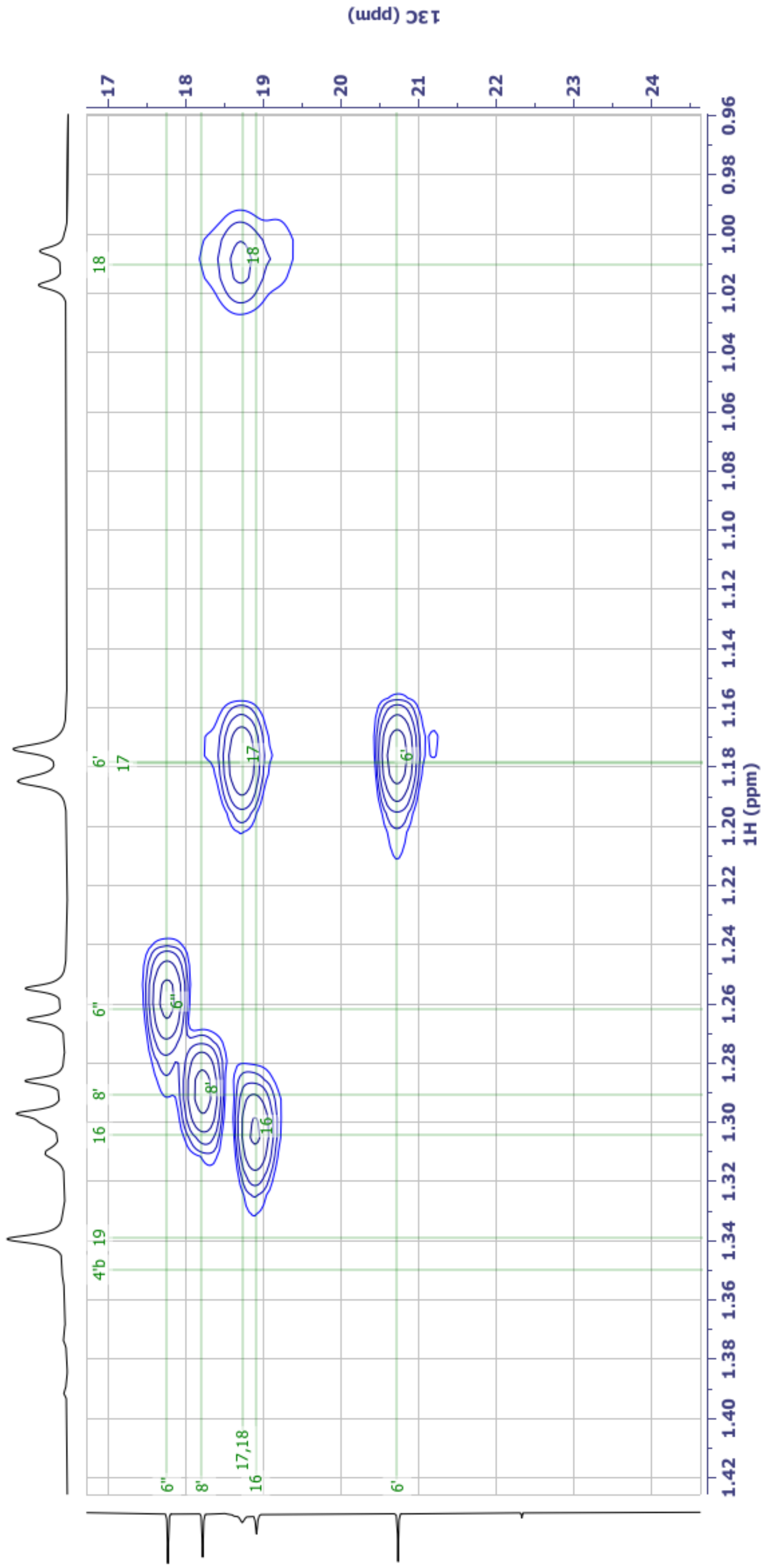

Fig. S4: Selective HSQC measurement for the ^{13}C assignment of C-17 and C-6'.

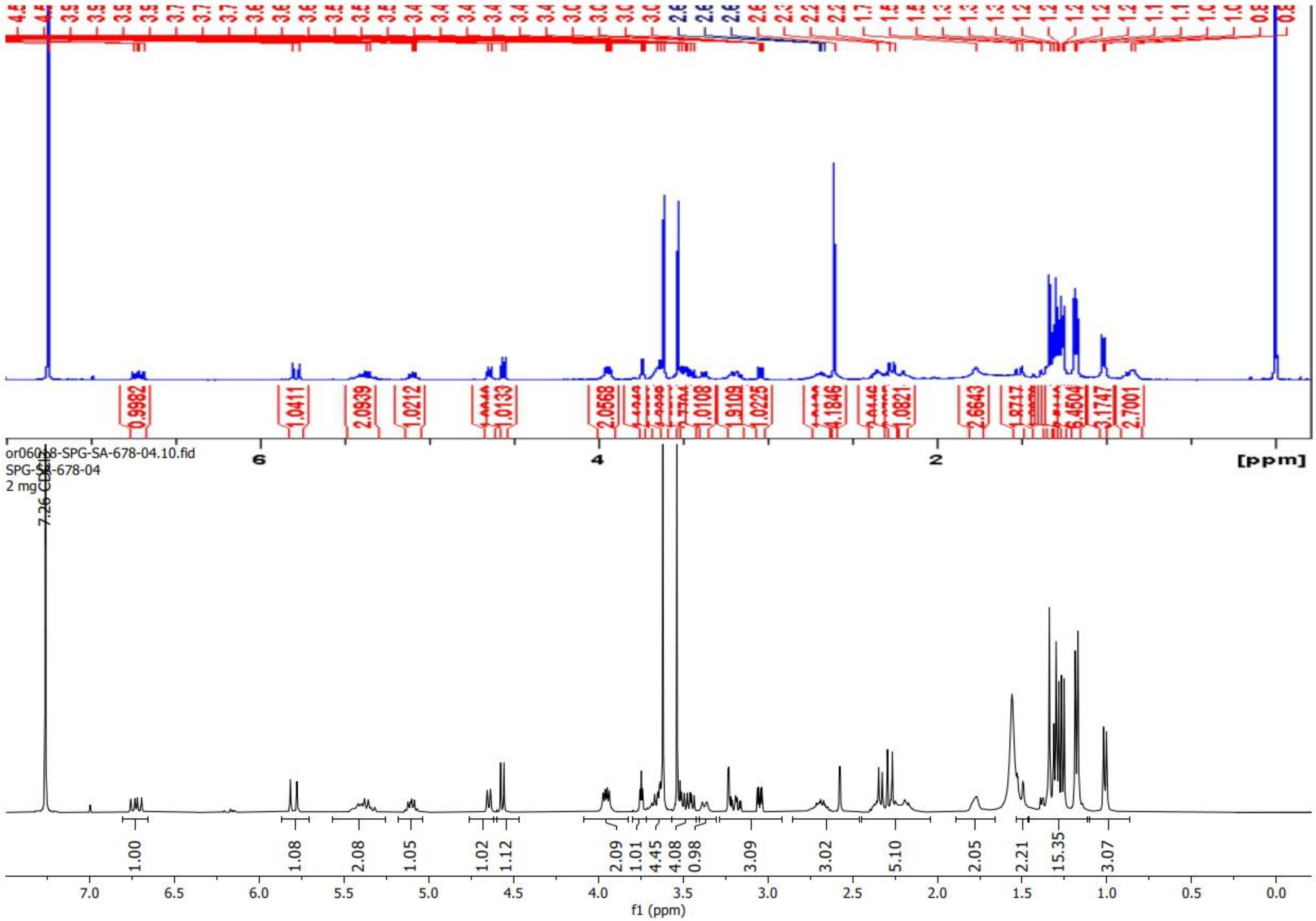

Fig. S5: Comparison of ^{1}H NMR spectra of natural (top, 400 MHz, $CDCl_3$) and synthetic Aldgamycin N (bottom, 400 MHz, $CDCl_3$).

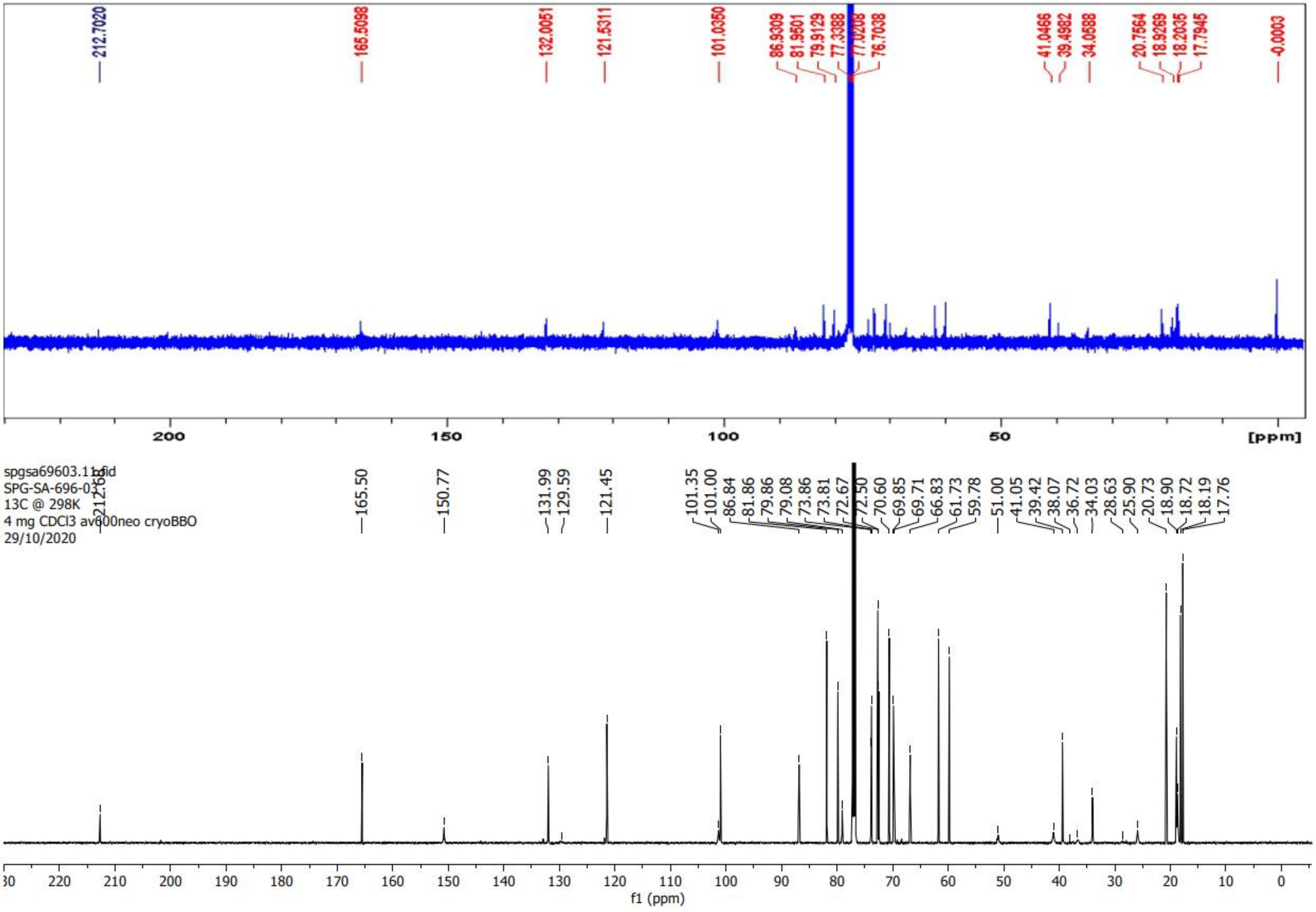

Fig. S6: Comparison of $^{13}C\{^1H\}$ NMR spectra of natural (top, 100 MHz, $CDCl_3$) and synthetic Aldgamycin N (bottom, 151 MHz, $CDCl_3$).

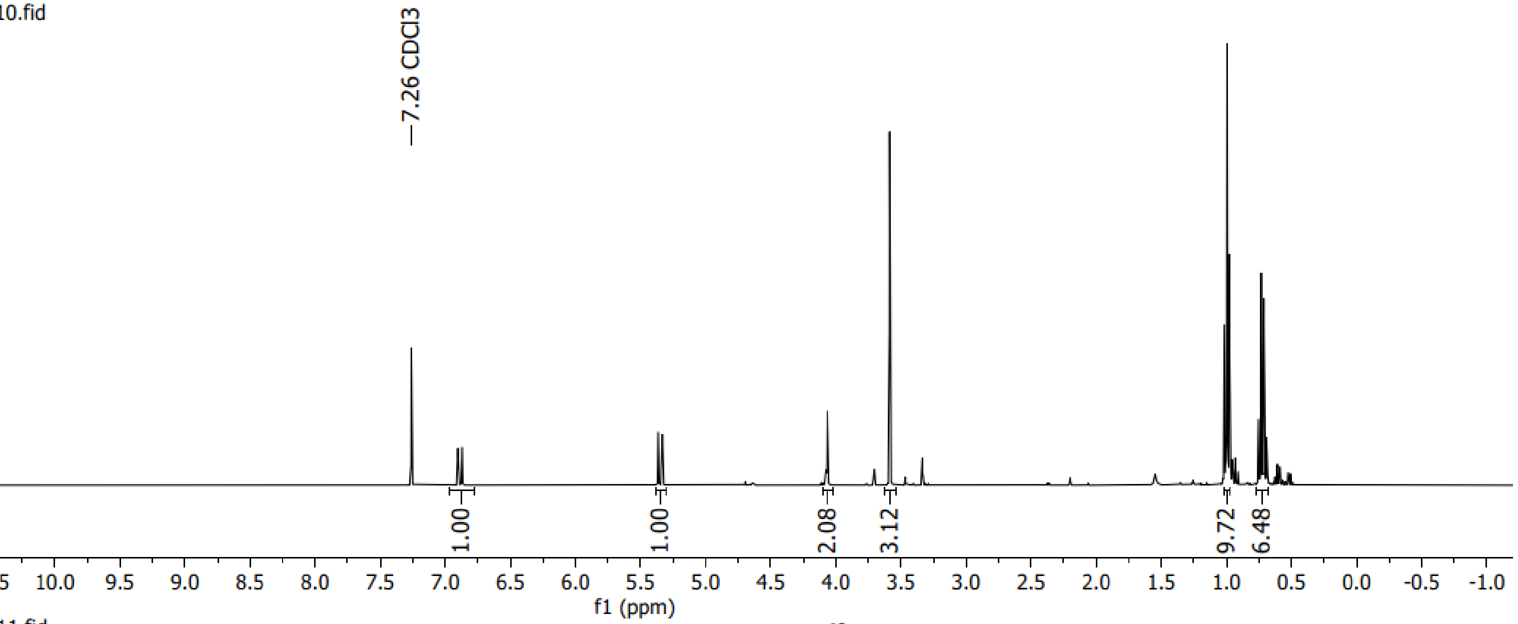

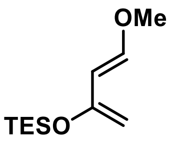

4 in $CDCl_3$

^1H (400 MHz)

$^{13}C\{^1H\}$ (101 MHz)

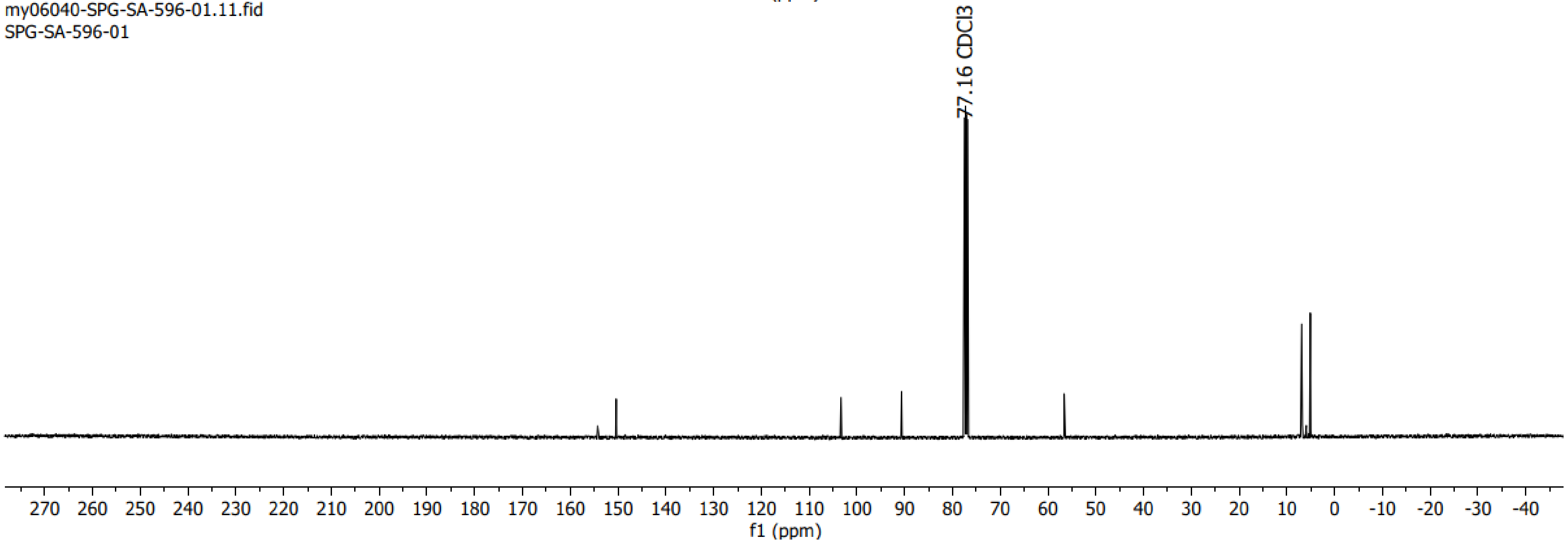

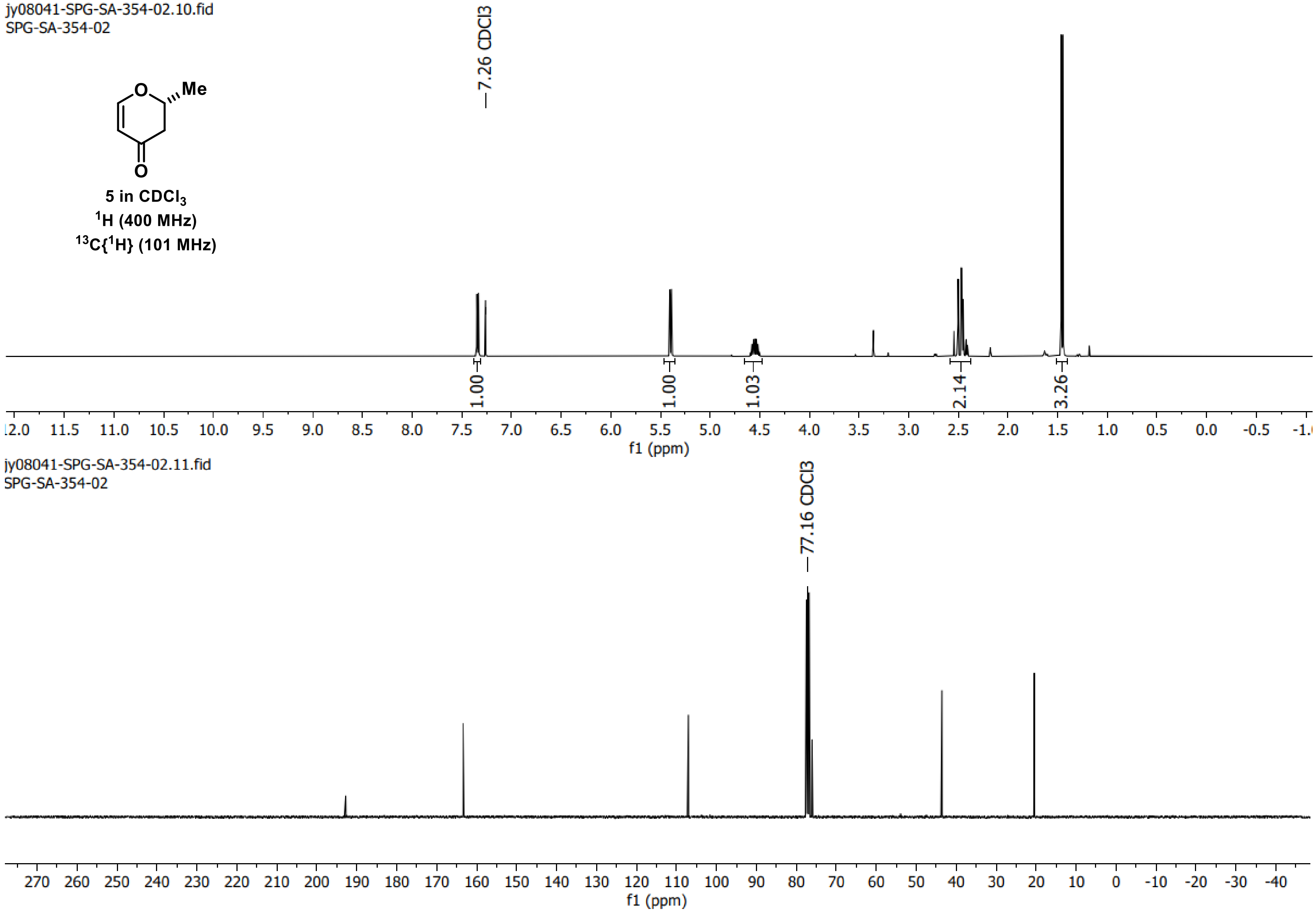

jy08041-SPG-SA-354-02.10.fid
SPG-SA-354-02
Me
O
O
5 in CDCl3
1H (400 MHz)
13C{1H} (101 MHz)
7.26 CDCl3
1.00
1.00
1.03
2.14
3.26
f1 (ppm)
jy08041-SPG-SA-354-02.11.fid
SPG-SA-354-02
77.16 CDCl3
f1 (ppm)

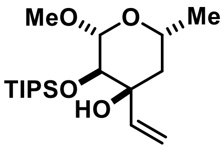

9 in $CDCl_3$
^{1}H (400 MHz)
^{13}C{^{1}H} (101 MHz)

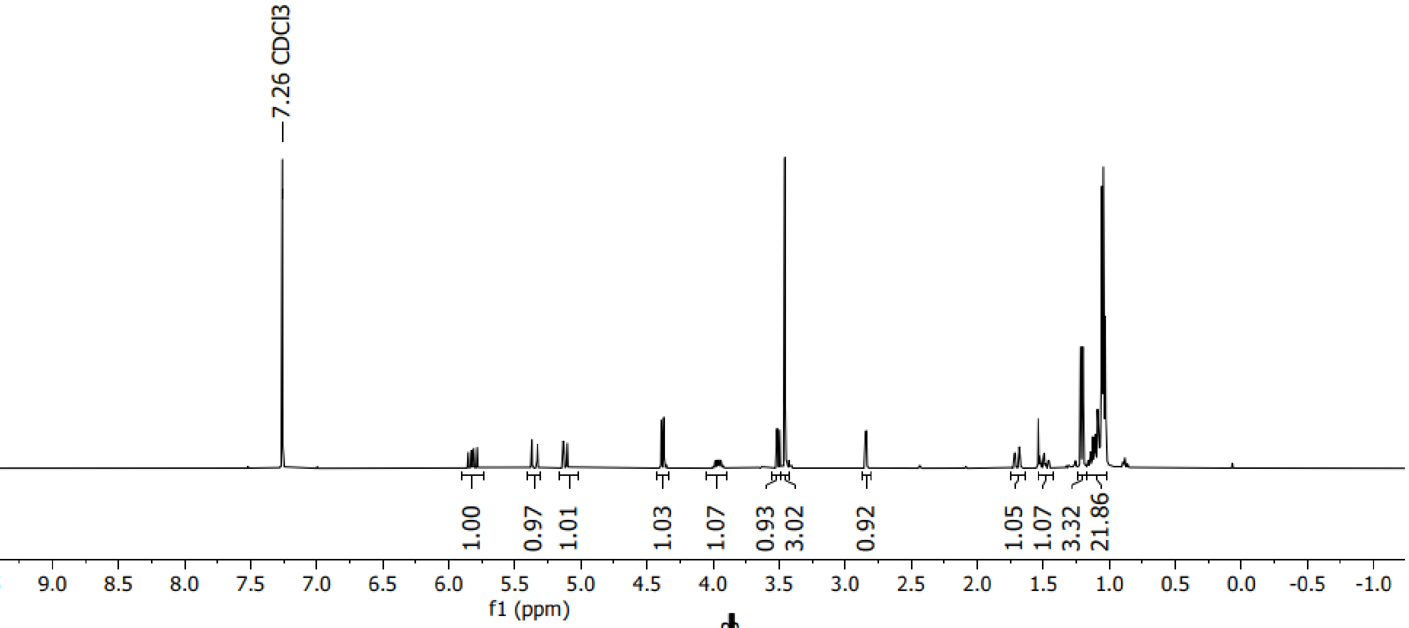

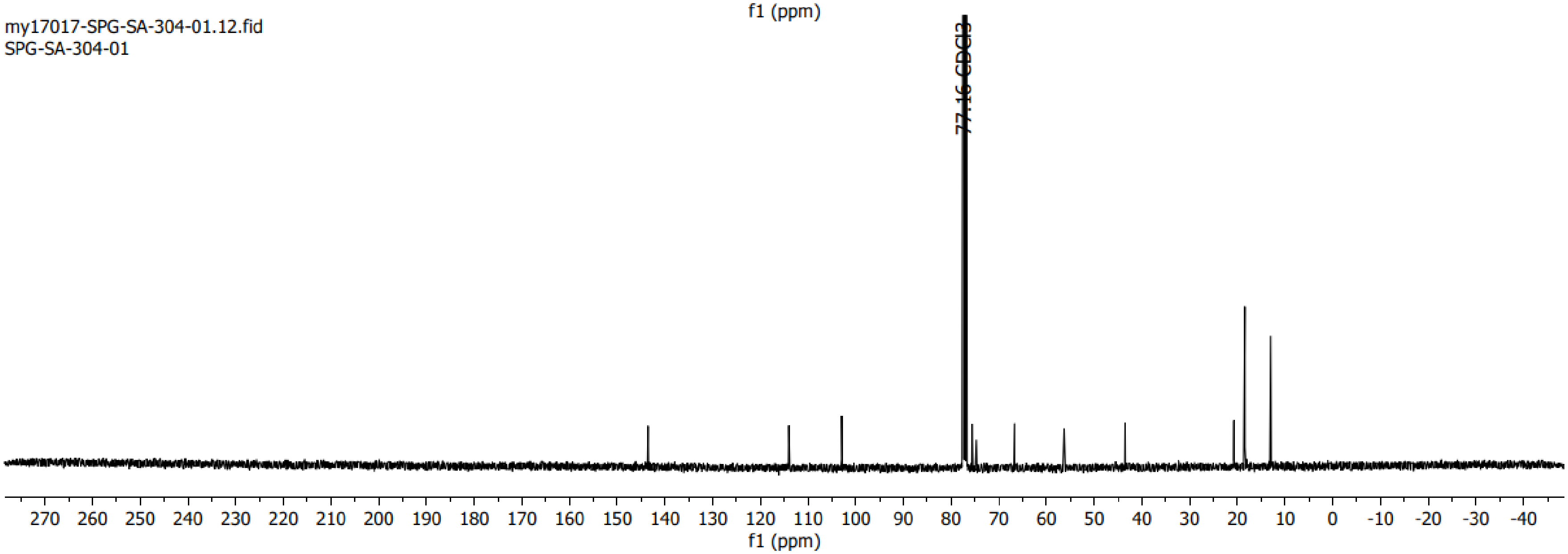

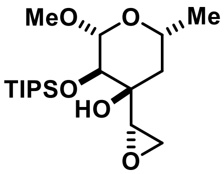

10 in $CDCl_3$
^1H (400 MHz)
$^{13}C\{^1H\}$ (101 MHz)

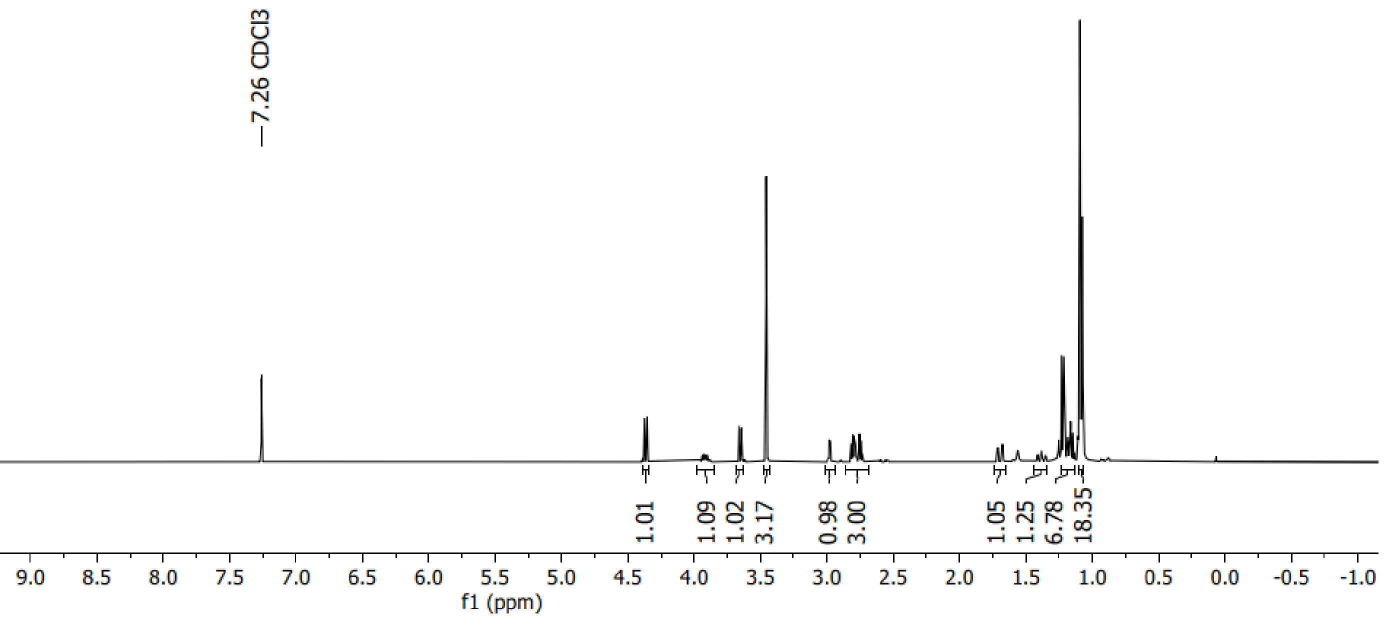

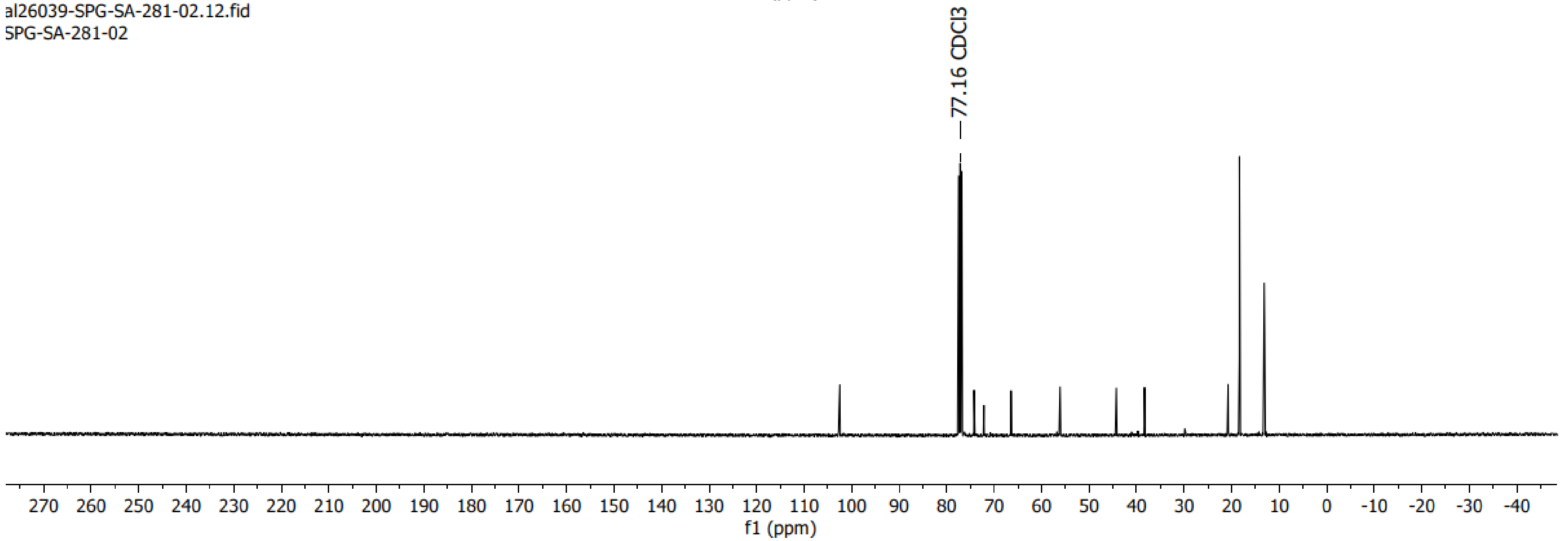

jn15057-SPG-SA-336-01.10.fid
SPG-SA-336-01

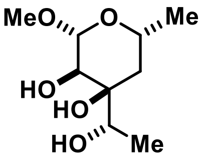

11 in $CDCl_3$
^{1}H (400 MHz)
$^{13}C\{^{1}H\}$ (101 MHz)

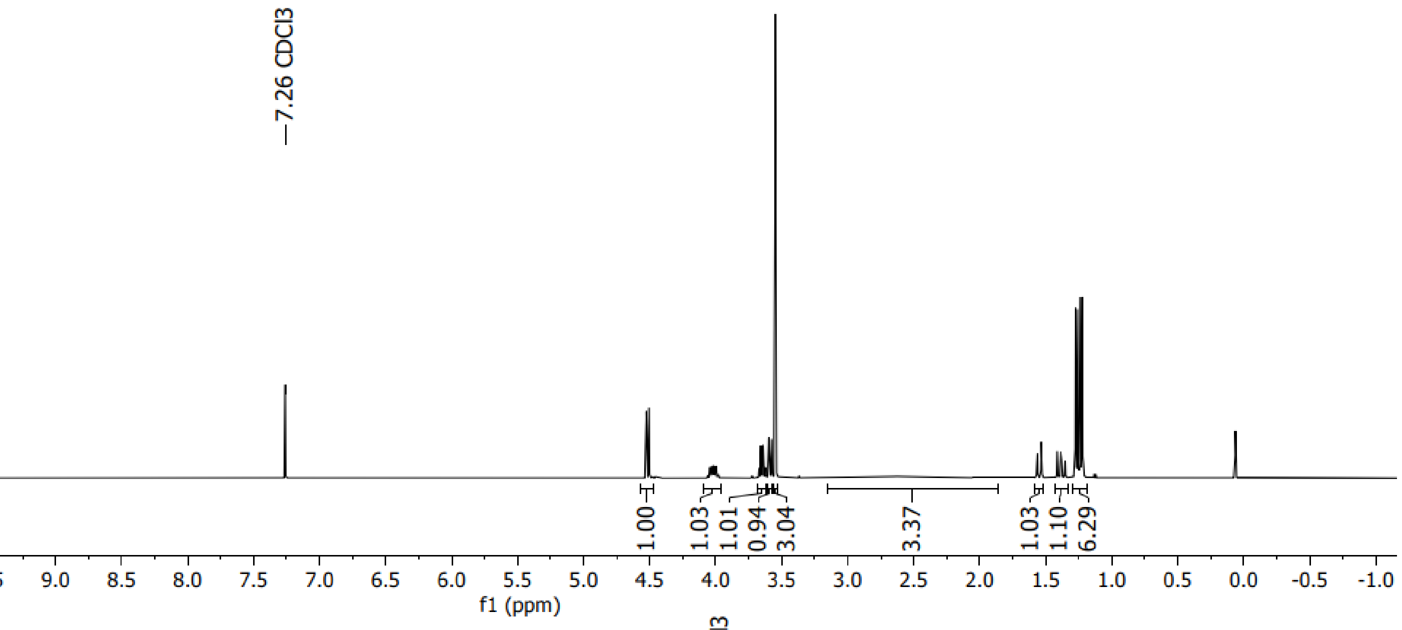

jn15057-SPG-SA-336-01.12.fid
SPG-SA-336-01

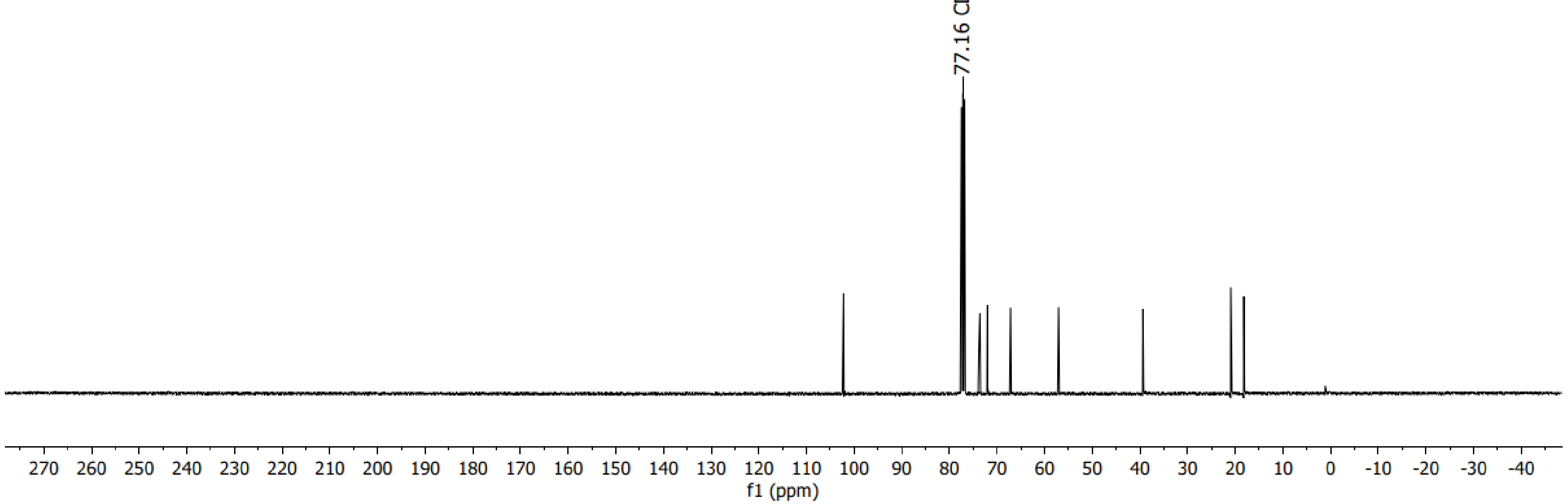

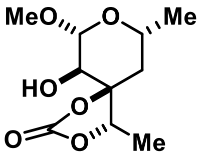

12 in $CDCl_3$
^1H (400 MHz)
$^{13}C\{^1H\}$ (101 MHz)

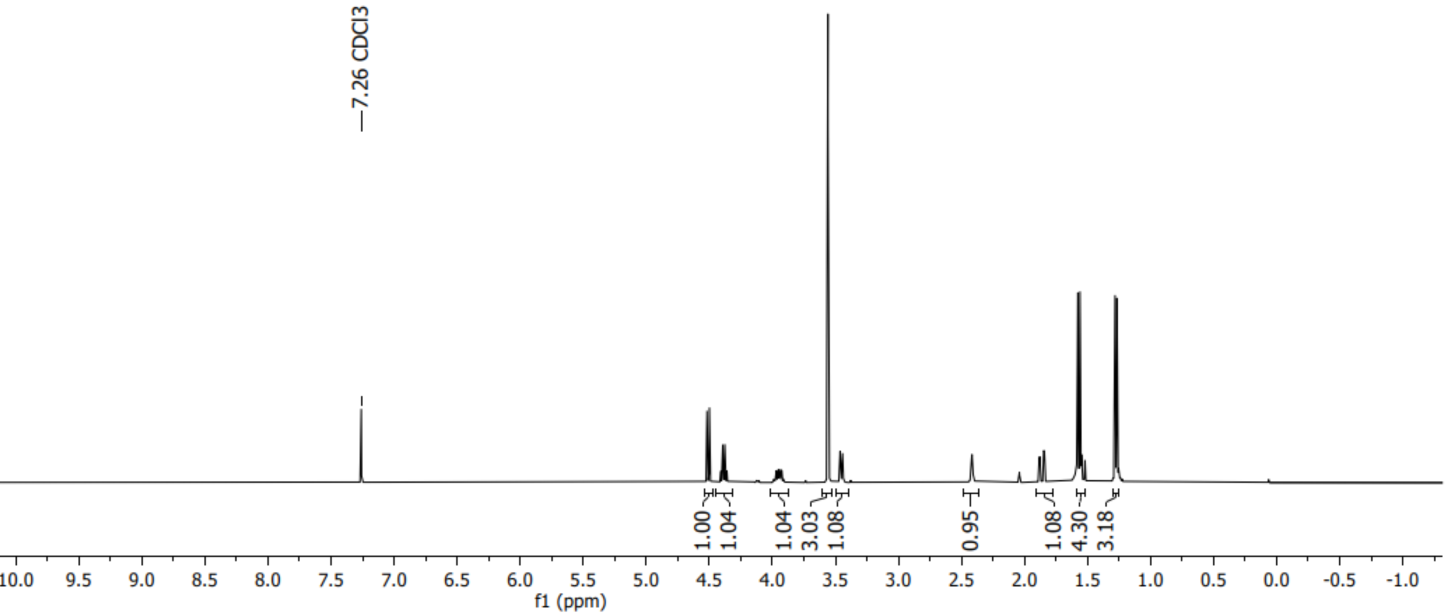

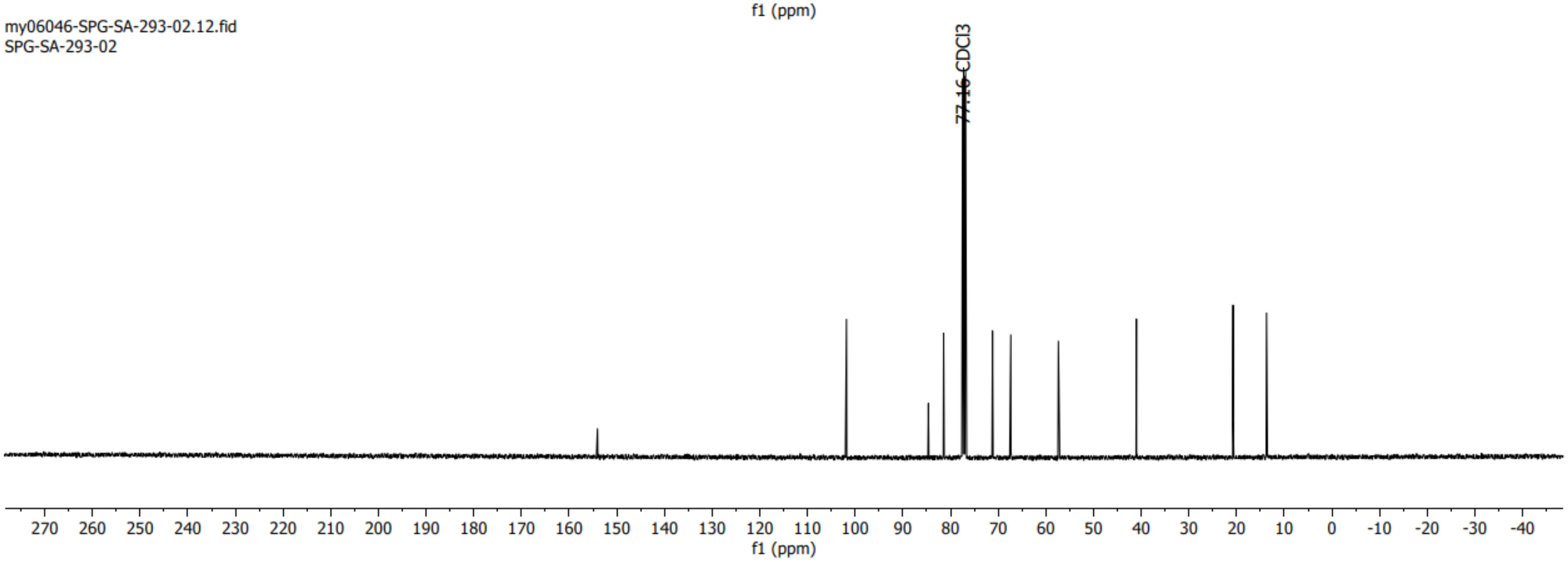

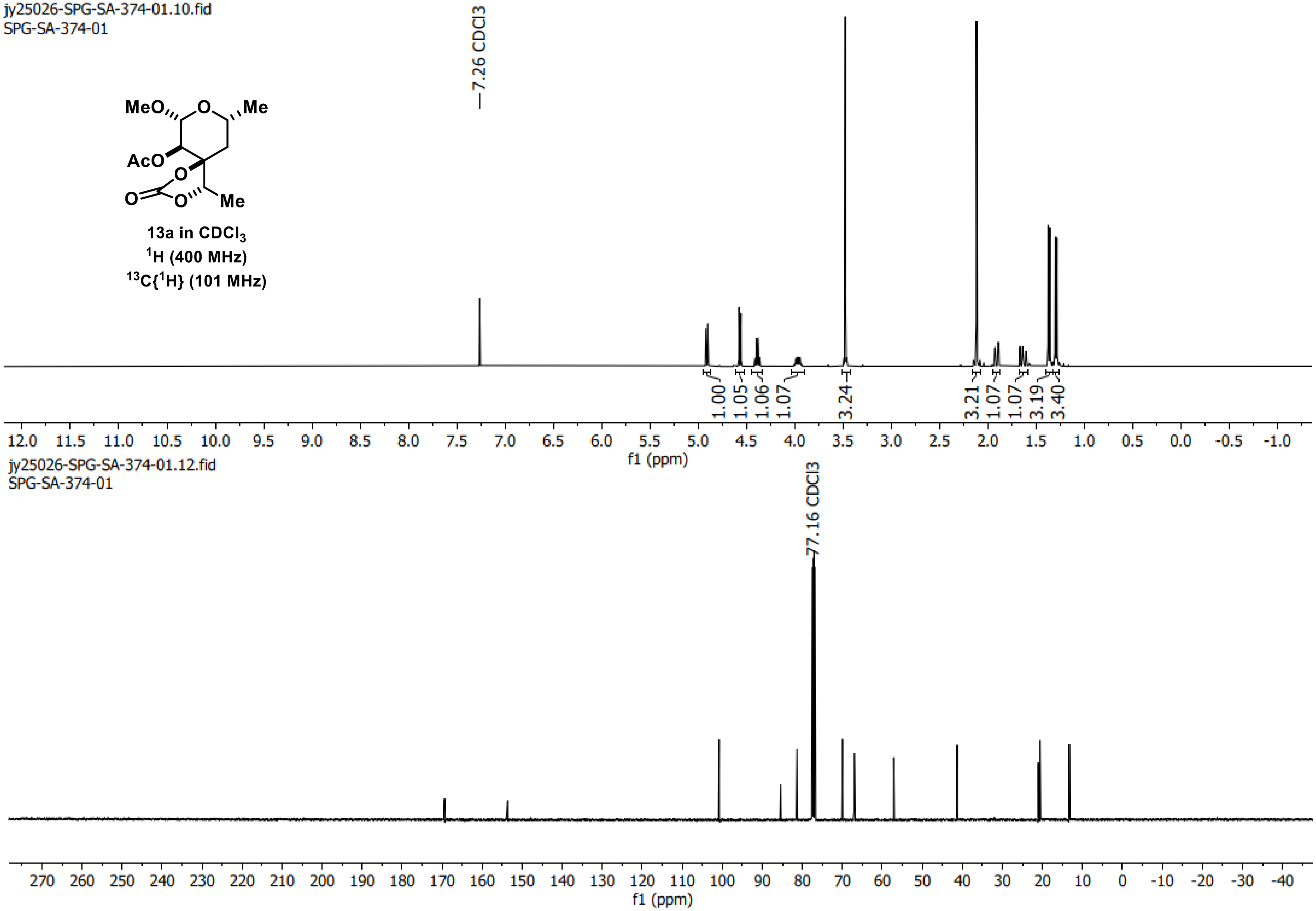
jy25026-SPG-SA-374-01.10.fid
SPG-SA-374-01
MeO
O
Me
AcO
O
O
O
Me
13a in CDCl3
1H (400 MHz)
13C{1H} (101 MHz)
7.26 CDCl3
1.00
1.05
1.06
1.07
3.24
3.21
1.07
1.07
3.19
3.40
f1 (ppm)
jy25026-SPG-SA-374-01.12.fid
SPG-SA-374-01
77.16 CDCl3
f1 (ppm)

mz13022-SPG-SA-561-01.10.fid
SPG-SA-561-01

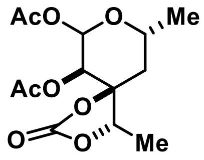

13b in $CDCl_3$
^{1}H (400 MHz)
$^{13}C\{^{1}H\}$ (101 MHz)

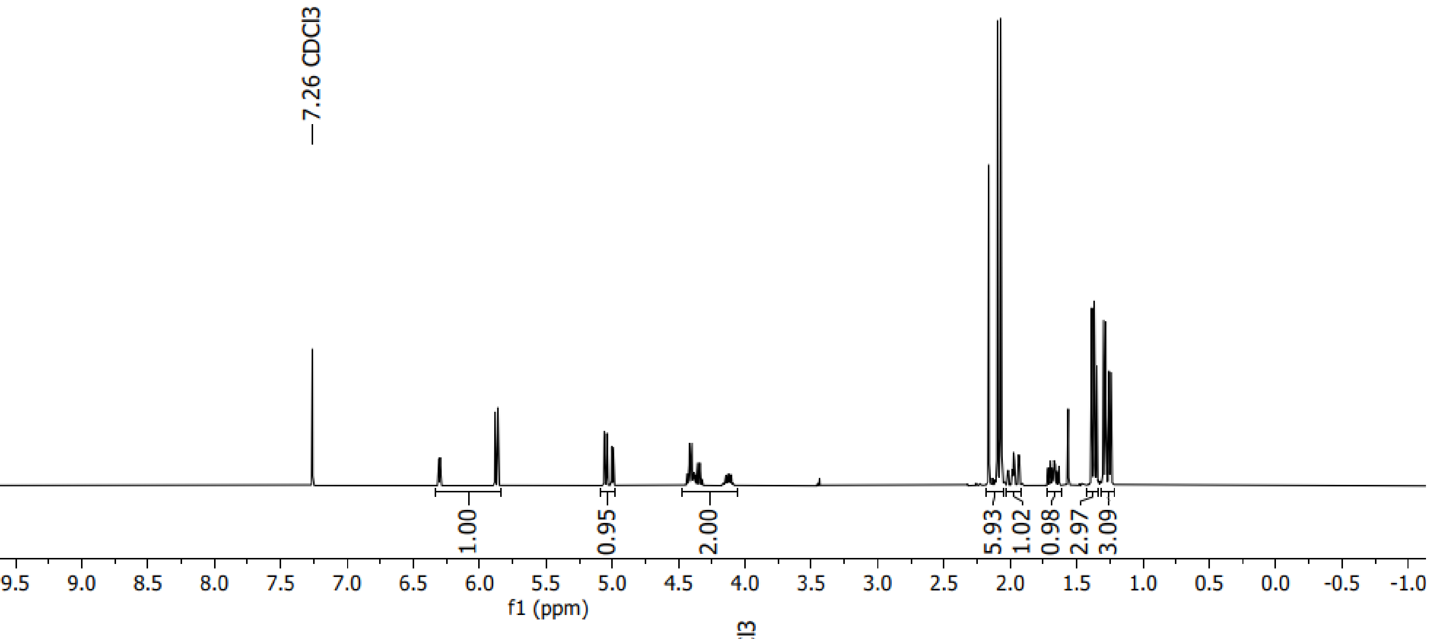

mz12042-SPG-SA-561-01.12.fid
SPG-SA-561-01

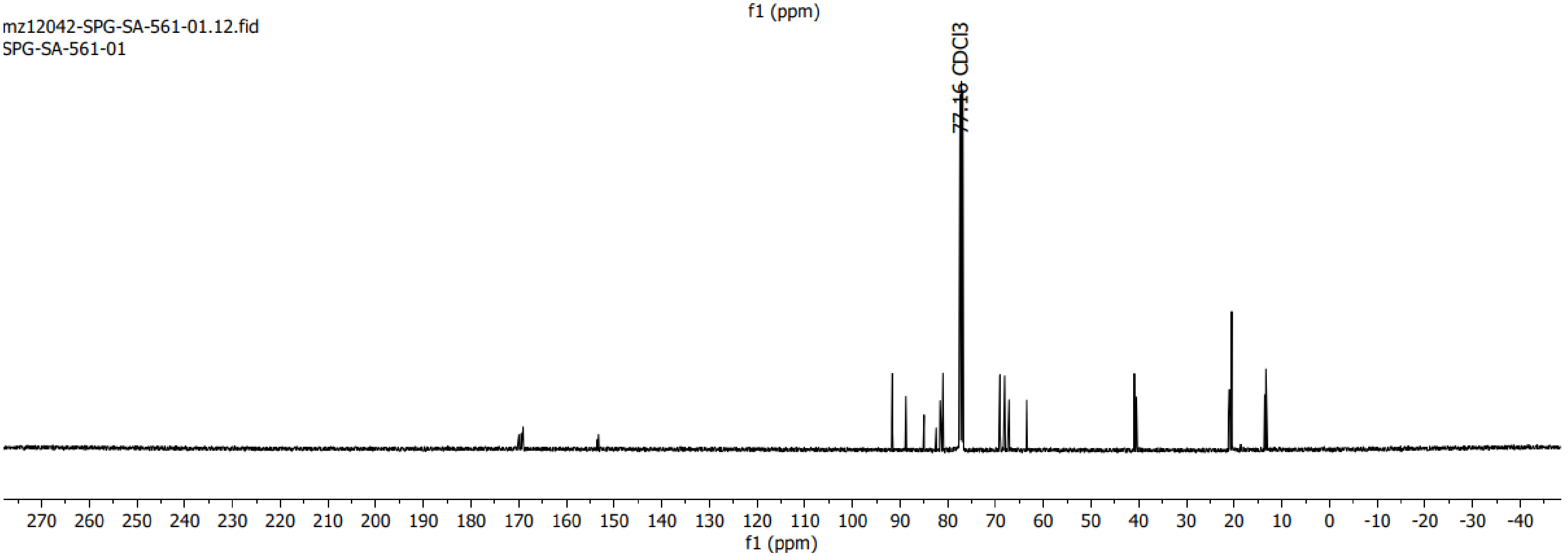

S1 in $CDCl_3$
^1H (400 MHz)
$^{13}C\{^1H\}$ (101 MHz)

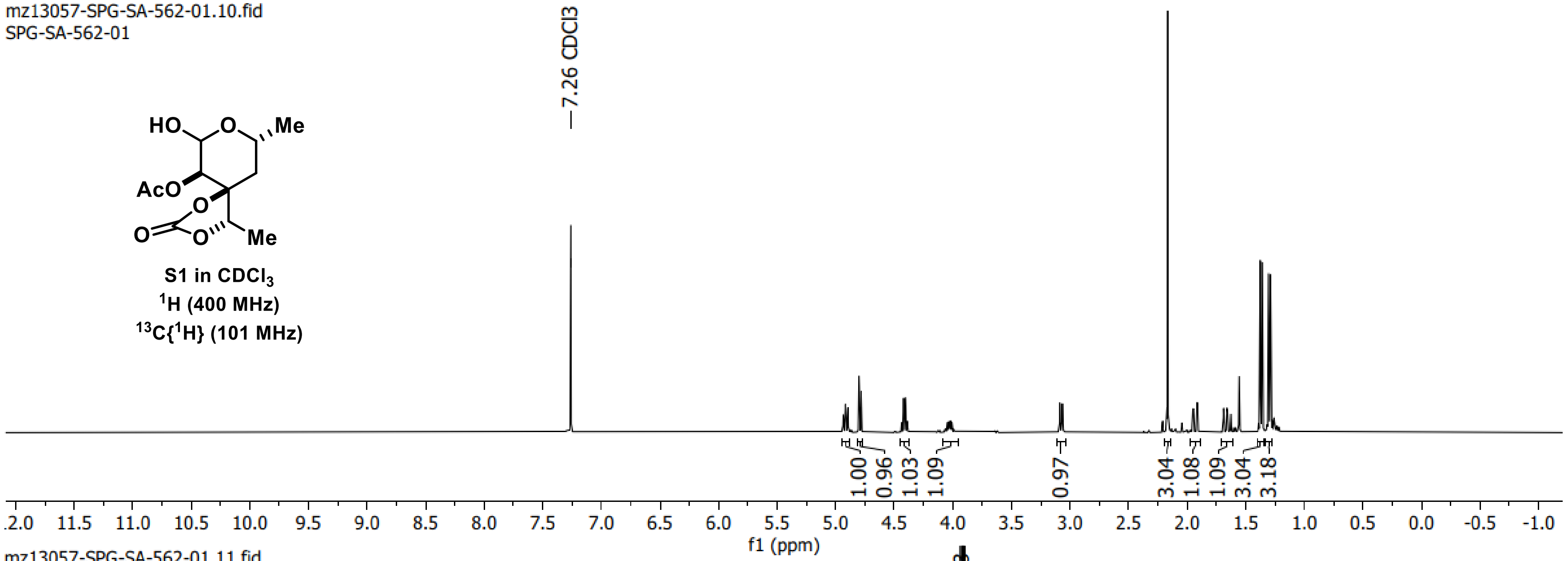

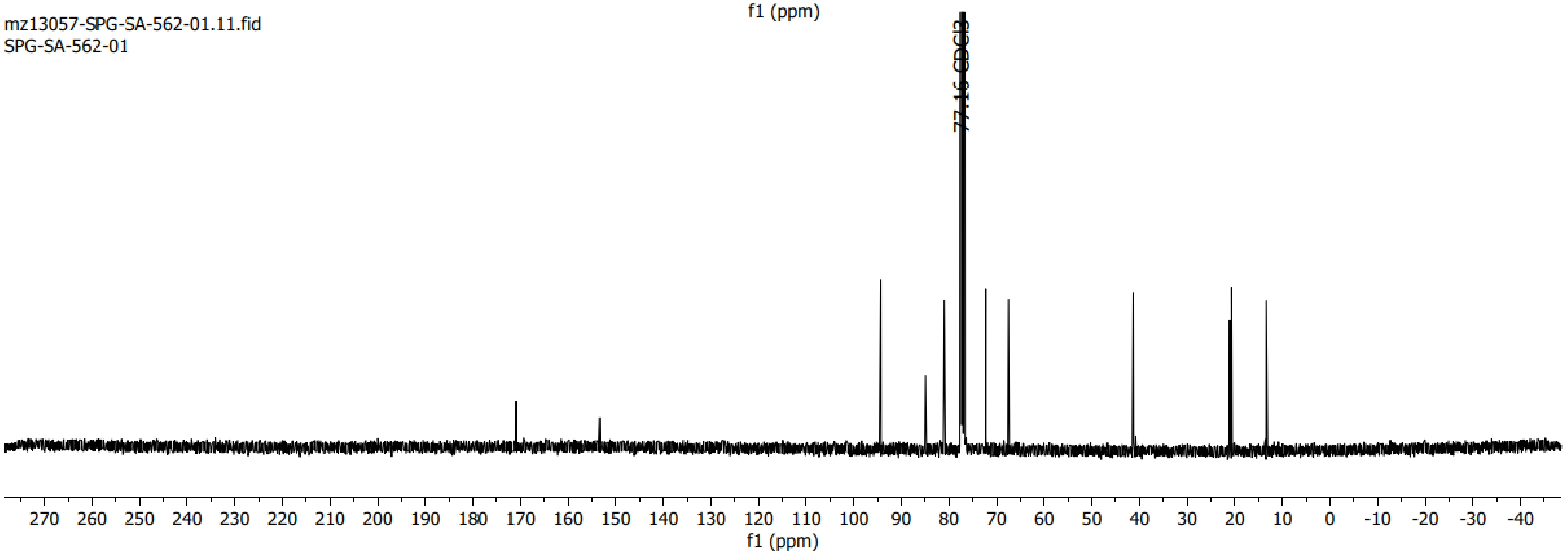

at13056-SPG-SA-389-02.10.fid
SPG-SA-389-02

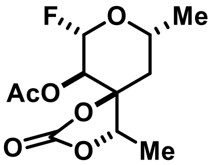

13c in $CDCl_3$
^{1}H (400 MHz)
$^{13}C\{^{1}H\}$ (101 MHz)

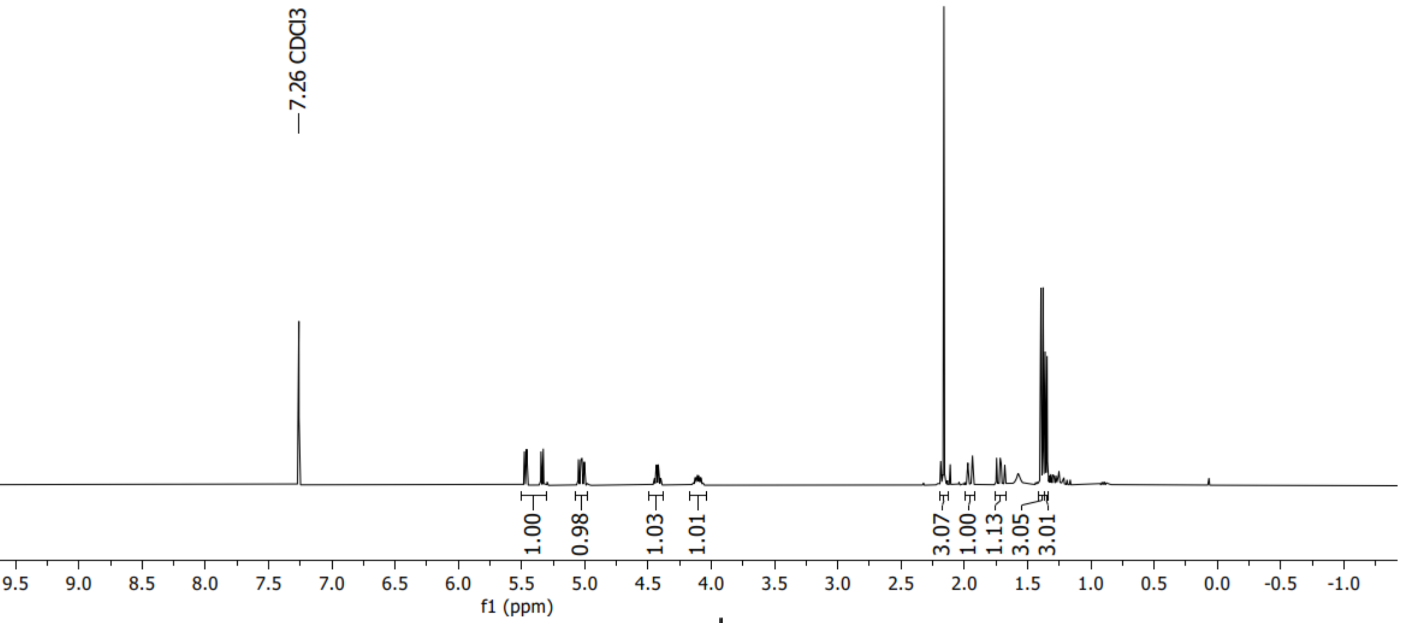

at13056-SPG-SA-389-02.12.fid
SPG-SA-389-02

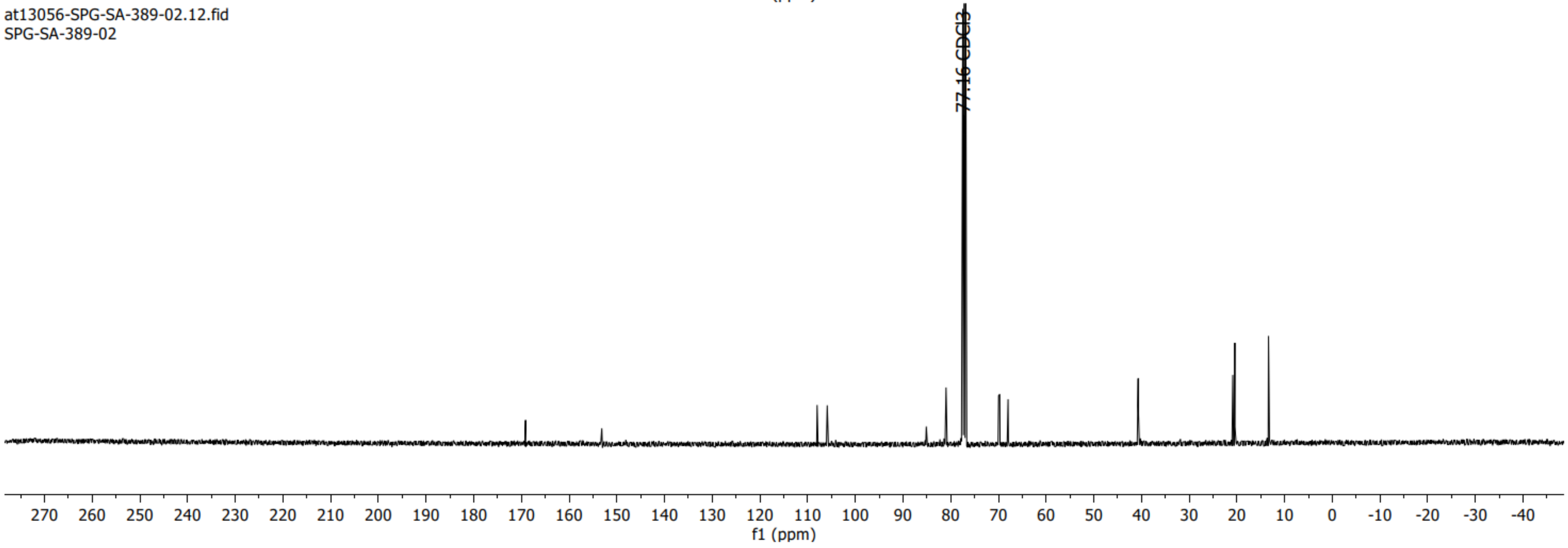

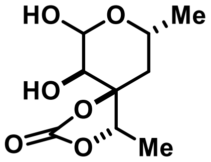

Aldgarose in [D$_4$]-methanol

^{1}H (400 MHz)

^{13}C{^{1}H} (101 MHz)

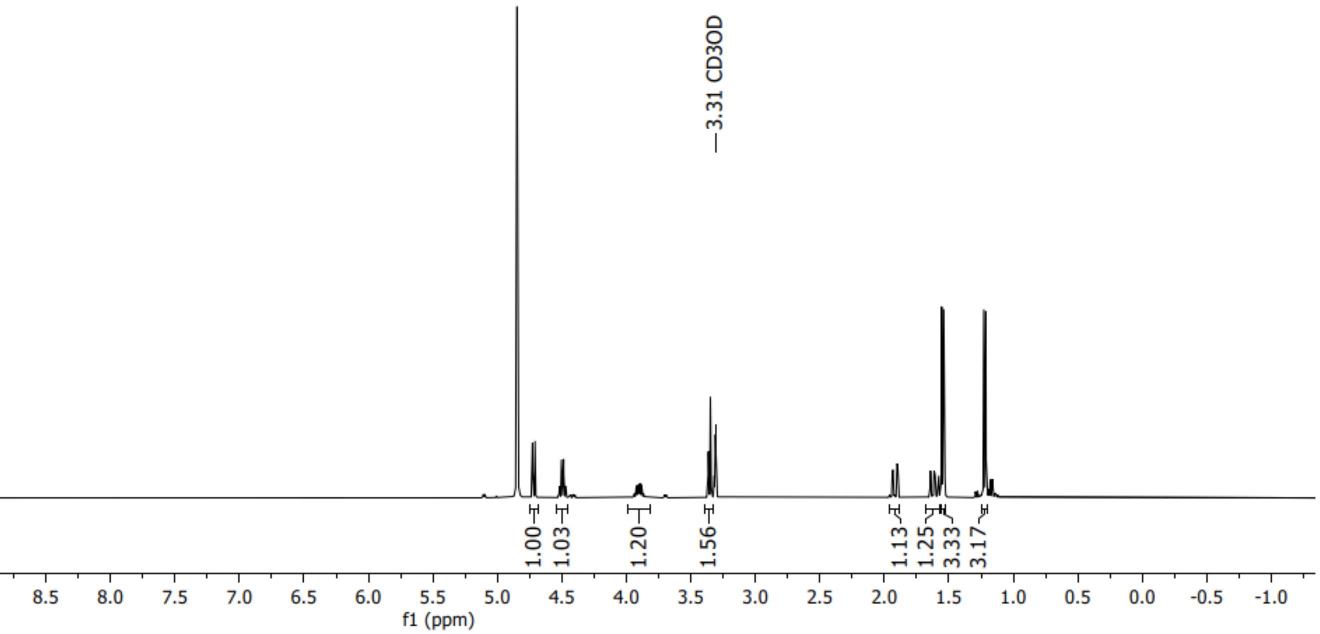

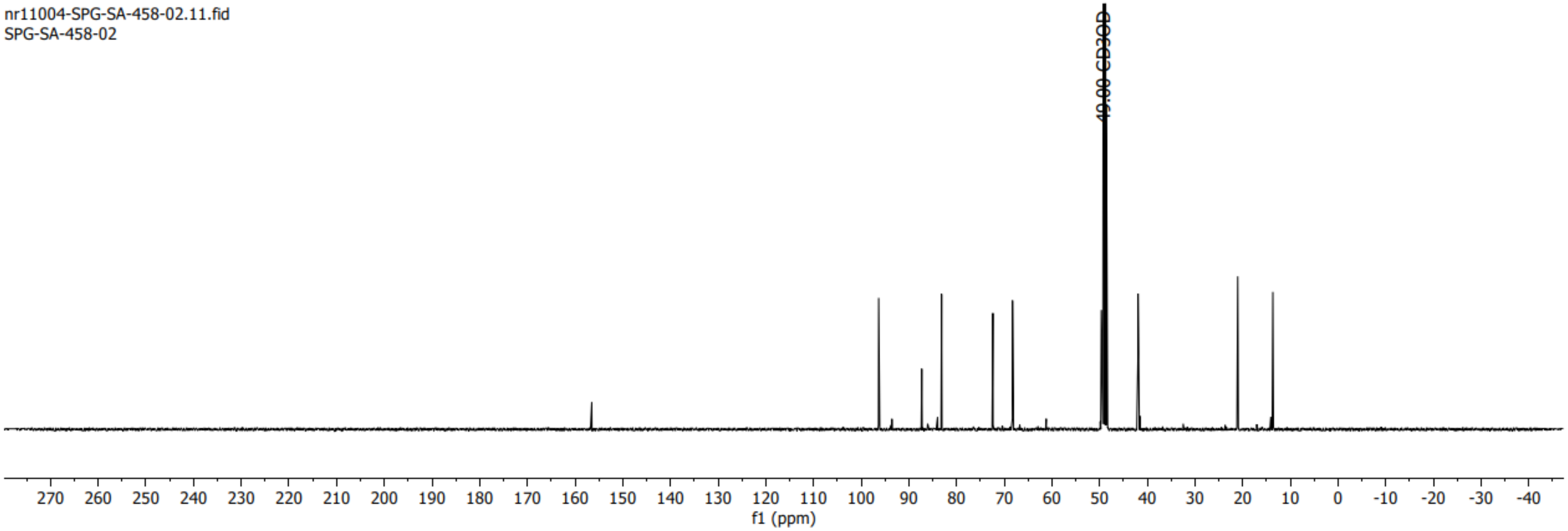

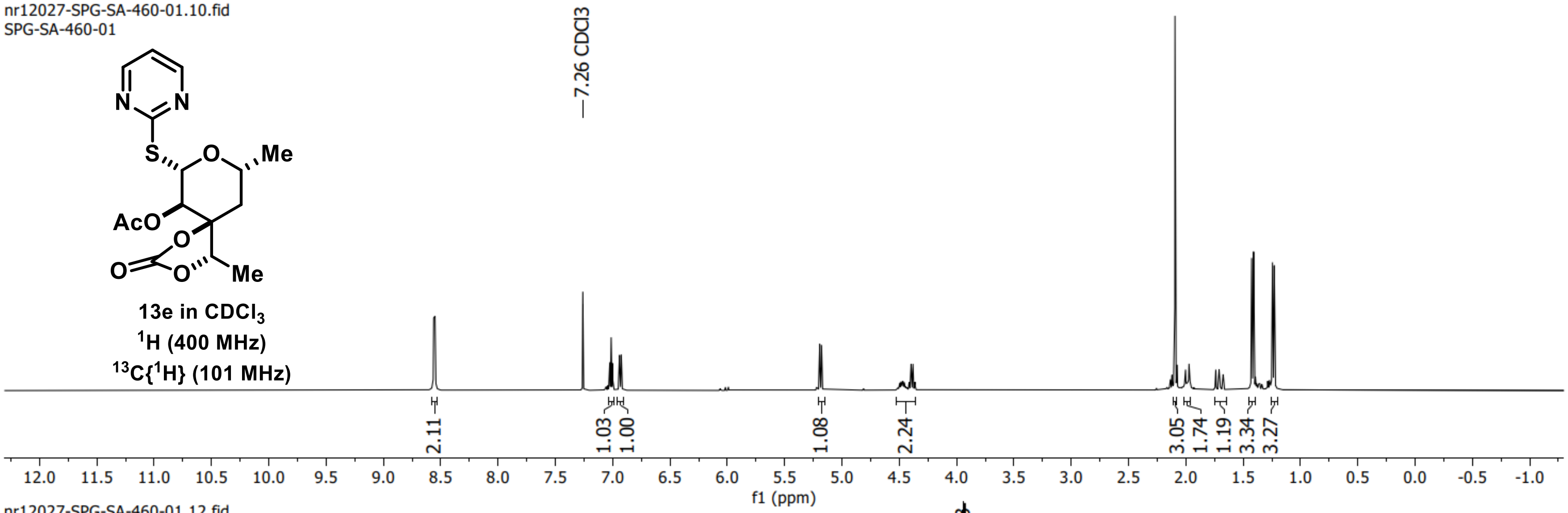

13e in $CDCl_3$
^{1}H (400 MHz)
$^{13}C\{^{1}H\}$ (101 MHz)

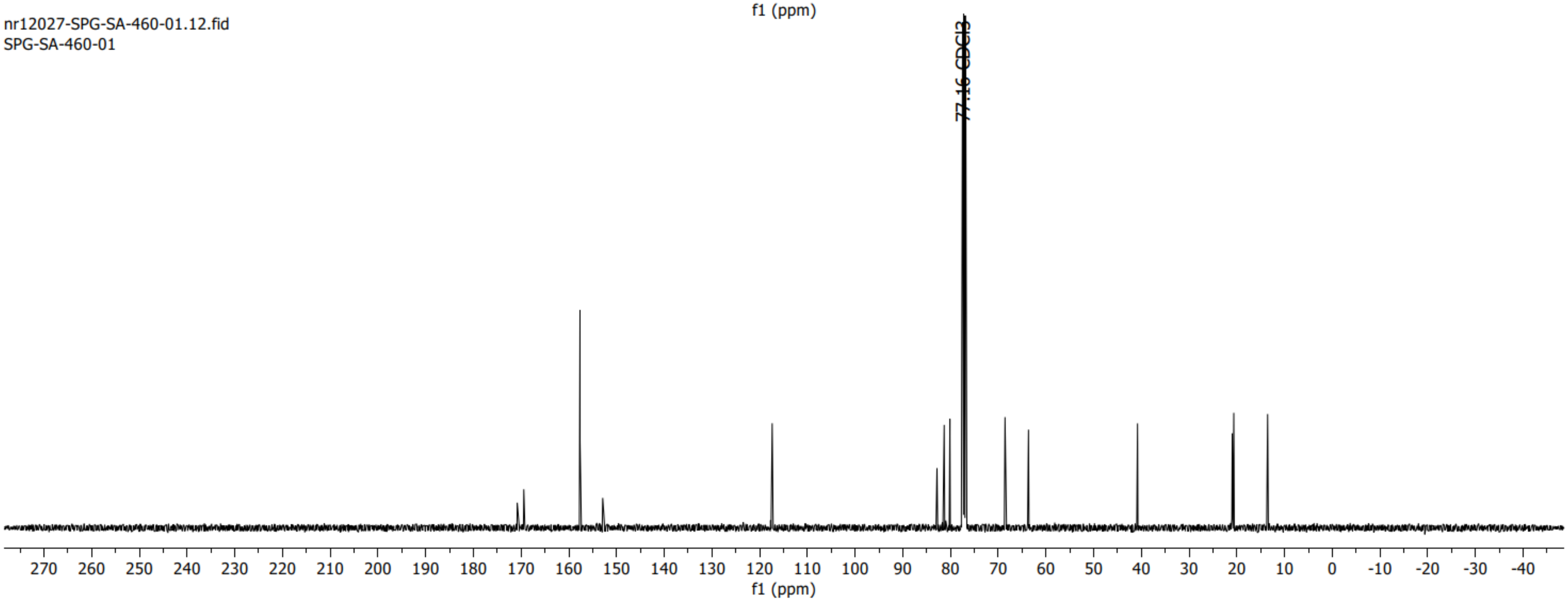

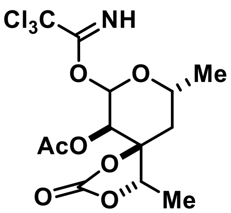

13g in $CDCl_3$
^1H (400 MHz)
$^{13}C\{^1H\}$ (101 MHz)

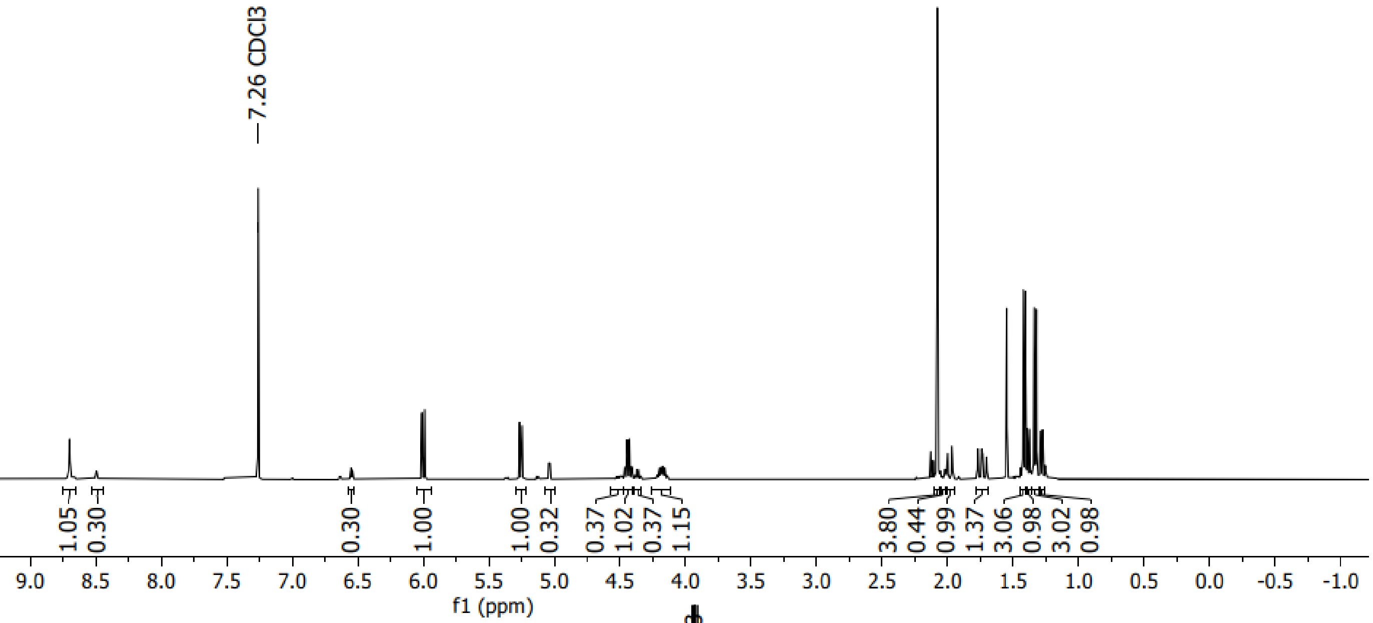

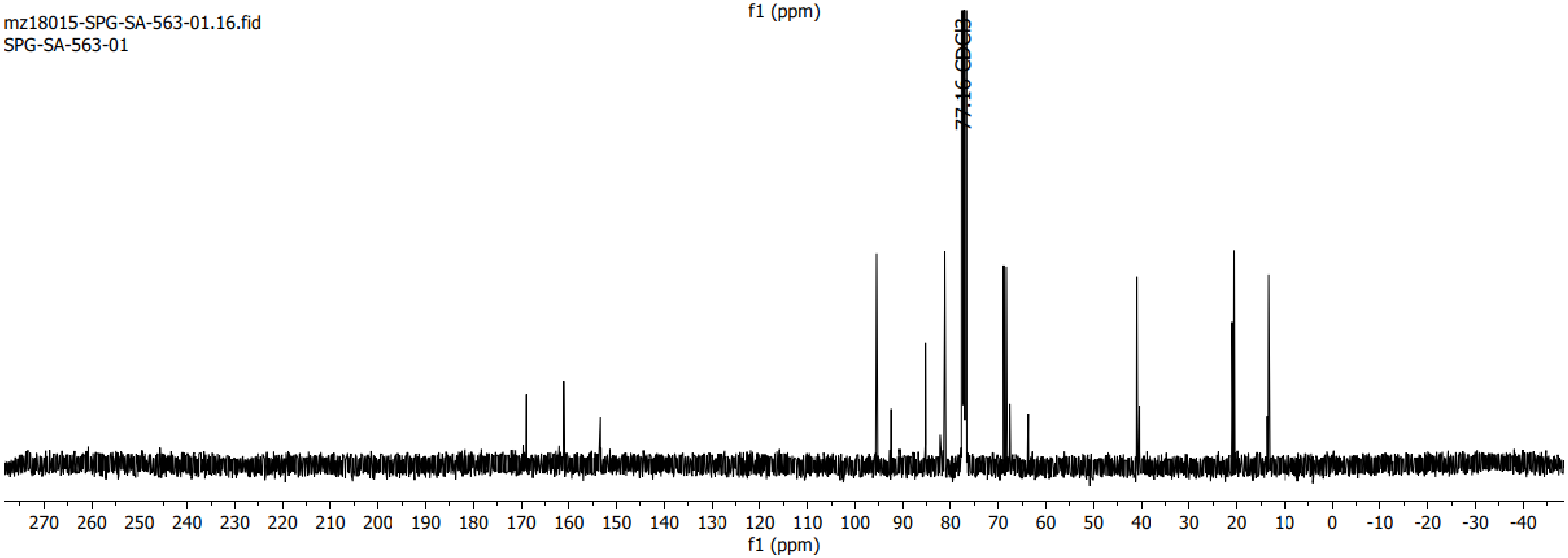

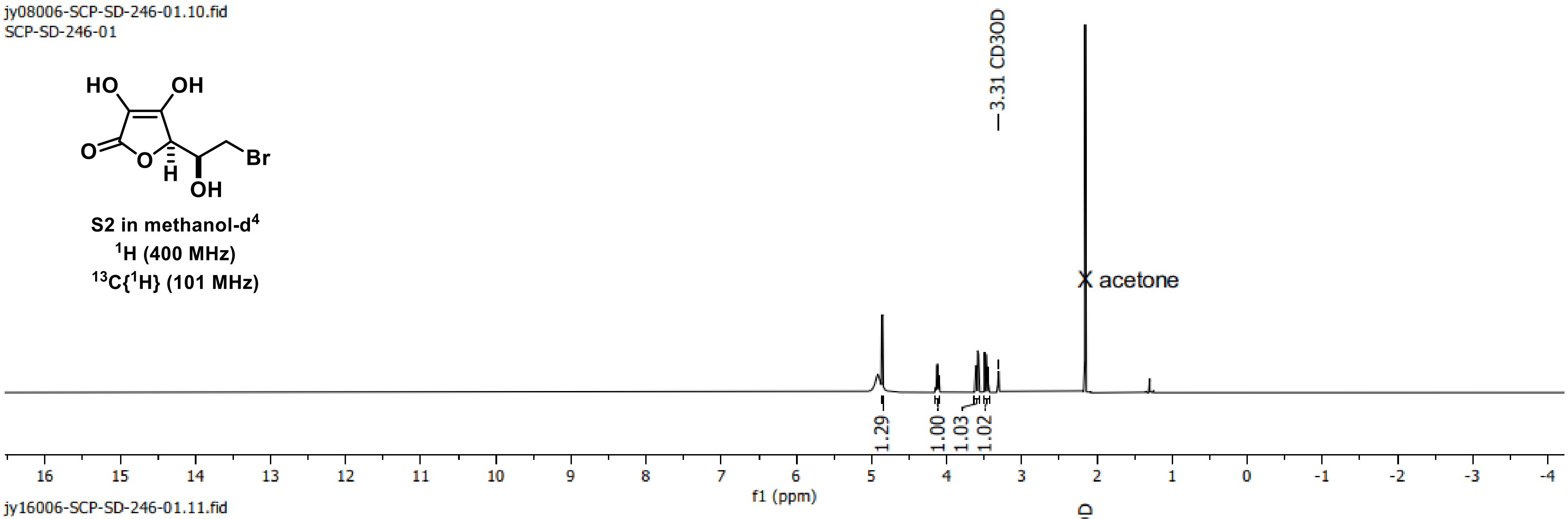
HO
OH
O
O
H
OH
Br
S2 in methanol-d4
1H (400 MHz)
13C{1H} (101 MHz)
jy08006-SCP-SD-246-01.10.fid
SCP-SD-246-01
3.31 CD3OD
acetone
1.29
1.00
1.03
1.02
16
15
14
13
12
11
10
9
8
7
6
5
4
3
2
1
0
-1
-2
-3
-4
f1 (ppm)

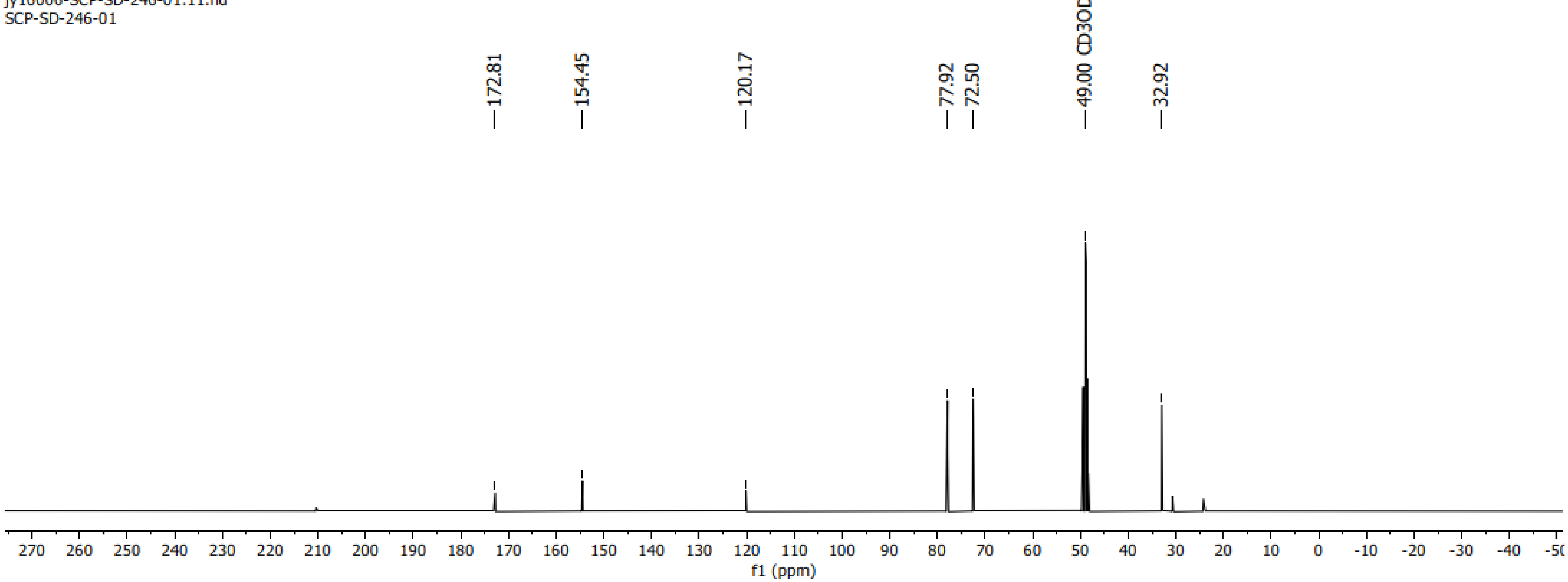
jy16006-SCP-SD-246-01.11.fid
SCP-SD-246-01
172.81
154.45
120.17
77.92
72.50
49.00 CD3OD
32.92
270
260
250
240
230
220
210
200
190
180
170
160
150
140
130
120
110
100
90
80
70
60
50
40
30
20
10
0
-10
-20
-30
-40
f1 (ppm)

mz13013-SPG-SA-558-01.10.fid
SPG-SA-558-01

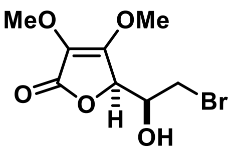

22 in $CDCl_3$
^{1}H (400 MHz)
$^{13}C\{^1H\}$ (101 MHz)

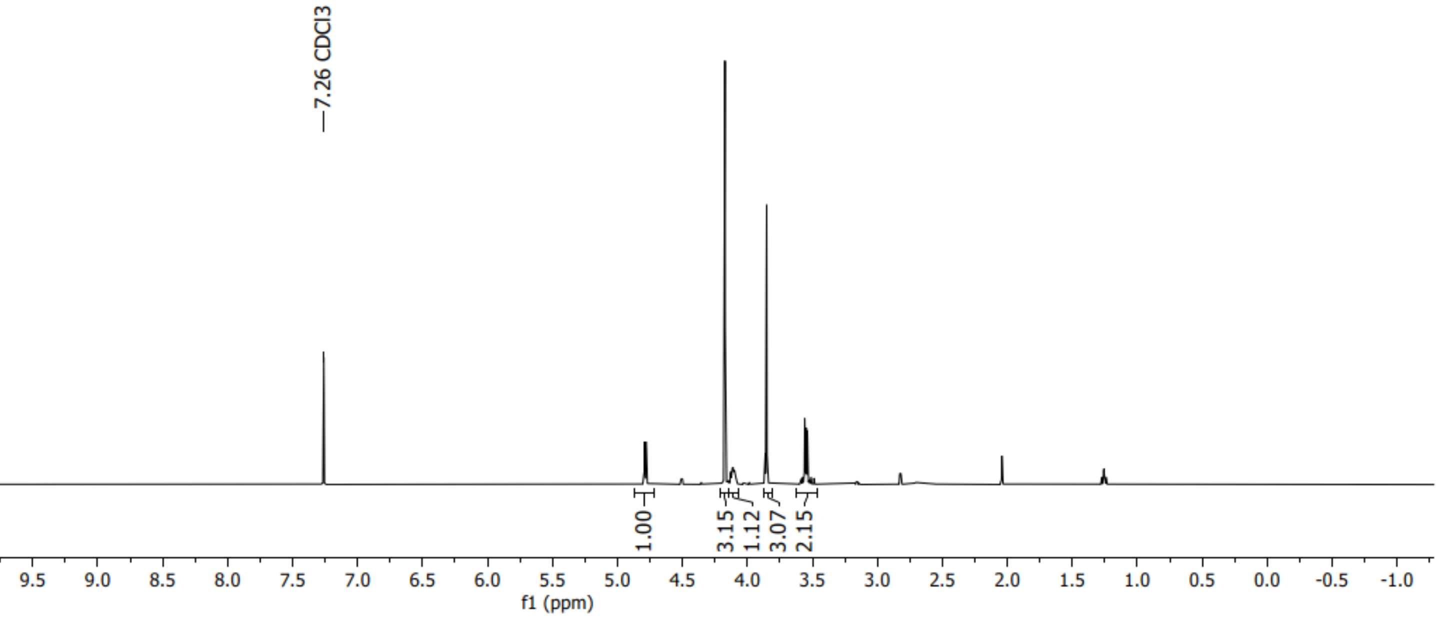

mz13013-SPG-SA-558-01.11.fid
SPG-SA-558-01

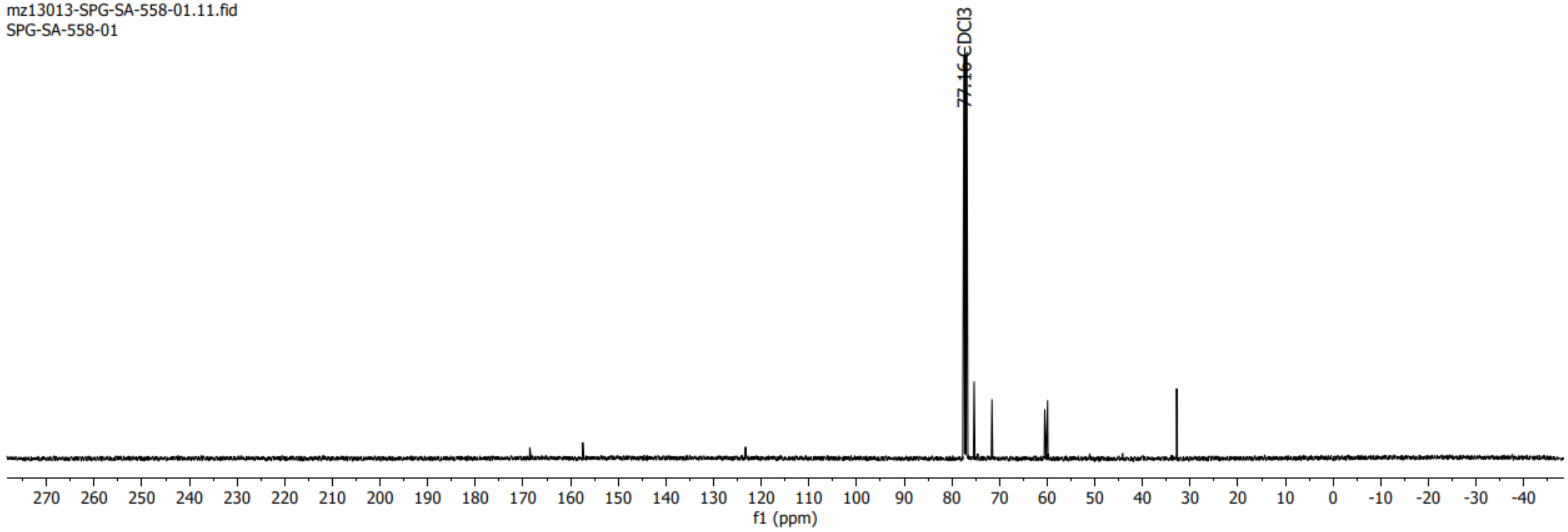

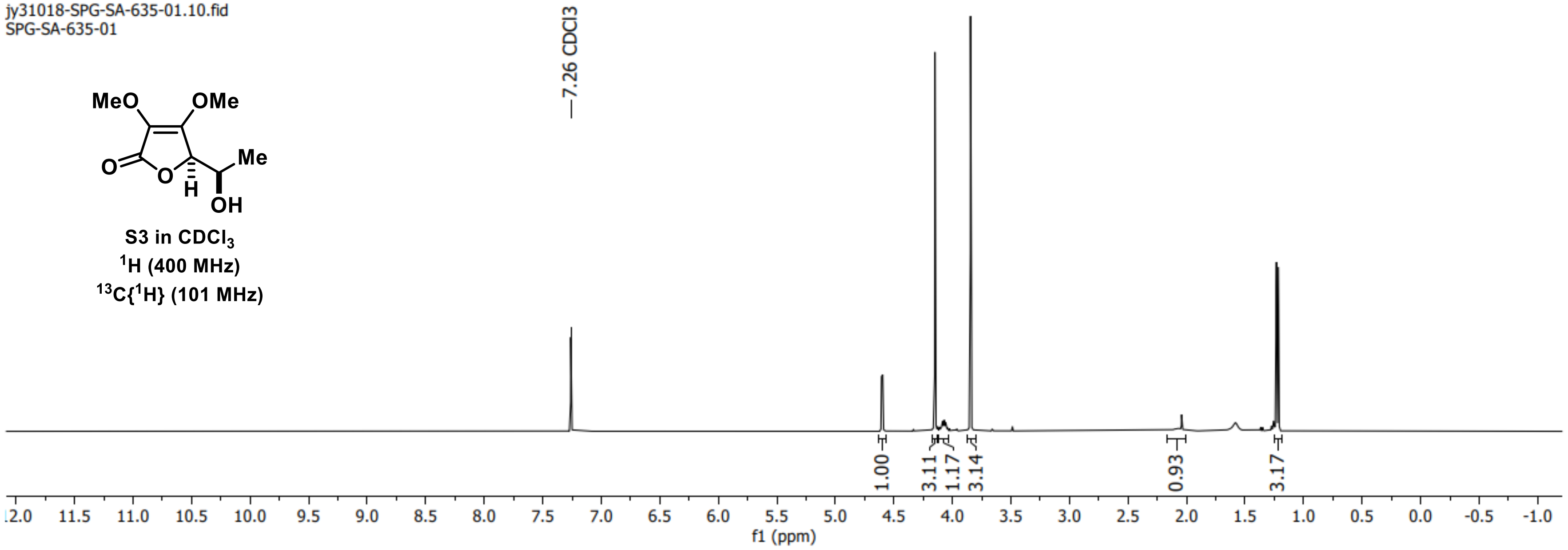
jy31018-SPG-SA-635-01.10.fid
SPG-SA-635-01
MeO
OMe
O
O
H
Me
OH
S3 in CDCl3
1H (400 MHz)
13C{1H} (101 MHz)
7.26 CDCl3
1.00
3.11
1.17
3.14
0.93
3.17
f1 (ppm)

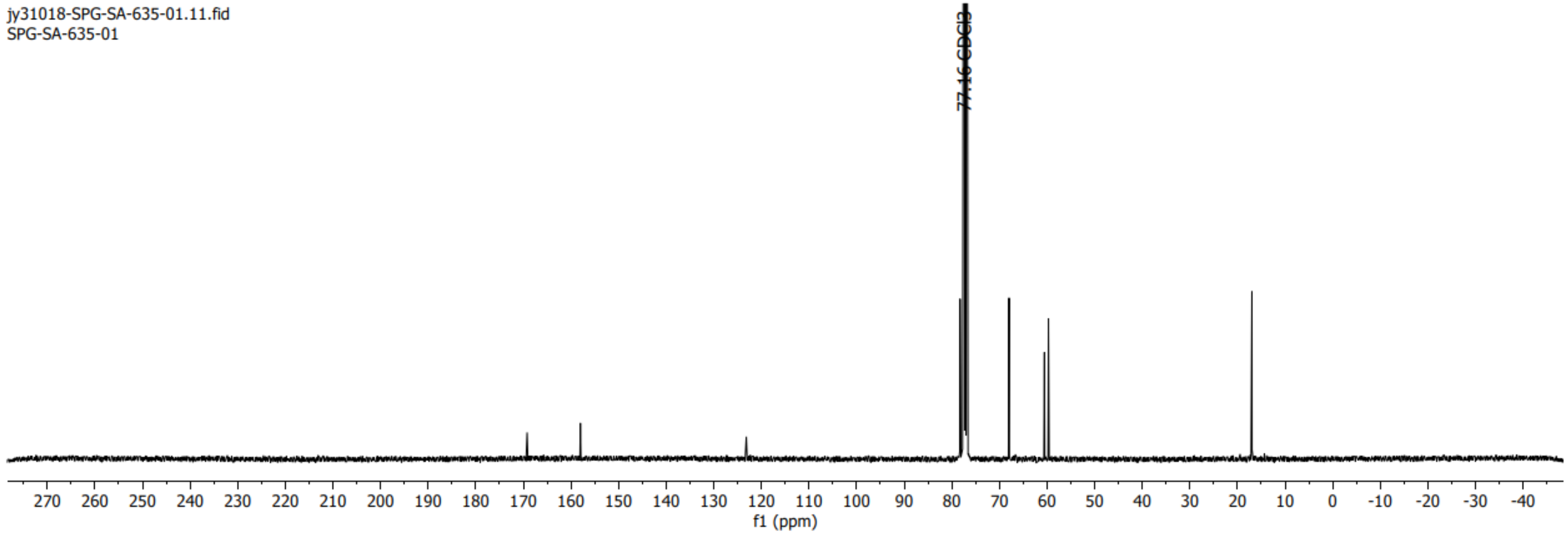
jy31018-SPG-SA-635-01.11.fid
SPG-SA-635-01
77.16 CDCl3
f1 (ppm)

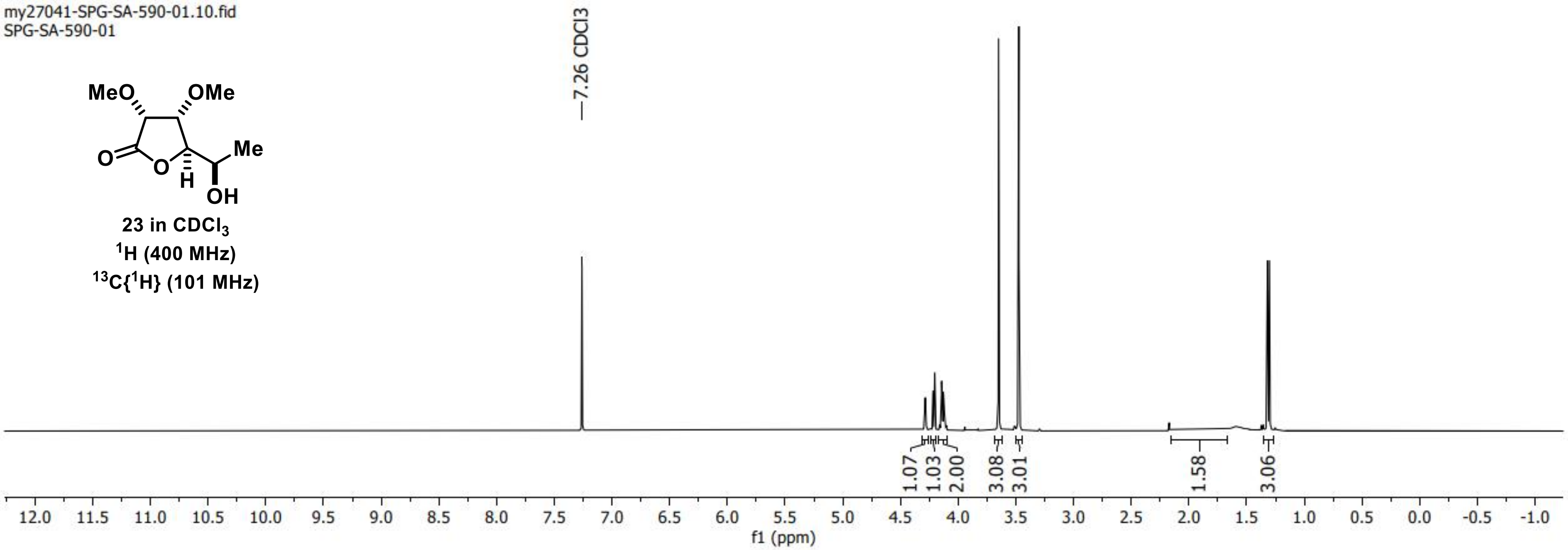

23 in $CDCl_3$
^1H (400 MHz)
$^{13}C\{^1H\}$ (101 MHz)

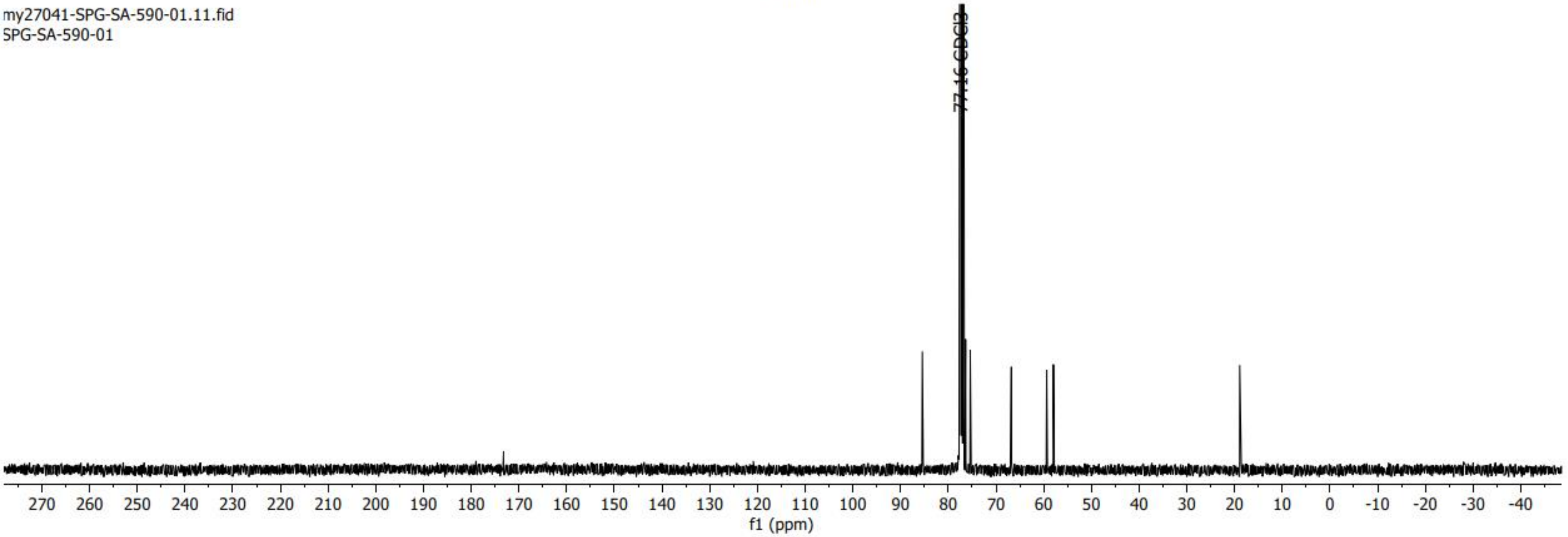

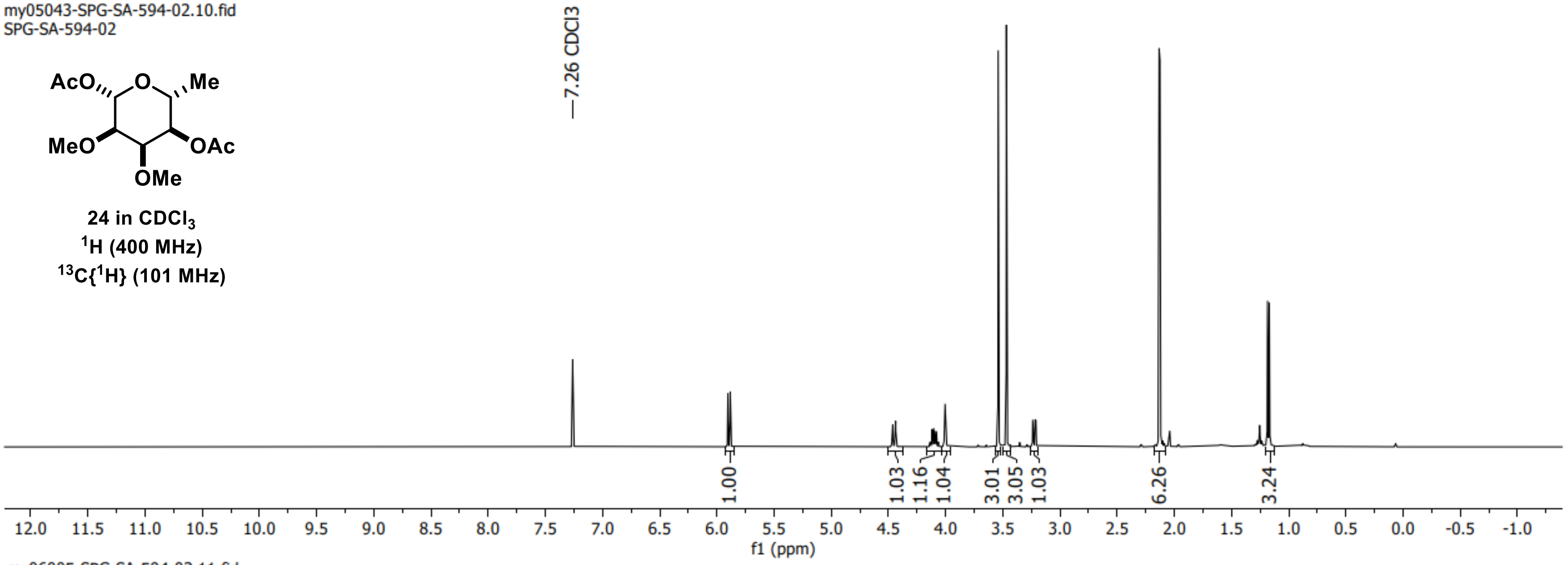
my05043-SPG-SA-594-02.10.fid
SPG-SA-594-02
AcO
O
Me
MeO
OAc
OMe
24 in CDCl3
1H (400 MHz)
13C{1H} (101 MHz)
7.26 CDCl3
1.00
1.03
1.16
1.04
3.01
3.05
1.03
6.26
3.24
12.0 11.5 11.0 10.5 10.0 9.5 9.0 8.5 8.0 7.5 7.0 6.5 6.0 5.5 5.0 4.5 4.0 3.5 3.0 2.5 2.0 1.5 1.0 0.5 0.0 -0.5 -1.0
f1 (ppm)

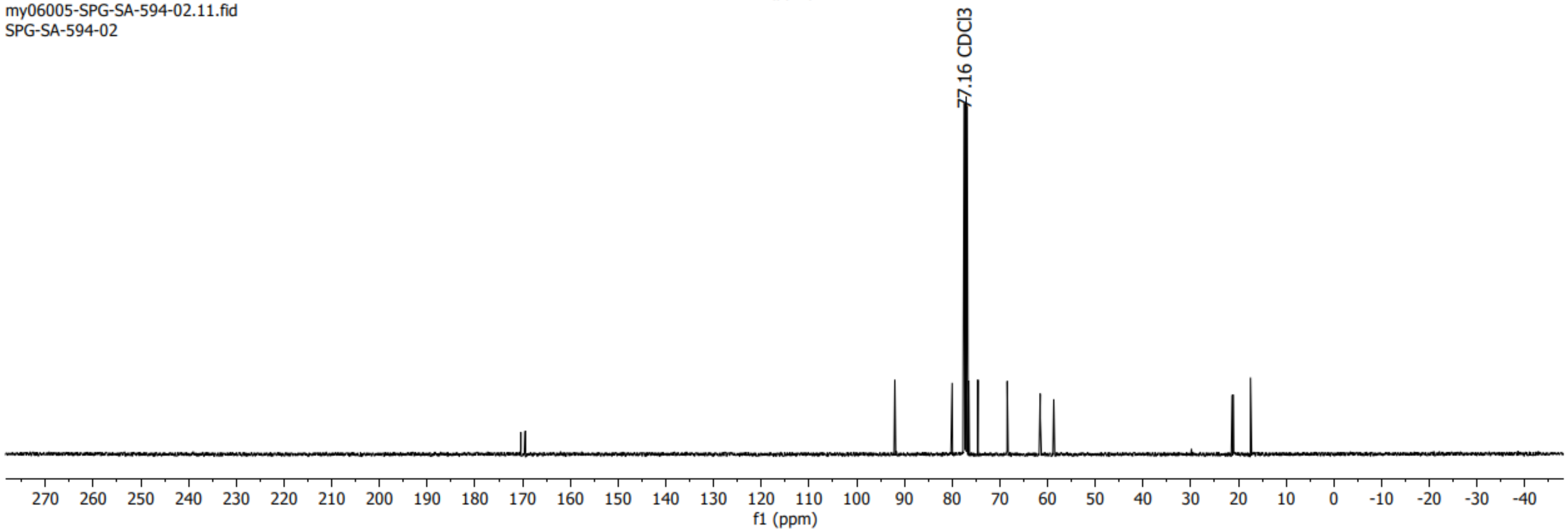
my06005-SPG-SA-594-02.11.fid
SPG-SA-594-02
77.16 CDCl3
270 260 250 240 230 220 210 200 190 180 170 160 150 140 130 120 110 100 90 80 70 60 50 40 30 20 10 0 -10 -20 -30 -40
f1 (ppm)

at25041-SPG-SA-653-02.10.fid
SPG-SA-653-02

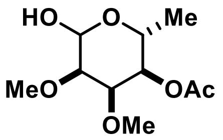

S4 in $CDCl_3$
^{1}H (400 MHz)
^{13}C{^{1}H} (101 MHz)

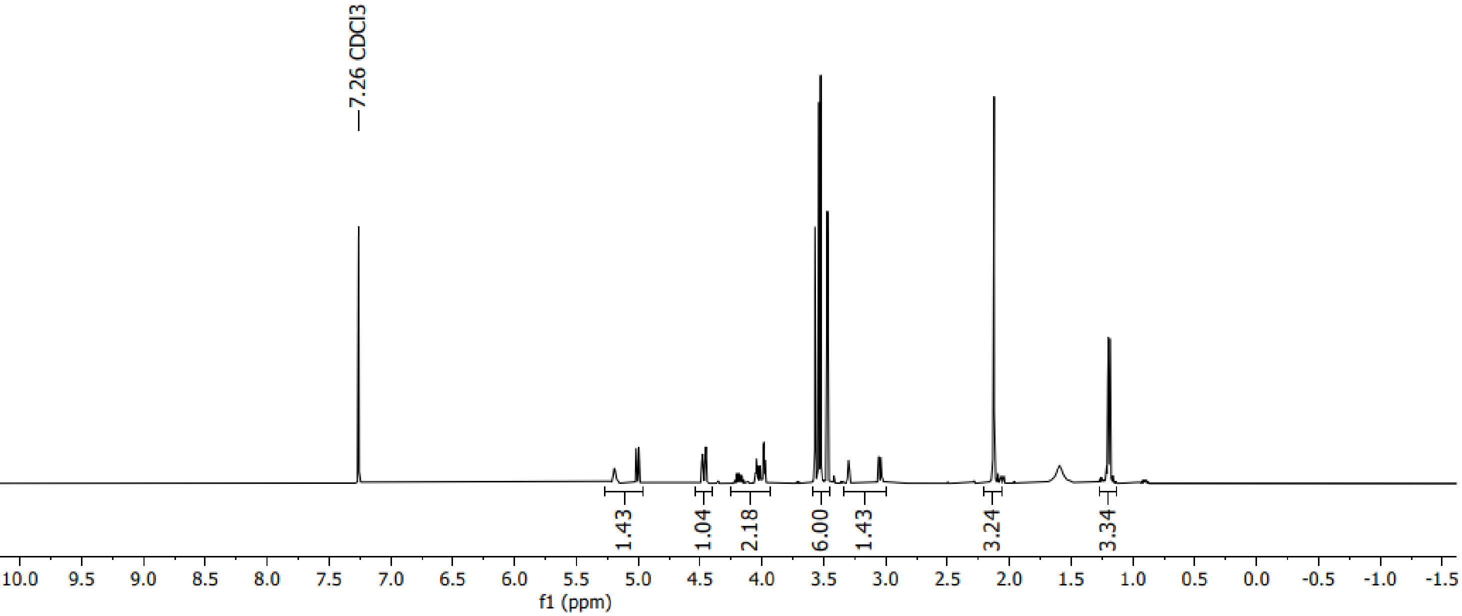

at25041-SPG-SA-653-02.11.fid
SPG-SA-653-02

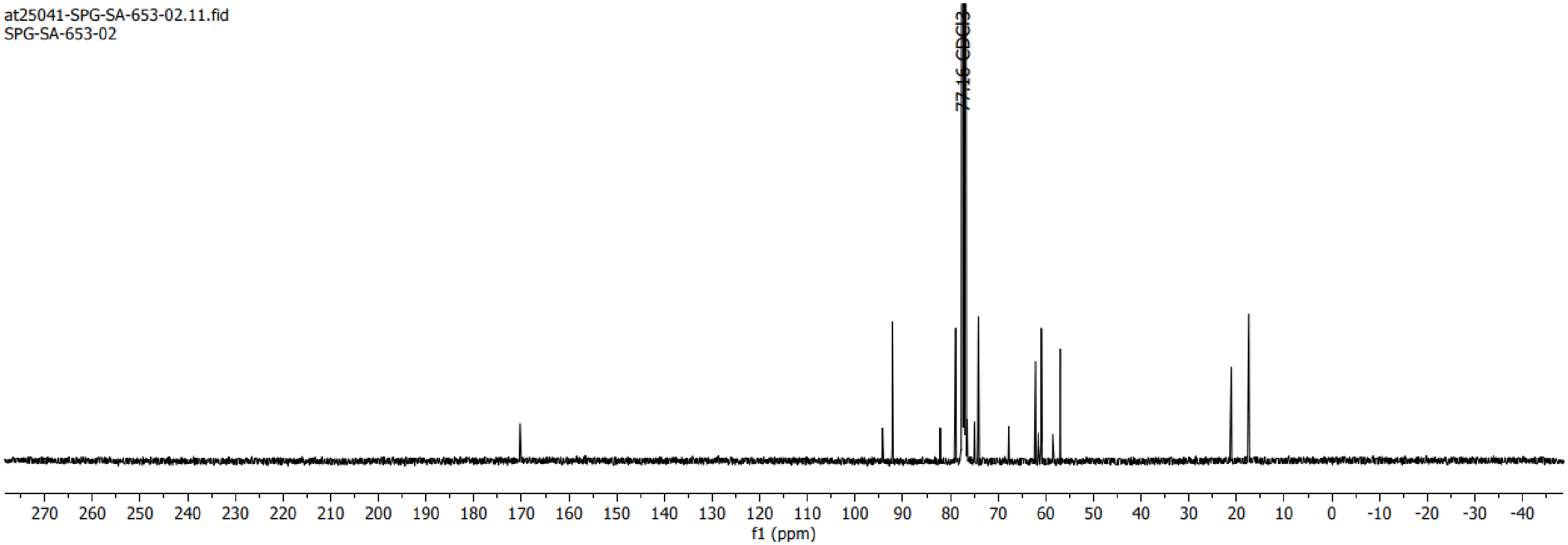

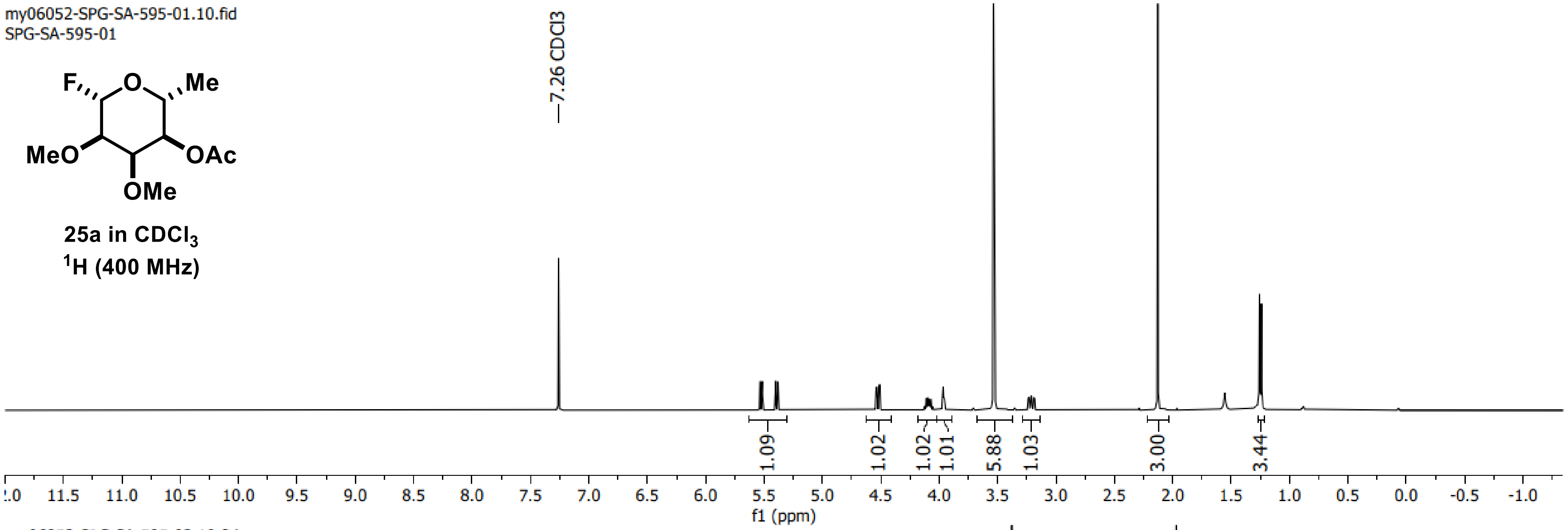
my06052-SPG-SA-595-01.10.fid
SPG-SA-595-01
25a in CDCl3
1H (400 MHz)
7.26 CDCl3
1.09
1.02
1.02
1.01
5.88
1.03
3.00
3.44
f1 (ppm)

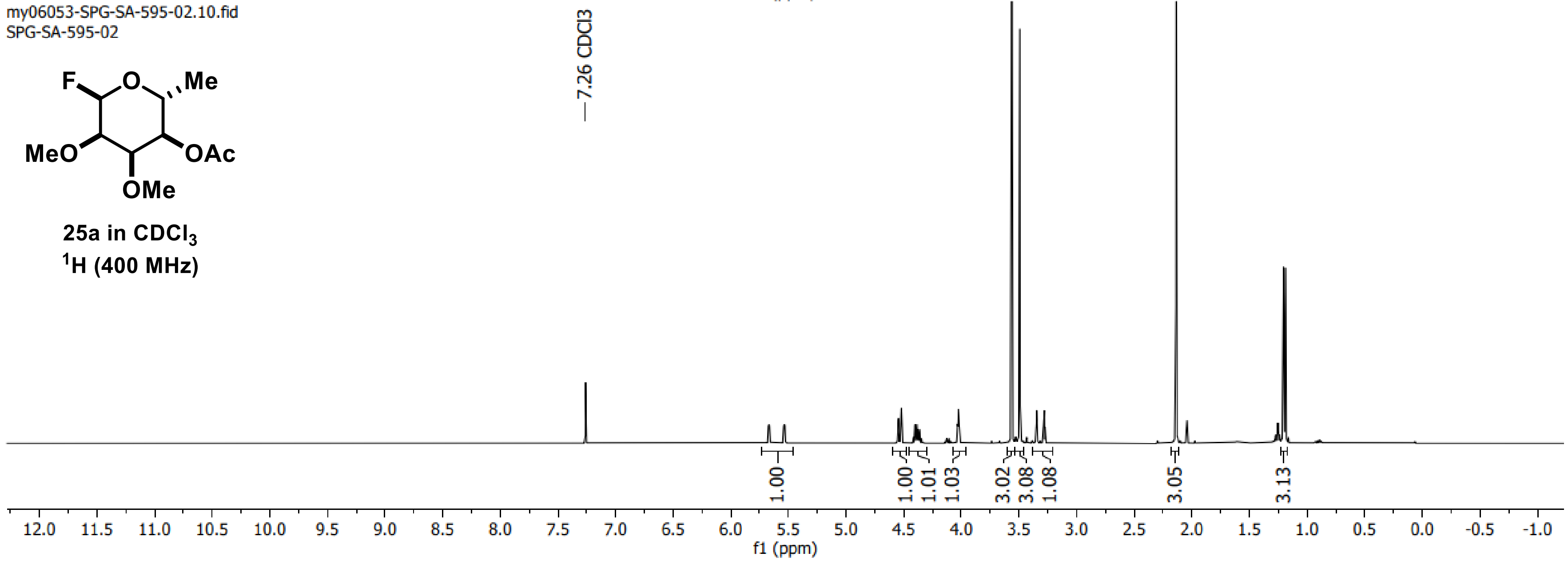
my06053-SPG-SA-595-02.10.fid
SPG-SA-595-02
25a in CDCl3
1H (400 MHz)
7.26 CDCl3
1.00
1.00
1.01
1.03
3.02
3.08
1.08
3.05
3.13
f1 (ppm)

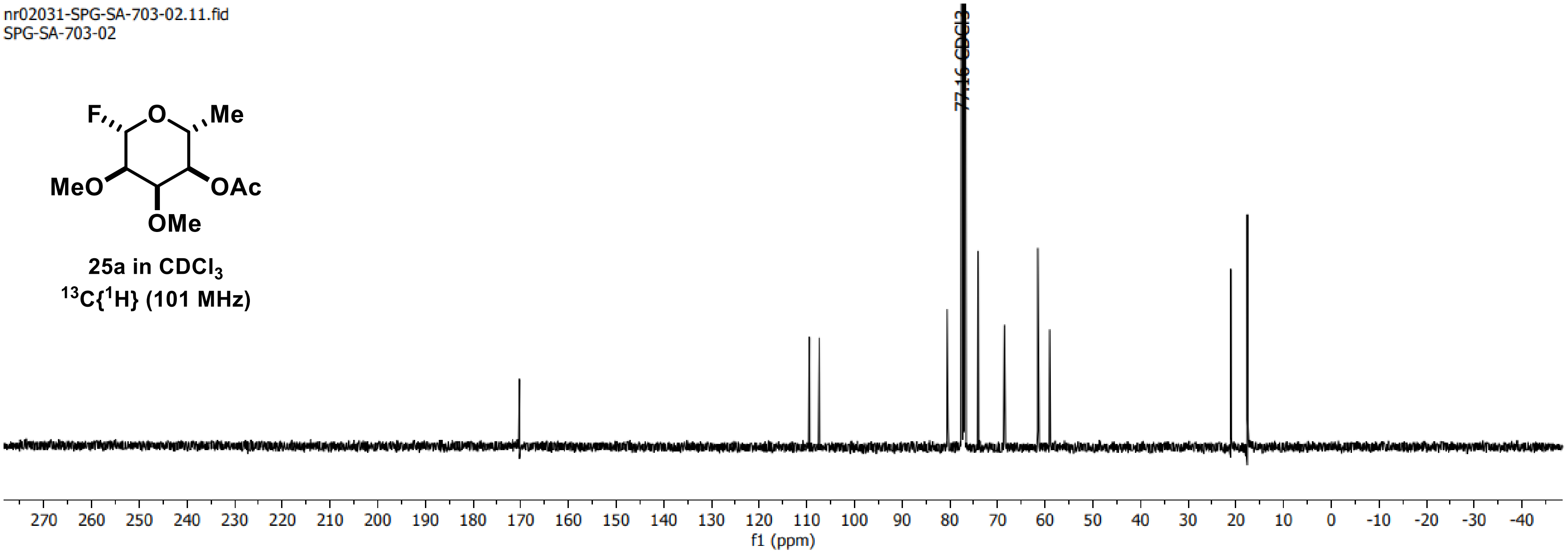

nr02031-SPG-SA-703-02.11.fid
SPG-SA-703-02
F
O
Me
MeO
OAc
OMe
25a in CDCl3
13C{1H} (101 MHz)
77.16 CDCl3
f1 (ppm)

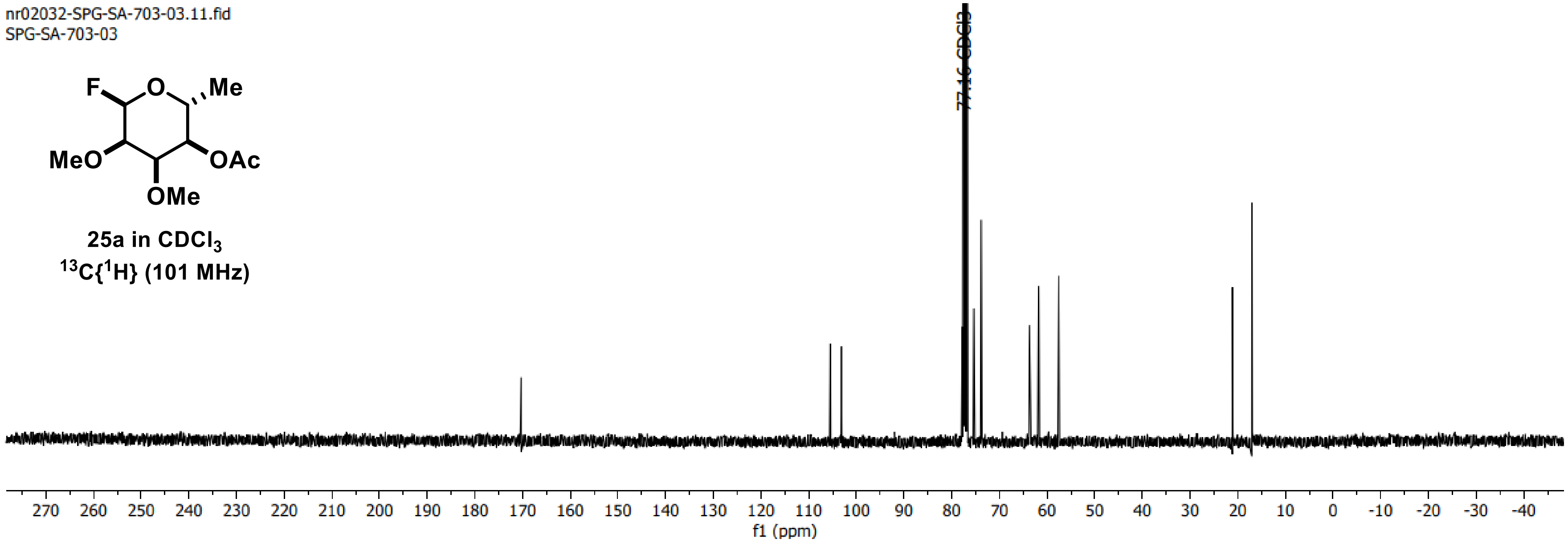

nr02032-SPG-SA-703-03.11.fid
SPG-SA-703-03
F
O
Me
MeO
OAc
OMe
25a in CDCl3
13C{1H} (101 MHz)
77.16 CDCl3
f1 (ppm)

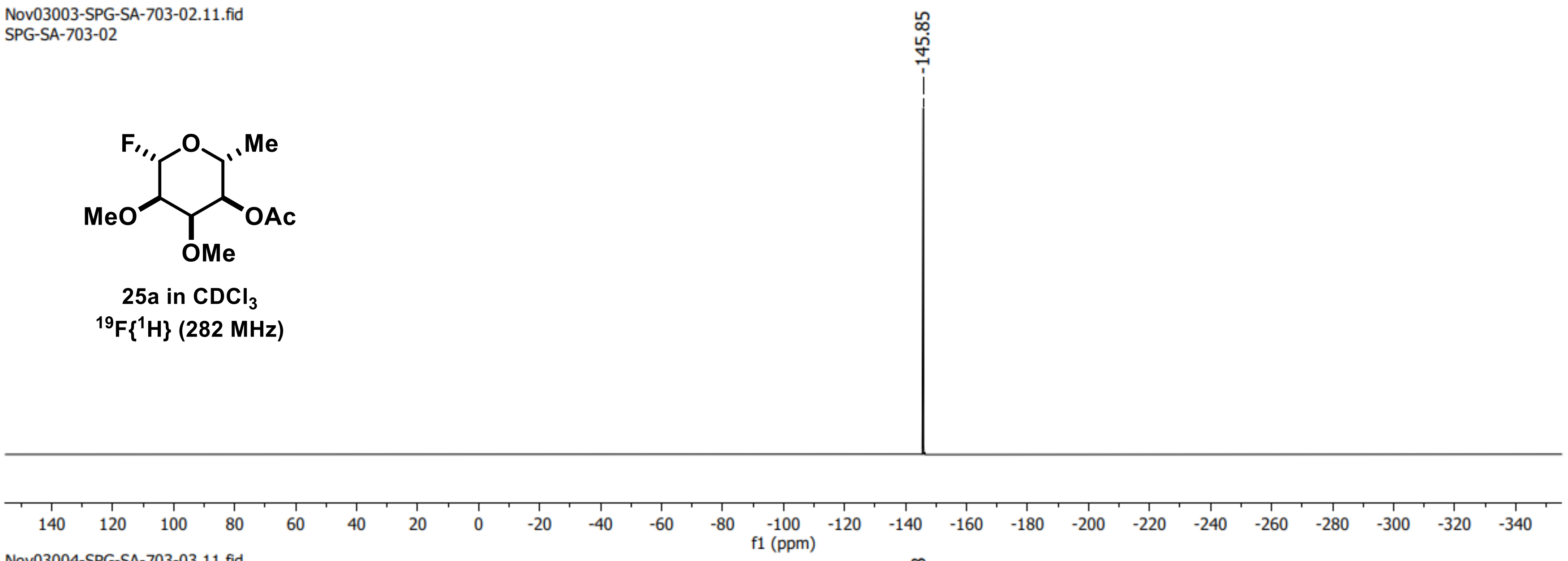
Nov03003-SPG-SA-703-02.11.fid
SPG-SA-703-02
-145.85
F
O
Me
MeO
OAc
OMe
25a in CDCl3
19F{1H} (282 MHz)
140 120 100 80 60 40 20 0 -20 -40 -60 -80 -100 -120 -140 -160 -180 -200 -220 -240 -260 -280 -300 -320 -340
f1 (ppm)

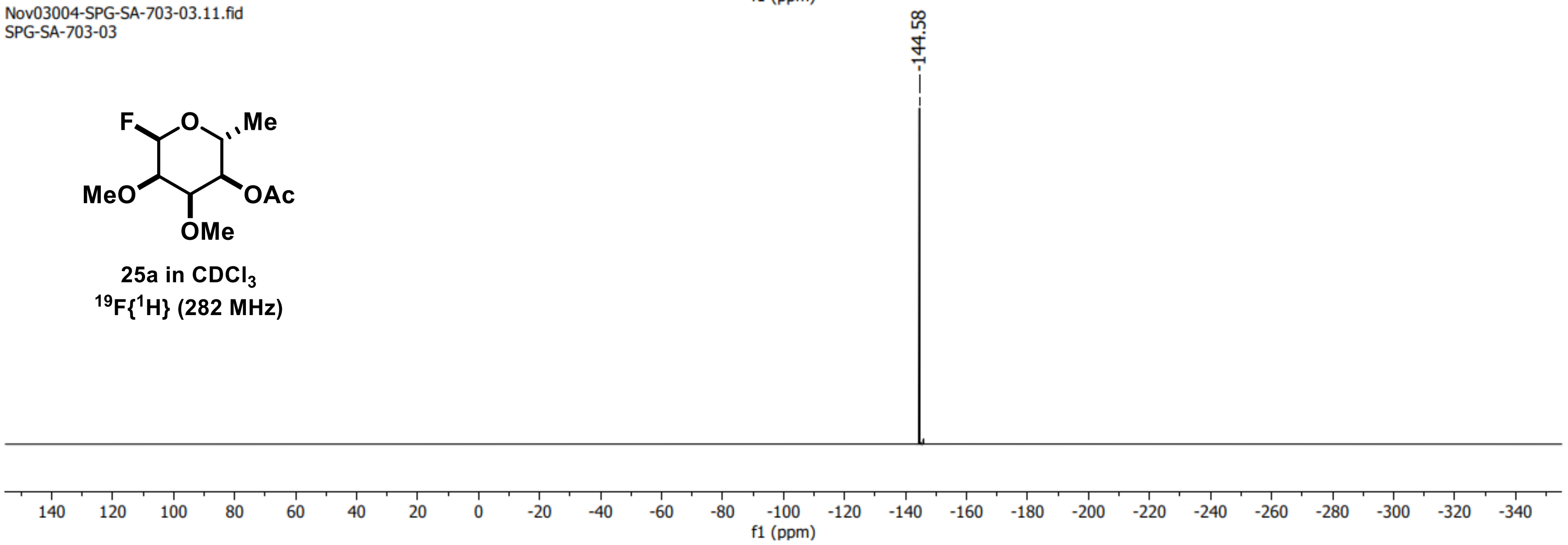
Nov03004-SPG-SA-703-03.11.fid
SPG-SA-703-03
-144.58
F
O
Me
MeO
OAc
OMe
25a in CDCl3
19F{1H} (282 MHz)
140 120 100 80 60 40 20 0 -20 -40 -60 -80 -100 -120 -140 -160 -180 -200 -220 -240 -260 -280 -300 -320 -340
f1 (ppm)

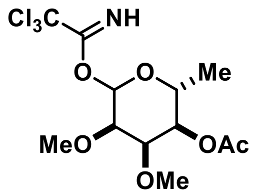

25b in $CDCl_3$
^{1}H (400 MHz)
$^{13}C\{^{1}H\}$ (101 MHz)

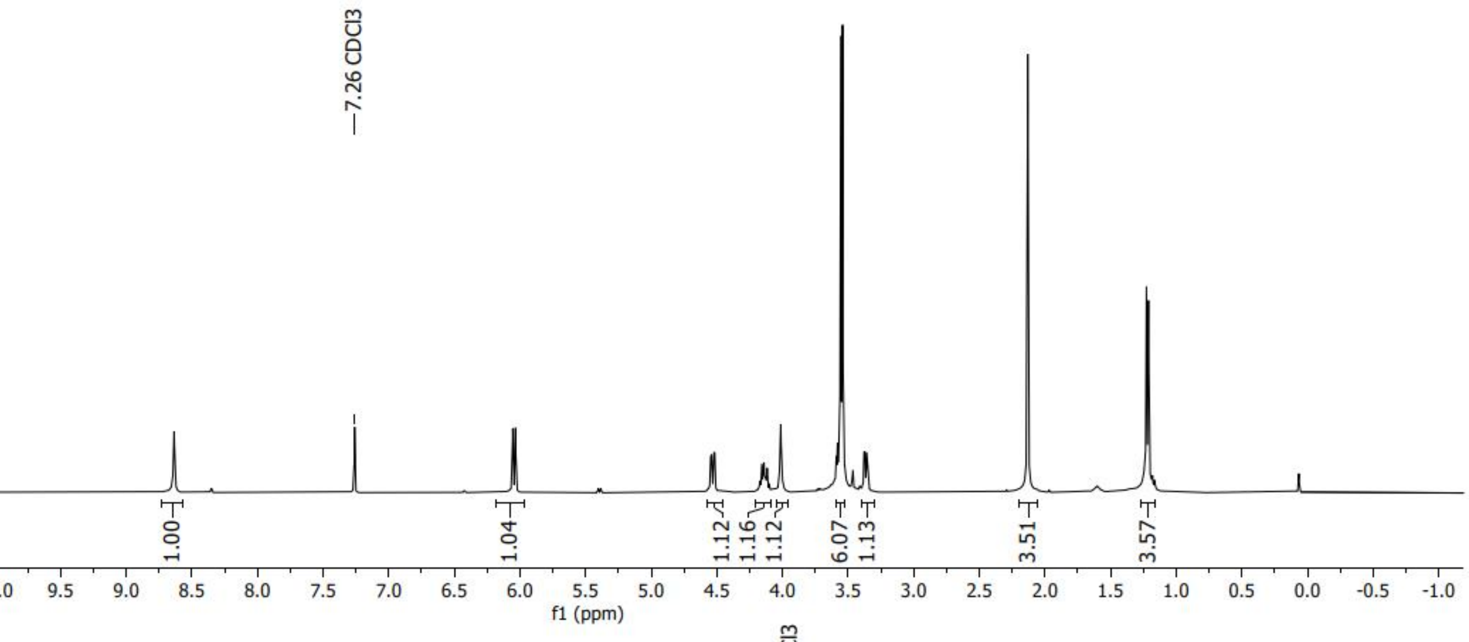

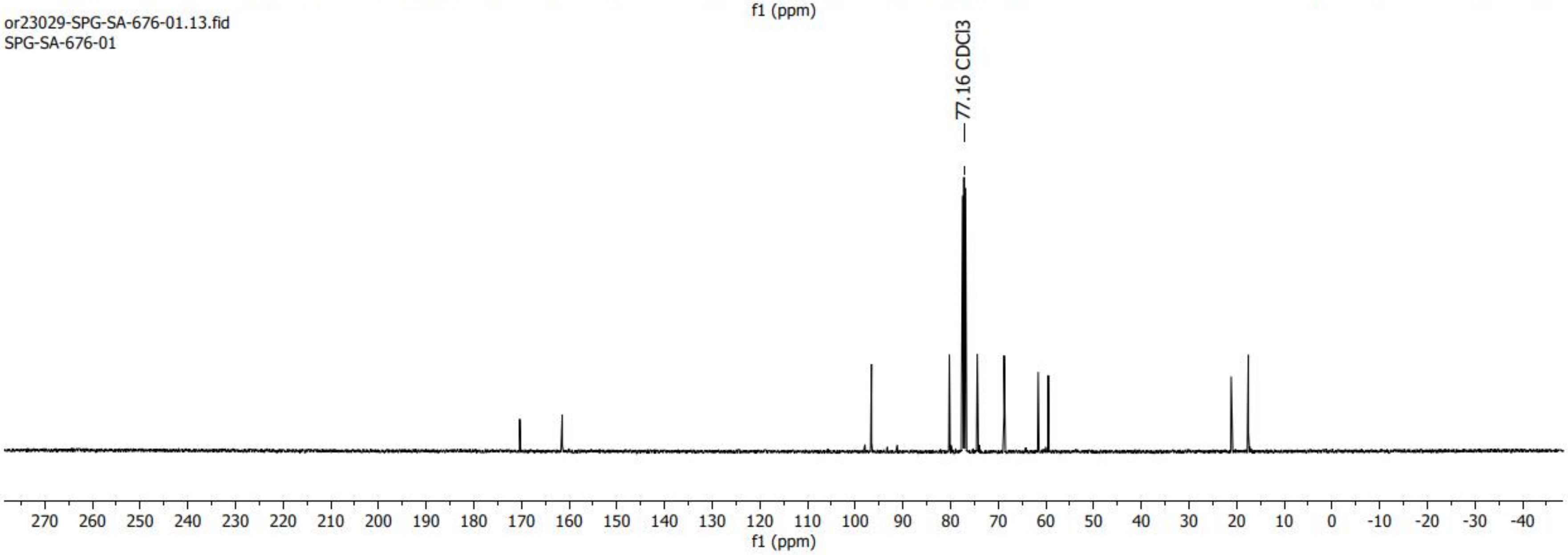

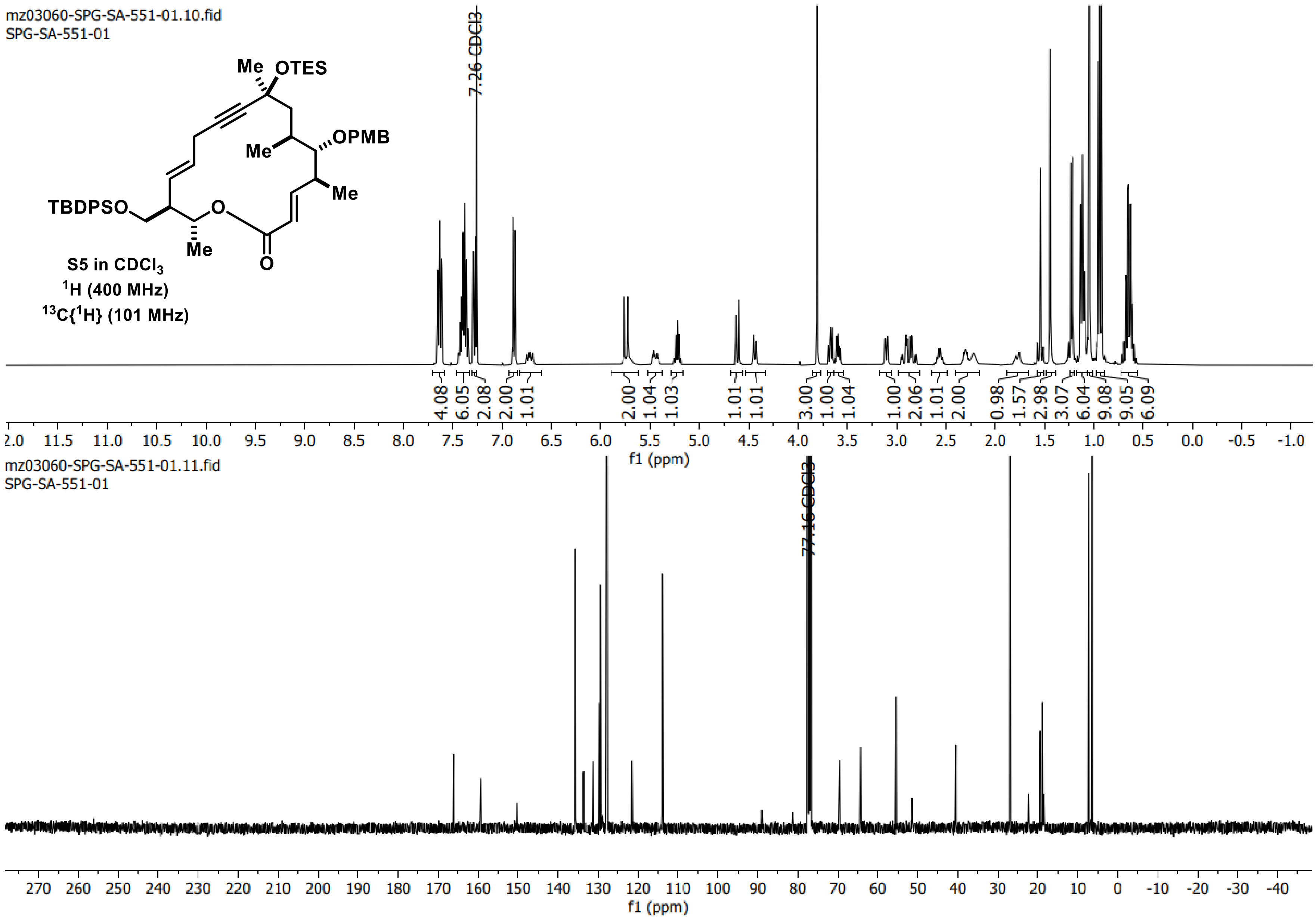
mz03060-SPG-SA-551-01.10.fid
SPG-SA-551-01
Me
OTES
OPMB
Me
Me
TBDPSO
O
Me
O
S5 in CDCl3
1H (400 MHz)
13C{1H} (101 MHz)
7.26 CDCl3
4.08
6.05
2.08
2.00
1.01
2.00
1.04
1.03
1.01
1.01
3.00
1.00
1.04
1.00
2.06
1.01
2.00
0.98
1.57
2.98
3.07
6.04
9.08
9.05
6.09
f1 (ppm)
mz03060-SPG-SA-551-01.11.fid
SPG-SA-551-01
77.16 CDCl3
f1 (ppm)

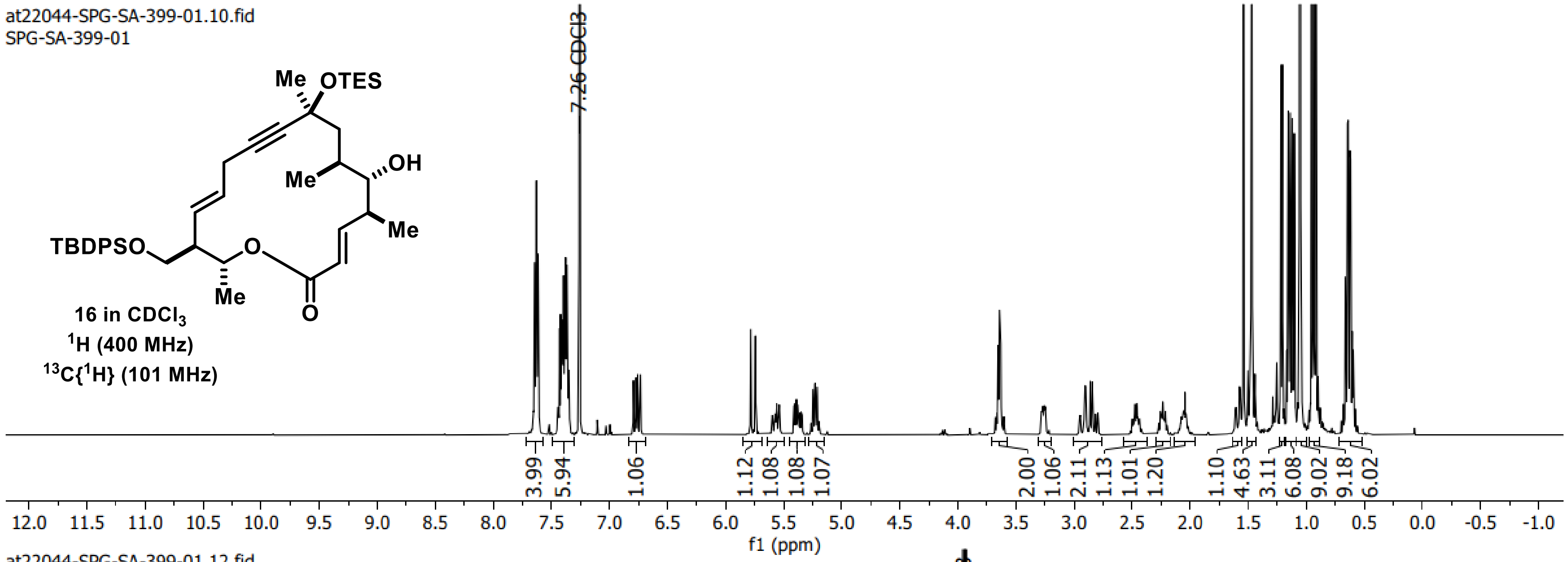
at22044-SPG-SA-399-01.10.fid
SPG-SA-399-01
Me OTES
OH
Me
Me
TBDPSO
O
Me
O
16 in CDCl3
1H (400 MHz)
13C{1H} (101 MHz)
7.26 CDCl3
3.99 5.94 1.06 1.12 1.08 1.08 1.07 2.00 1.06 2.11 1.13 1.01 1.20 1.10 4.63 3.11 6.08 9.02 9.18 6.02
f1 (ppm)

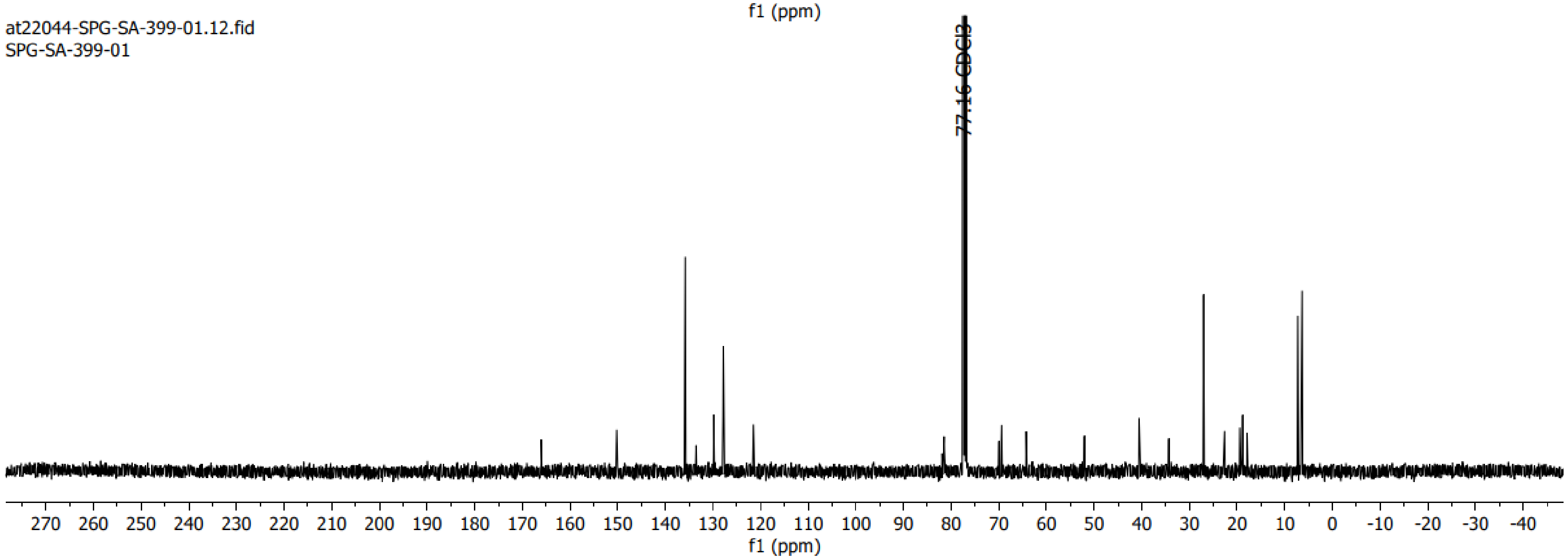
at22044-SPG-SA-399-01.12.fid
SPG-SA-399-01
77.16 CDCl3
f1 (ppm)

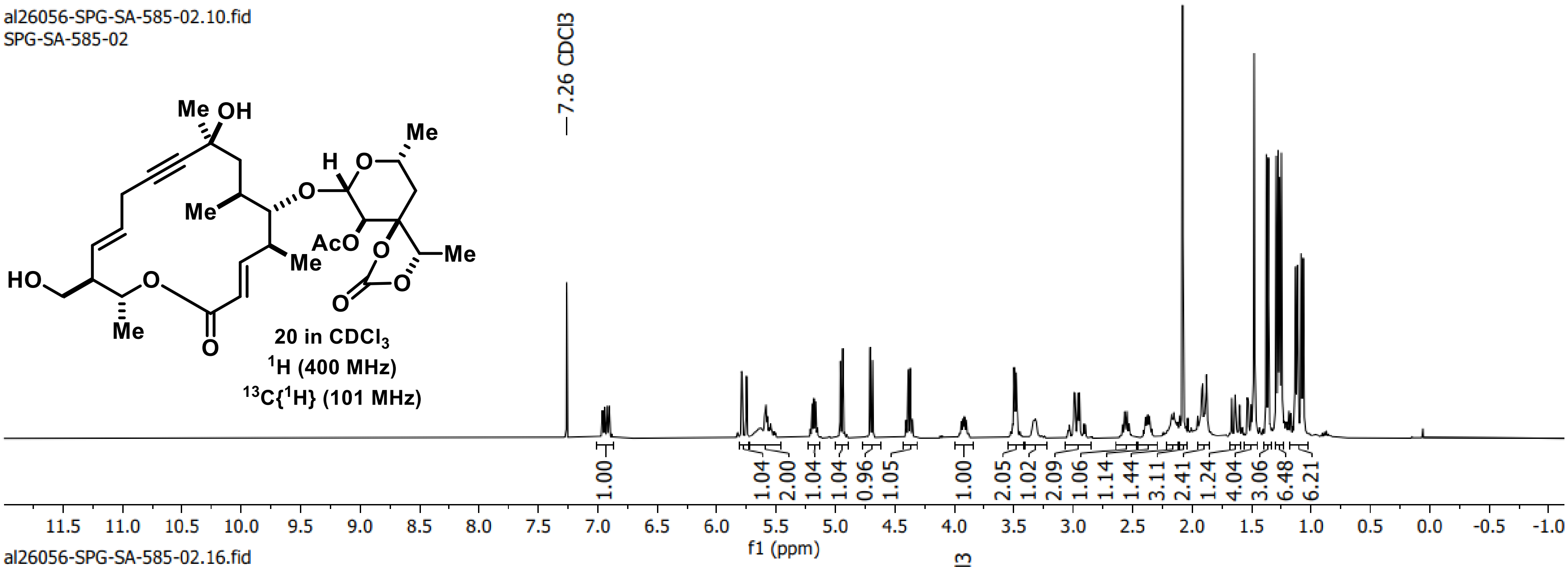
al26056-SPG-SA-585-02.10.fid
SPG-SA-585-02
7.26 CDCl3
Me OH
Me
H O
O
Me
AcO
O
Me
Me
HO
O
O
Me
O
O
20 in CDCl3
1H (400 MHz)
13C{1H} (101 MHz)
1.00
1.04
2.00
1.04
1.04
0.96
1.05
1.00
2.05
1.02
2.09
1.06
1.14
1.44
3.11
2.41
1.24
4.04
3.06
6.48
6.21
f1 (ppm)

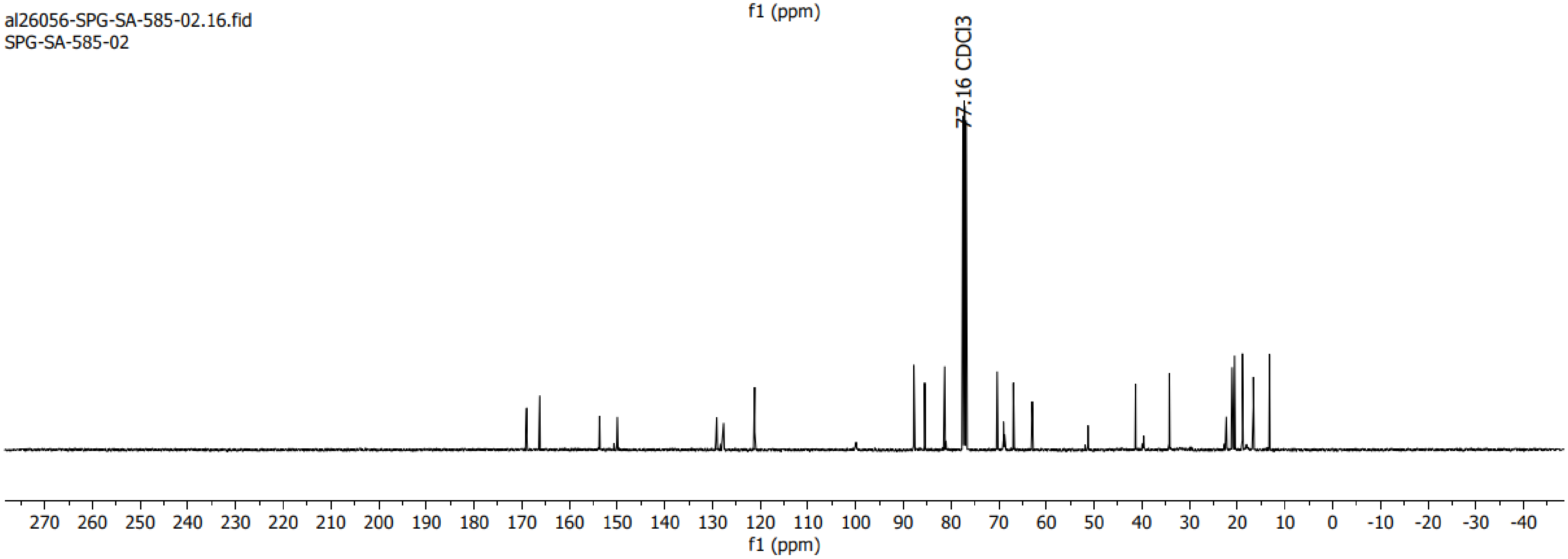
al26056-SPG-SA-585-02.16.fid
SPG-SA-585-02
77.16 CDCl3
f1 (ppm)

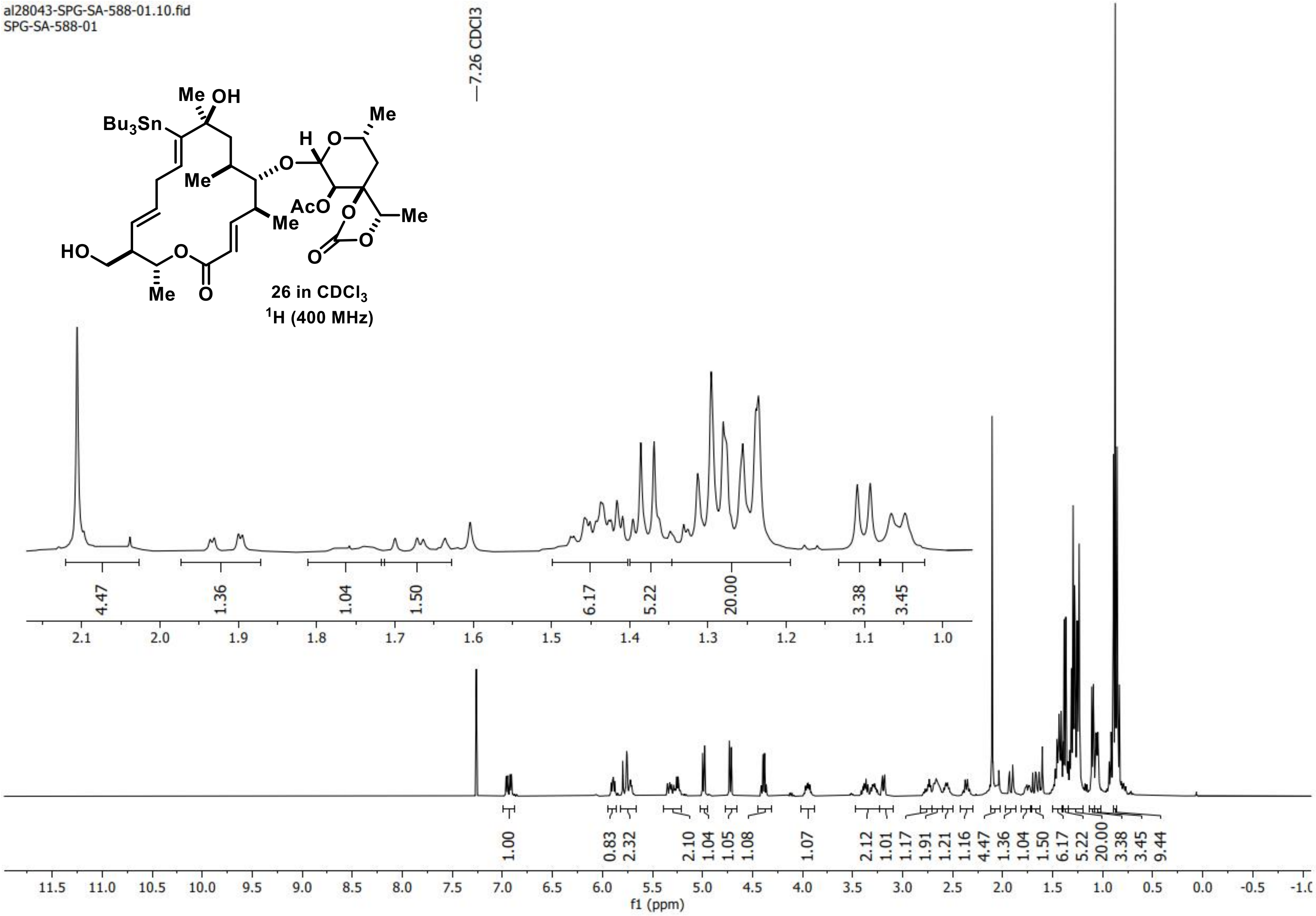
al28043-SPG-SA-588-01.10.fid
SPG-SA-588-01
—7.26 CDCl3
26 in CDCl3
1H (400 MHz)
4.47
1.36
1.04
1.50
6.17
5.22
20.00
3.38
3.45
1.00
0.83
2.32
2.10
1.04
1.05
1.08
1.07
2.12
1.01
1.17
1.91
1.21
1.16
9.44
f1 (ppm)

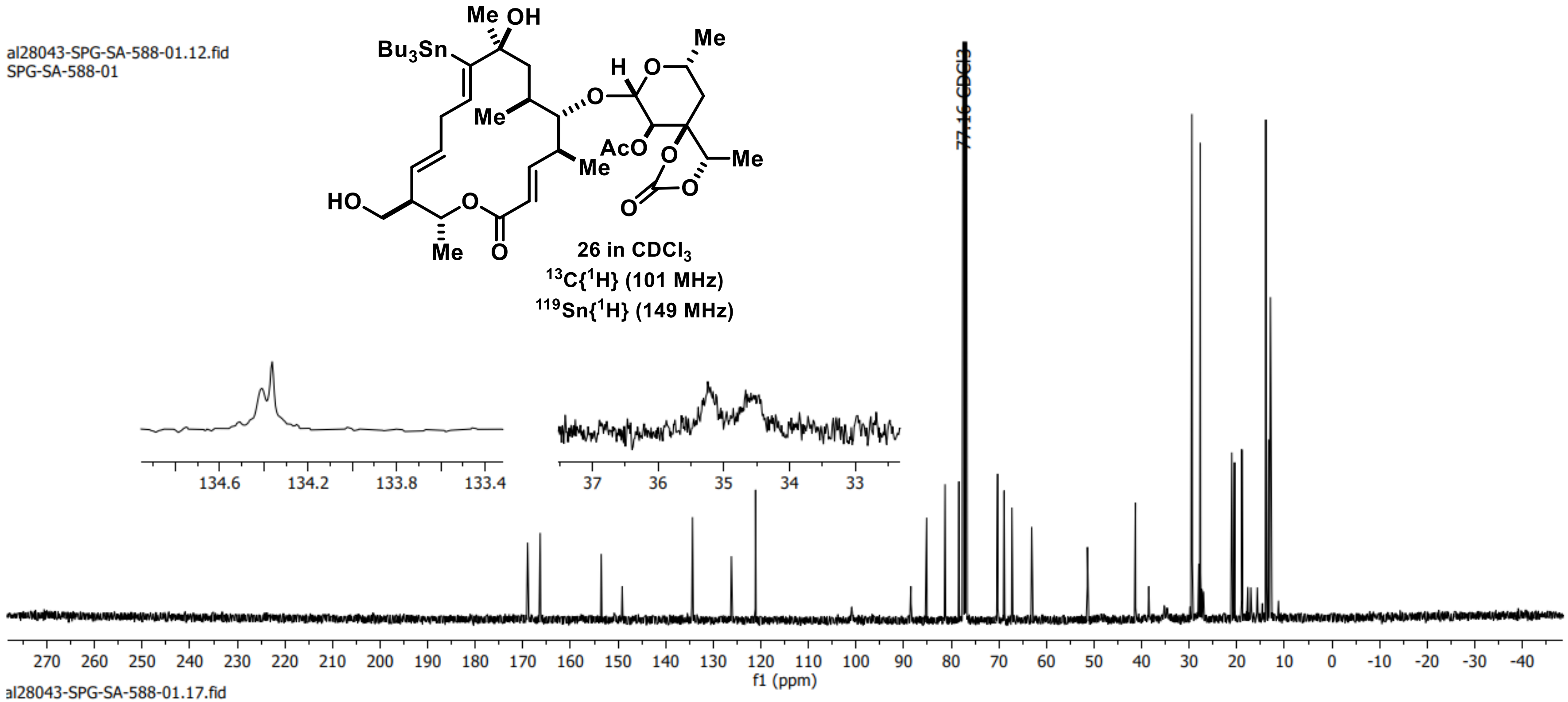

al28043-SPG-SA-588-01.17.fid
SPG-SA-588-01

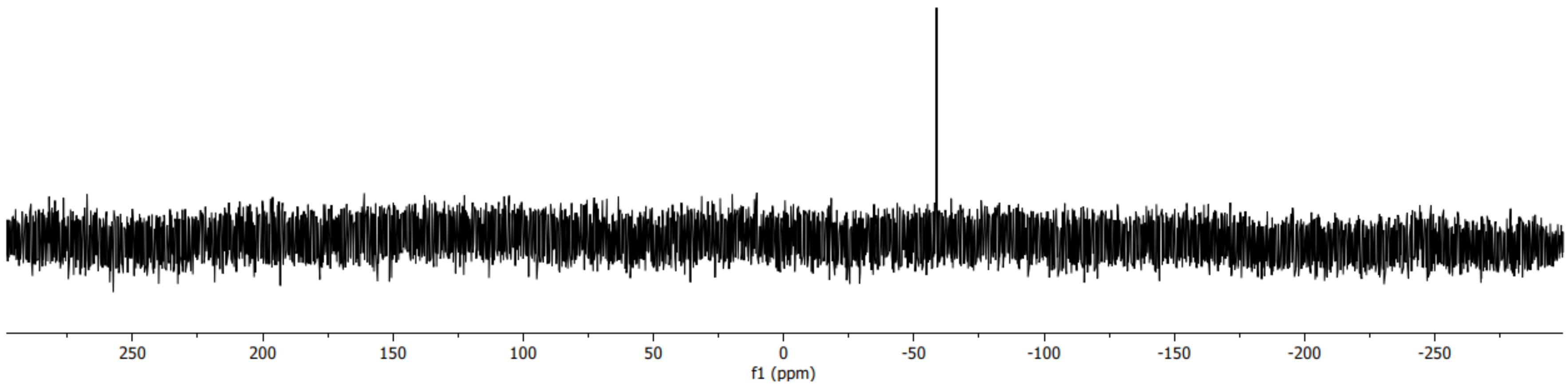

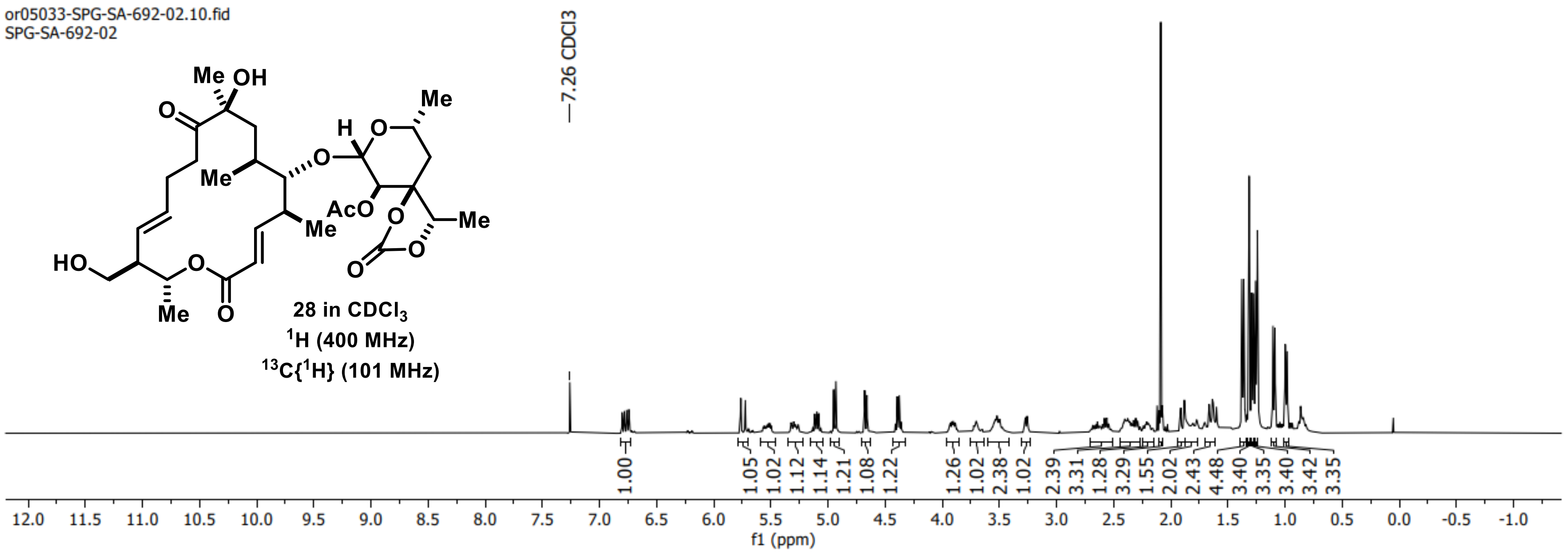
or05033-SPG-SA-692-02.10.fid
SPG-SA-692-02
Me OH
O
Me
H O
Me
O
AcO
Me
O
Me
HO
O
O
O
O
Me
O
28 in $CDCl_3$
^1H (400 MHz)
$^{13}C\{^1H\}$ (101 MHz)
7.26 CDCl3
1.00
1.05
1.02
1.12
1.14
1.21
1.08
1.22
1.26
1.02
2.38
1.02
2.39
3.31
1.28
3.29
1.55
2.02
2.43
4.48
3.40
3.35
3.40
3.42
3.35
f1 (ppm)

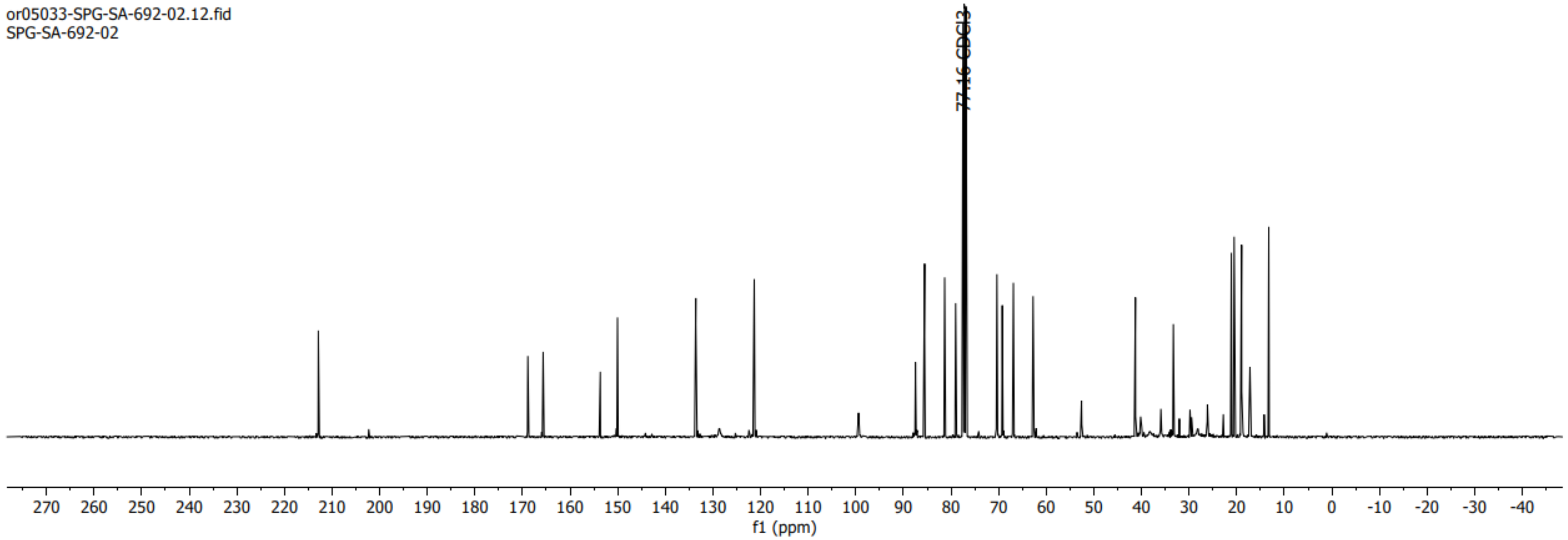
or05033-SPG-SA-692-02.12.fid
SPG-SA-692-02
77.16 CDCl3
f1 (ppm)

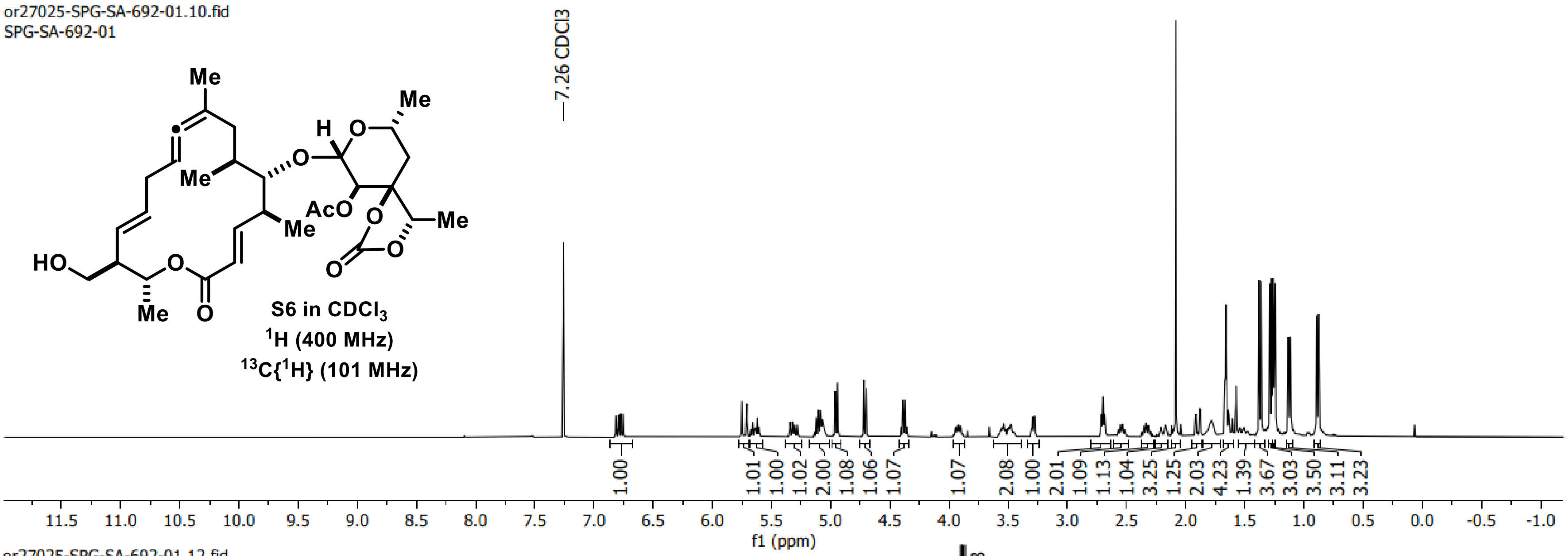

or27025-SPG-SA-692-01.10.fid
SPG-SA-692-01
7.26 CDCl3
Me
Me
H
O
Me
O
Me
AcO
O
Me
Me
O
HO
O
O
Me
O
S6 in CDCl3
1H (400 MHz)
13C{1H} (101 MHz)
1.00
1.01
1.00
1.02
2.00
1.08
1.06
1.07
1.07
2.08
1.00
2.01
1.09
1.13
1.04
3.25
1.25
2.03
4.23
1.39
3.67
3.03
3.50
3.11
3.23
11.5 11.0 10.5 10.0 9.5 9.0 8.5 8.0 7.5 7.0 6.5 6.0 5.5 5.0 4.5 4.0 3.5 3.0 2.5 2.0 1.5 1.0 0.5 0.0 -0.5 -1.0
f1 (ppm)

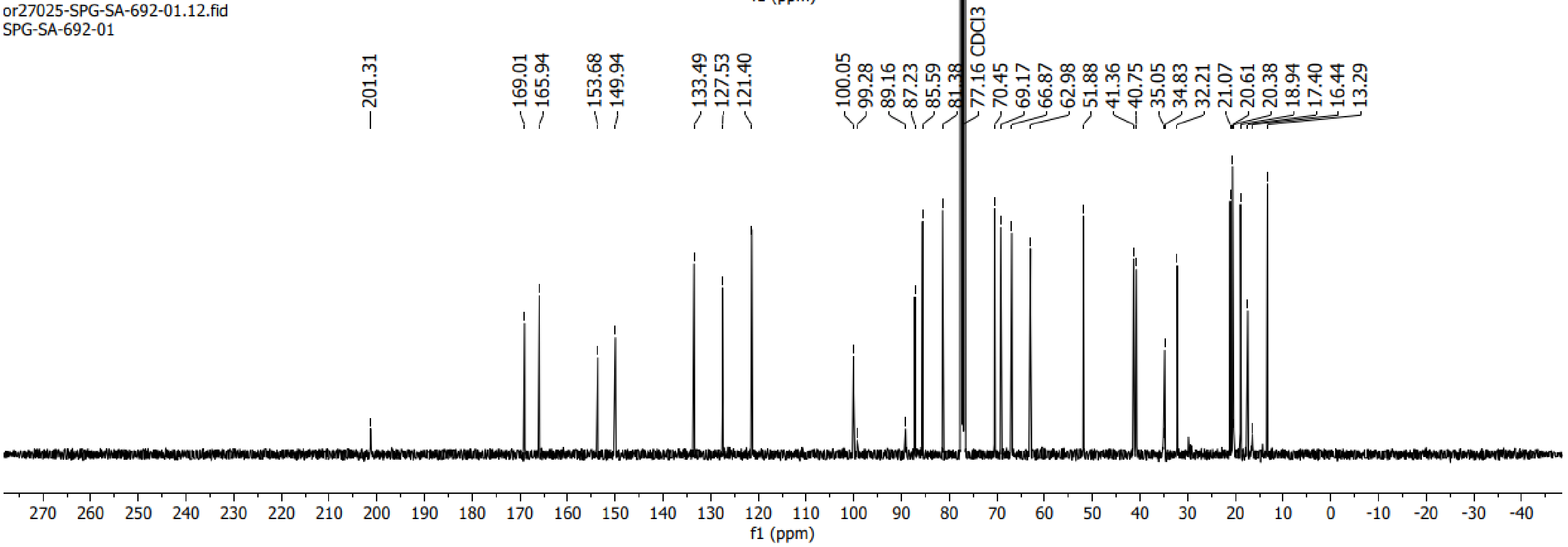

or27025-SPG-SA-692-01.12.fid
SPG-SA-692-01
201.31
169.01
165.94
153.68
149.94
133.49
127.53
121.40
100.05
99.28
89.16
87.23
85.59
81.38
77.16 CDCl3
70.45
69.17
66.87
62.98
51.88
41.36
40.75
35.05
34.83
32.21
21.07
20.61
20.38
18.94
17.40
16.44
13.29
270 260 250 240 230 220 210 200 190 180 170 160 150 140 130 120 110 100 90 80 70 60 50 40 30 20 10 0 -10 -20 -30 -40
f1 (ppm)

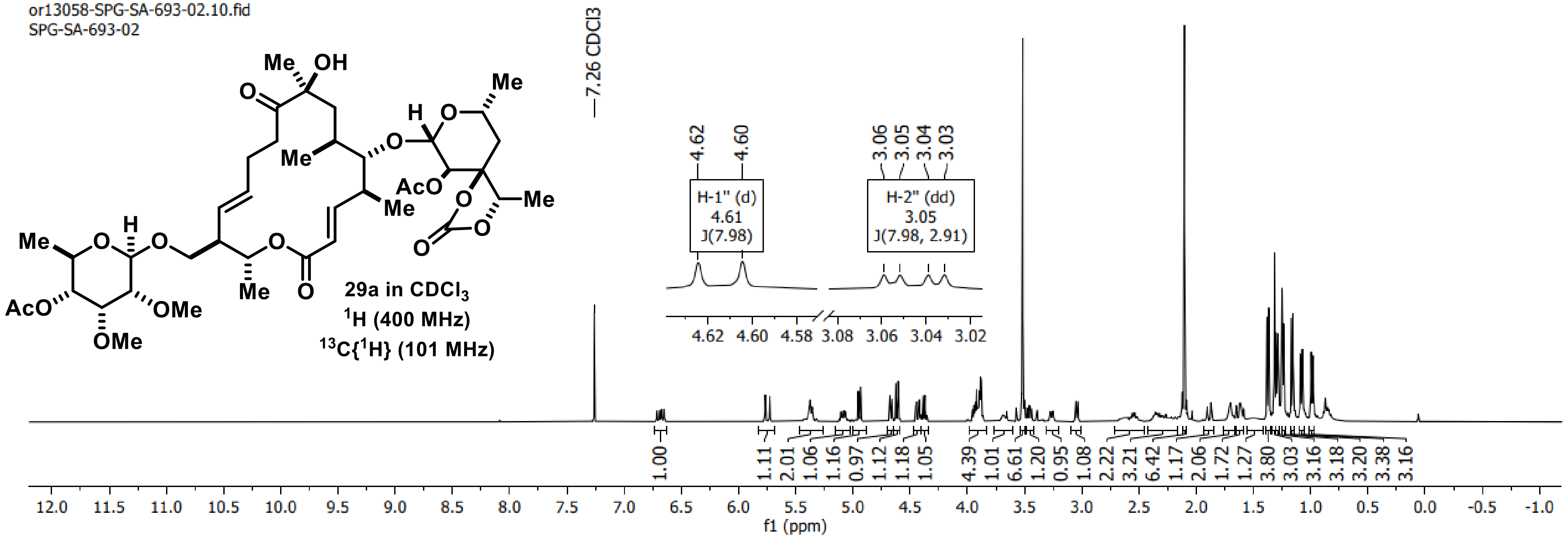
or13058-SPG-SA-693-02.10.fid
SPG-SA-693-02
29a in CDCl3
1H (400 MHz)
13C{1H} (101 MHz)
7.26 CDCl3
4.62
4.60
H-1'' (d)
4.61
J(7.98)
3.06
3.05
3.04
3.03
H-2'' (dd)
3.05
J(7.98, 2.91)
1.00
1.11
2.01
1.06
1.16
0.97
1.12
1.18
1.05
4.39
1.01
6.61
1.20
0.95
1.08
2.22
3.21
6.42
1.17
2.06
1.72
1.27
3.80
3.03
3.16
3.18
3.20
3.38
3.16
f1 (ppm)

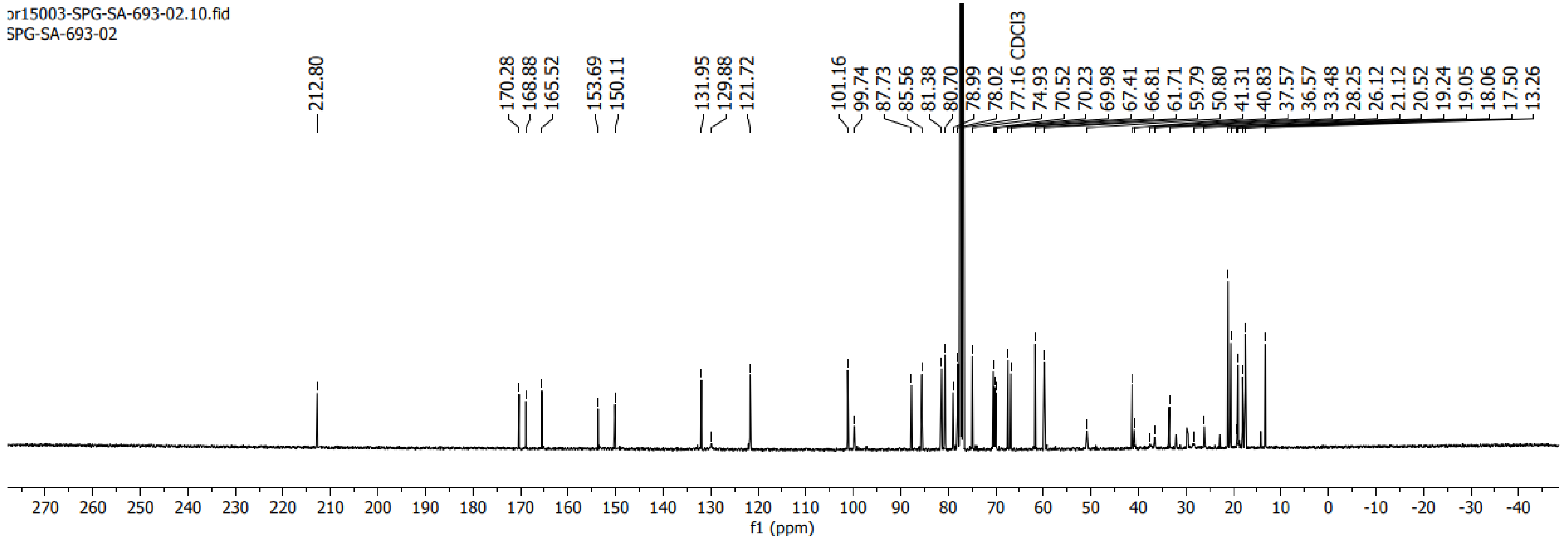
or15003-SPG-SA-693-02.10.fid
SPG-SA-693-02
212.80
170.28
168.88
165.52
153.69
150.11
131.95
129.88
121.72
101.16
99.74
87.73
85.56
81.38
80.70
78.99
78.02
77.16 CDCl3
74.93
70.52
70.23
69.98
67.41
66.81
61.71
59.79
50.80
41.31
40.83
37.57
36.57
33.48
28.25
26.12
21.12
20.52
19.24
19.05
18.06
17.50
13.26
f1 (ppm)

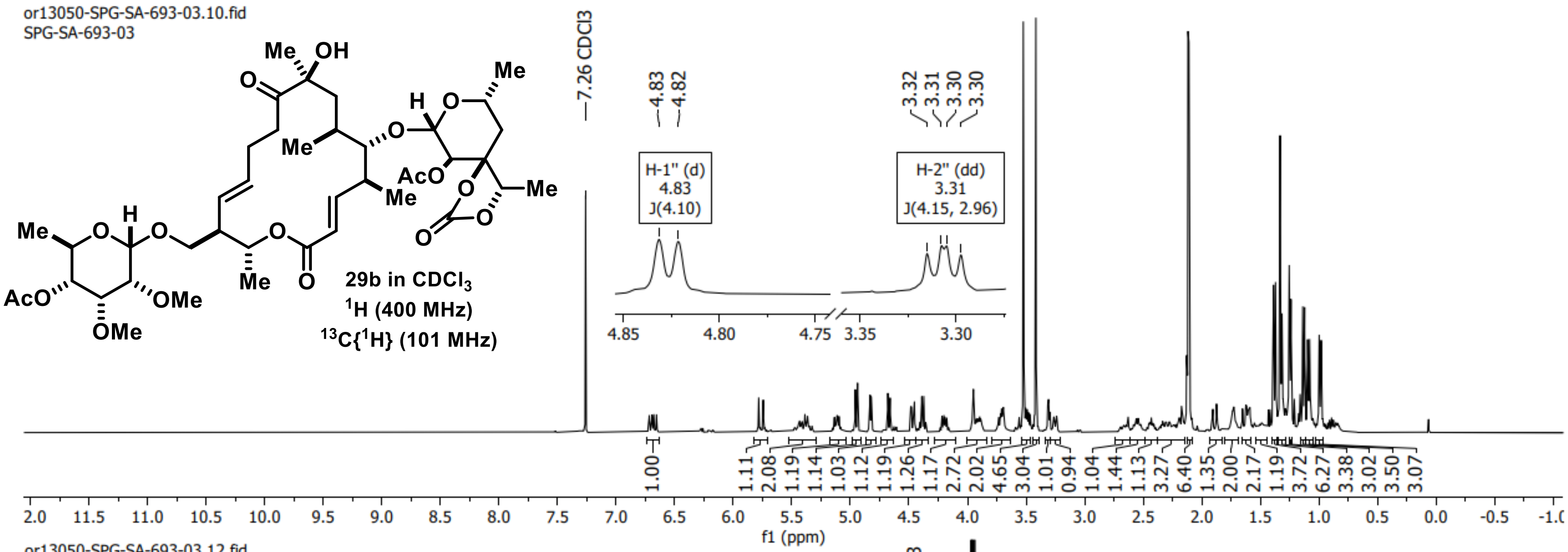
or13050-SPG-SA-693-03.10.fid
SPG-SA-693-03
29b in CDCl3
1H (400 MHz)
13C{1H} (101 MHz)
7.26 CDCl3
4.83
4.82
3.32
3.31
3.30
3.30
H-1" (d)
4.83
J(4.10)
H-2" (dd)
3.31
J(4.15, 2.96)
1.00
1.11
2.08
1.19
1.14
1.03
1.12
1.19
1.26
1.17
2.72
2.02
4.65
3.04
1.01
0.94
1.04
1.44
1.13
3.27
6.40
1.35
2.00
2.17
1.19
3.72
6.27
3.38
3.02
3.50
3.07
f1 (ppm)

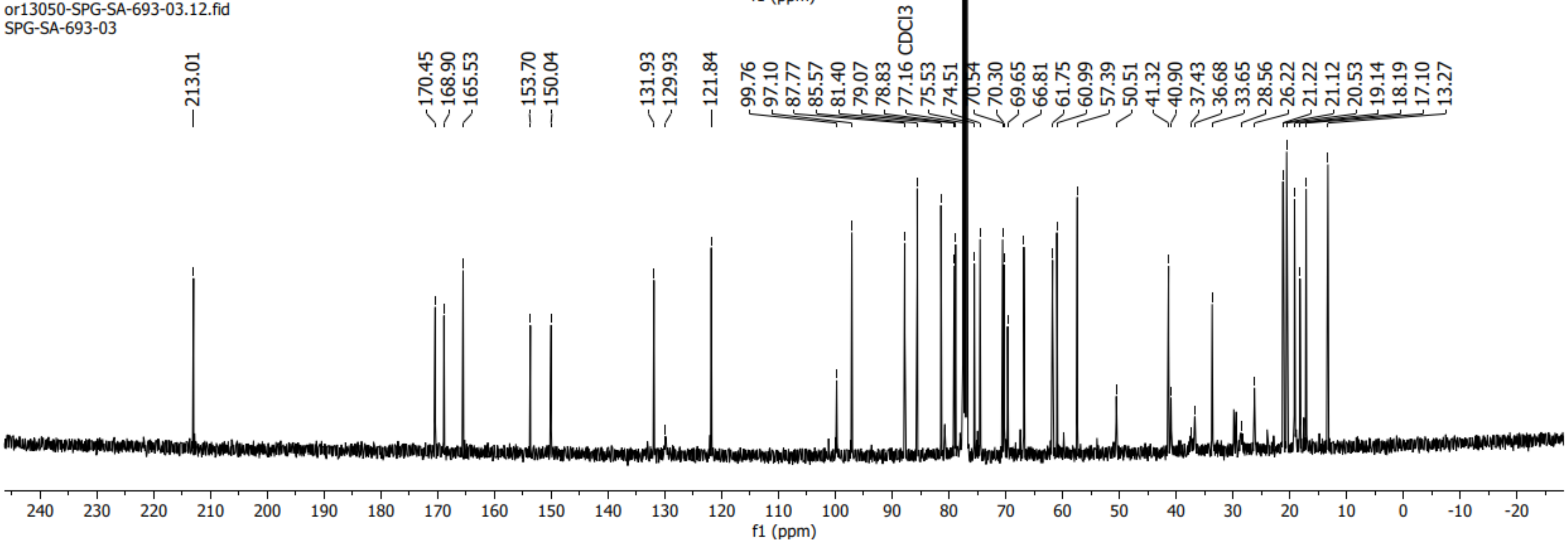
or13050-SPG-SA-693-03.12.fid
SPG-SA-693-03
213.01
170.45
168.90
165.53
153.70
150.04
131.93
129.93
121.84
99.76
97.10
87.77
85.57
81.40
79.07
78.83
77.16 CDCl3
75.53
74.51
70.54
70.30
69.65
66.81
61.75
60.99
57.39
50.51
41.32
40.90
37.43
36.68
33.65
28.56
26.22
21.22
21.12
20.53
19.14
18.19
17.10
13.27
f1 (ppm)

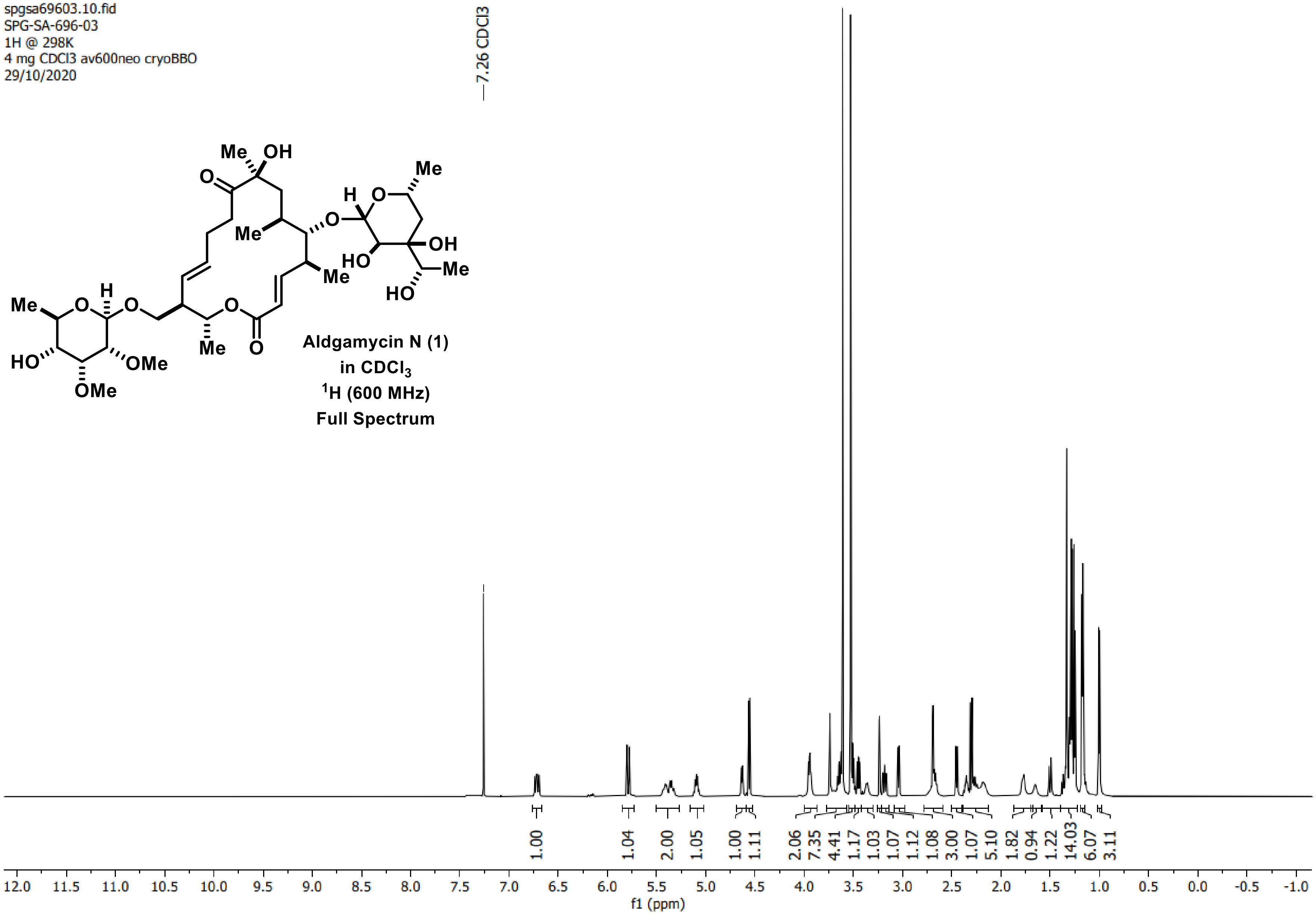

spgsa69603.10.fid
SPG-SA-696-03
1H @ 298K
4 mg CDCl3 av600neo cryoBBO
29/10/2020
7.26 CDCl3
Aldgamycin N (1)
in CDCl3
1H (600 MHz)
Full Spectrum
1.00
1.04
2.00
1.05
1.00
1.11
2.06
7.35
4.41
1.17
1.03
1.07
1.12
1.08
3.00
1.07
5.10
1.82
0.94
1.22
14.03
6.07
3.11
f1 (ppm)

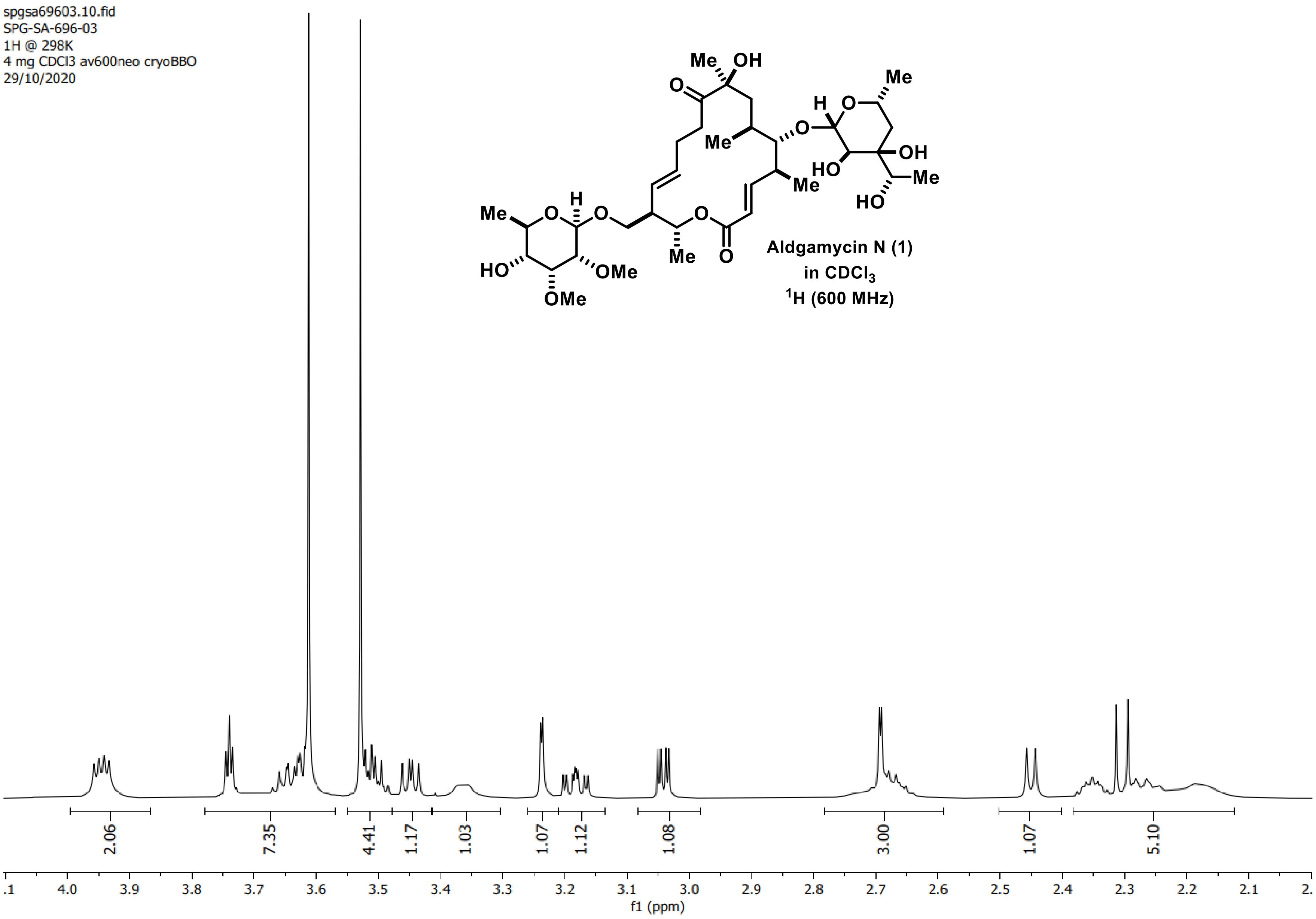
spgsa69603.10.fid
SPG-SA-696-03
1H @ 298K
4 mg CDCl3 av600neo cryoBBO
29/10/2020
Aldgamycin N (1)
in CDCl3
1H (600 MHz)
2.06
7.35
4.41
1.17
1.03
1.07
1.12
1.08
3.00
1.07
5.10
f1 (ppm)

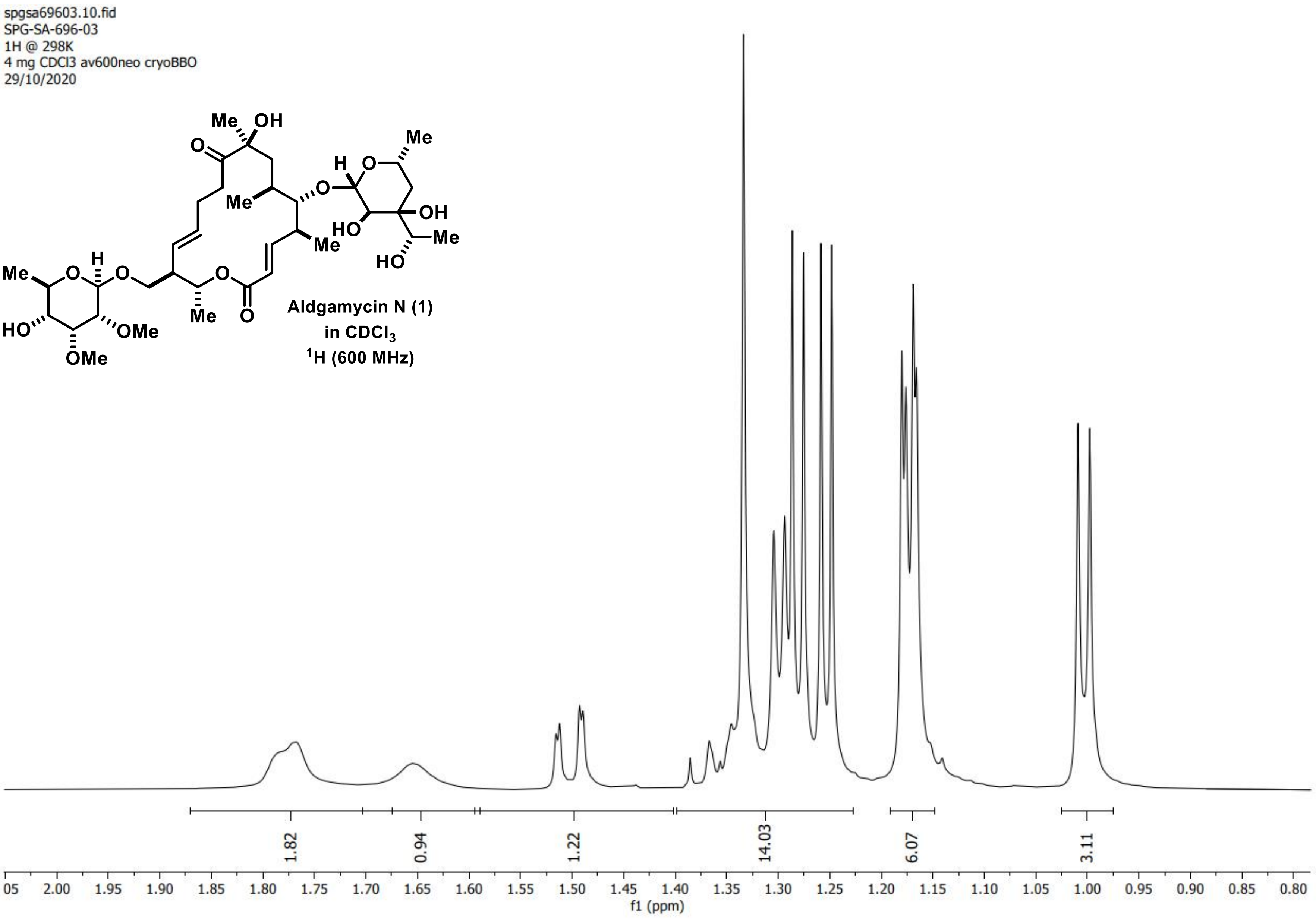
spgsa69603.10.fid
SPG-SA-696-03
1H @ 298K
4 mg CDCl3 av600neo cryoBBO
29/10/2020
Aldgamycin N (1)
in CDCl3
1H (600 MHz)
1.82
0.94
1.22
14.03
6.07
3.11
05 2.00 1.95 1.90 1.85 1.80 1.75 1.70 1.65 1.60 1.55 1.50 1.45 1.40 1.35 1.30 1.25 1.20 1.15 1.10 1.05 1.00 0.95 0.90 0.85 0.80
f1 (ppm)

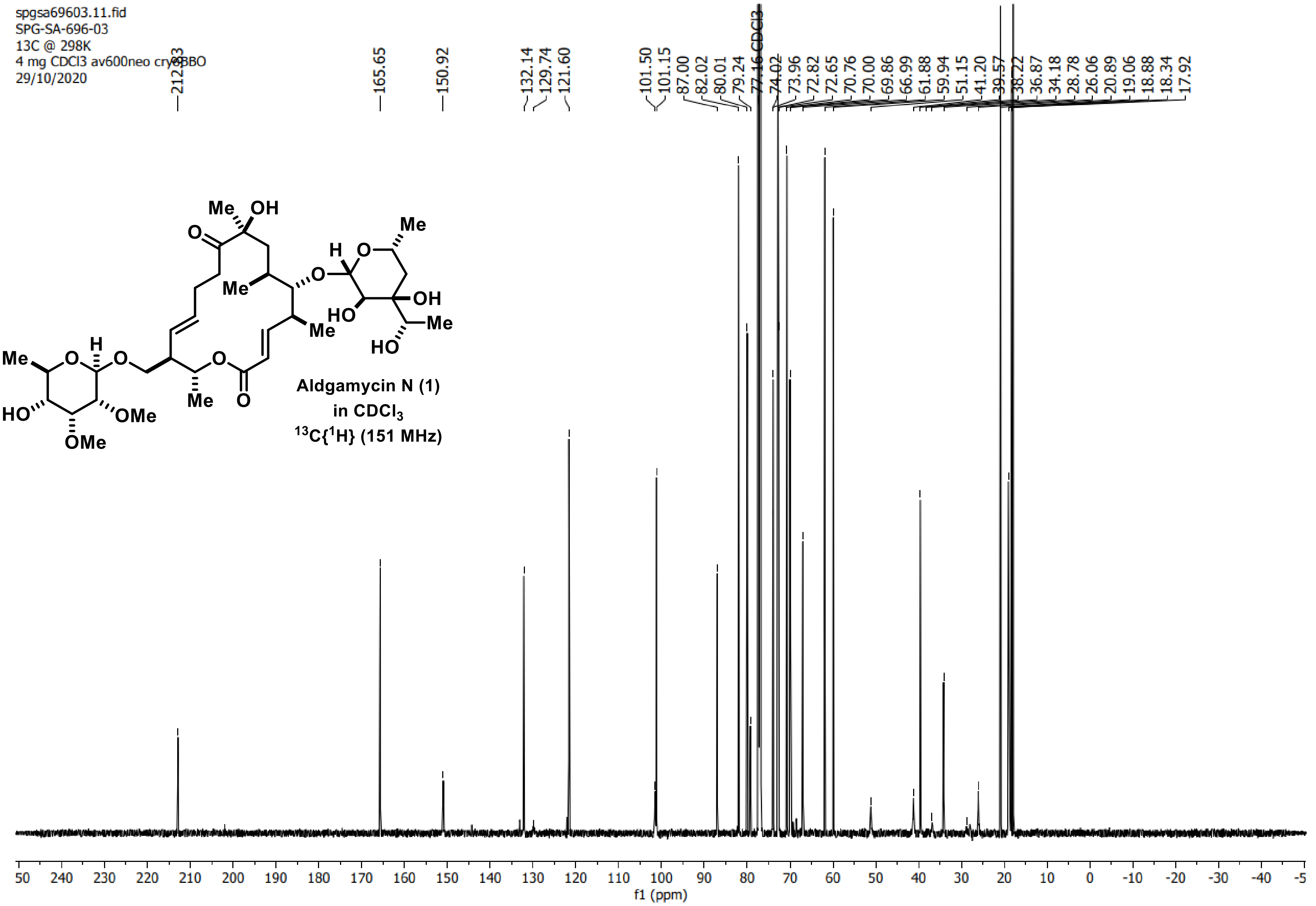
spgsa69603.11.fid
SPG-SA-696-03
13C @ 298K
4 mg CDCl3 av600neo cryoBBO
29/10/2020
212.83
165.65
150.92
132.14
129.74
121.60
101.50
101.15
87.00
82.02
80.01
79.24
77.16 CDCl3
74.02
73.96
72.82
72.65
70.76
70.00
69.86
66.99
61.88
59.94
51.15
41.20
39.57
38.22
36.87
34.18
28.78
26.06
20.89
19.06
18.88
18.34
17.92
Aldgamycin N (1)
in CDCl3
13C{1H} (151 MHz)
f1 (ppm)

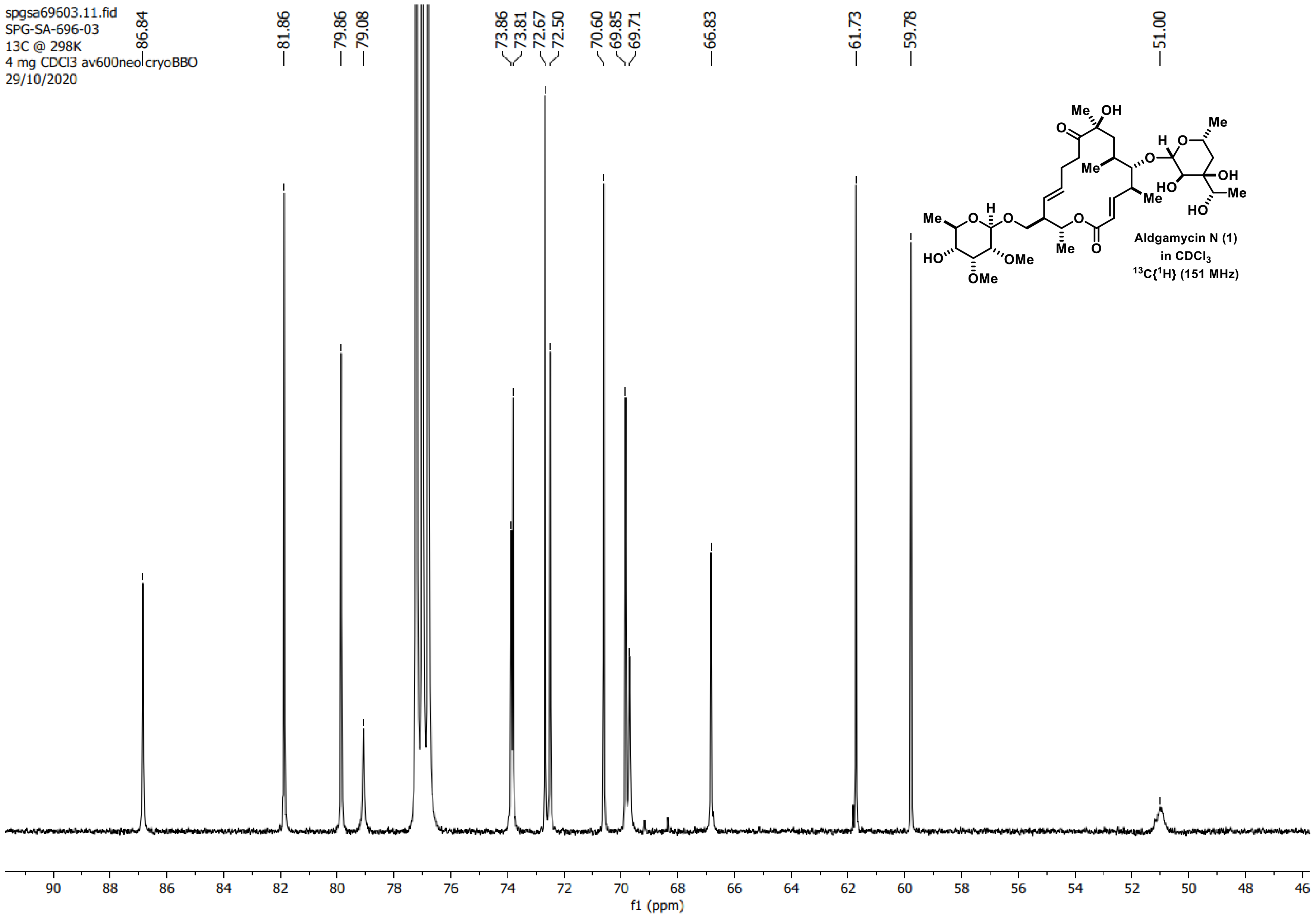

spgsa69603.11.fid
SPG-SA-696-03
13C @ 298K
4 mg CDCl3 av600neo cryoBBO
29/10/2020
86.84
81.86
79.86
79.08
73.86
73.81
72.67
72.50
70.60
69.85
69.71
66.83
61.73
59.78
51.00
Aldgamycin N (1)
in CDCl3
13C{1H} (151 MHz)
Me
OH
O
H
HO
Me
OMe
f1 (ppm)
90
88
86
84
82
80
78
76
74
72
70
68
66
64
62
60
58
56
54
52
50
48
46

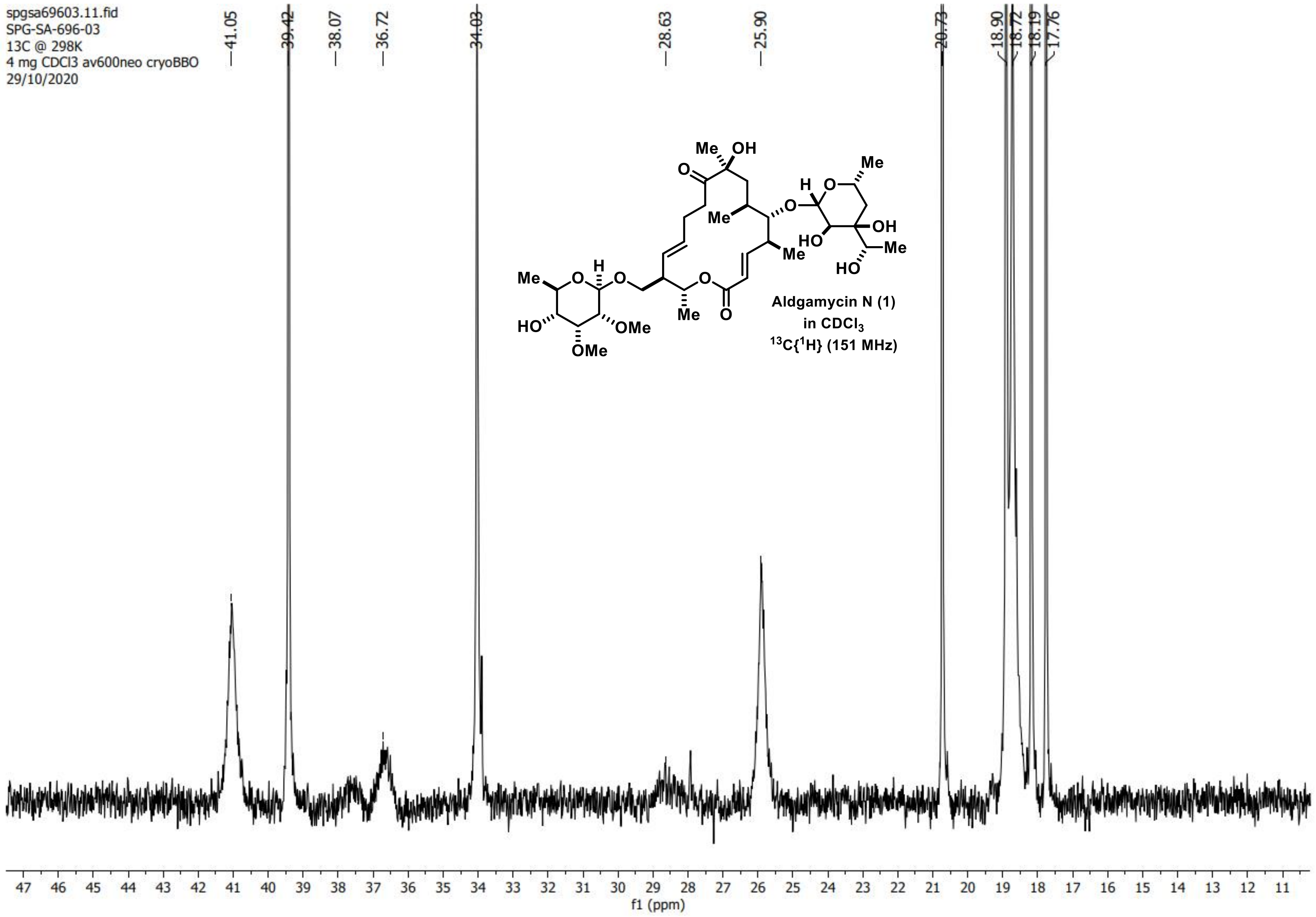

Aldgamycin N (1)
in $CDCl_3$
$^{13}C\{^1H\}$ (151 MHz)

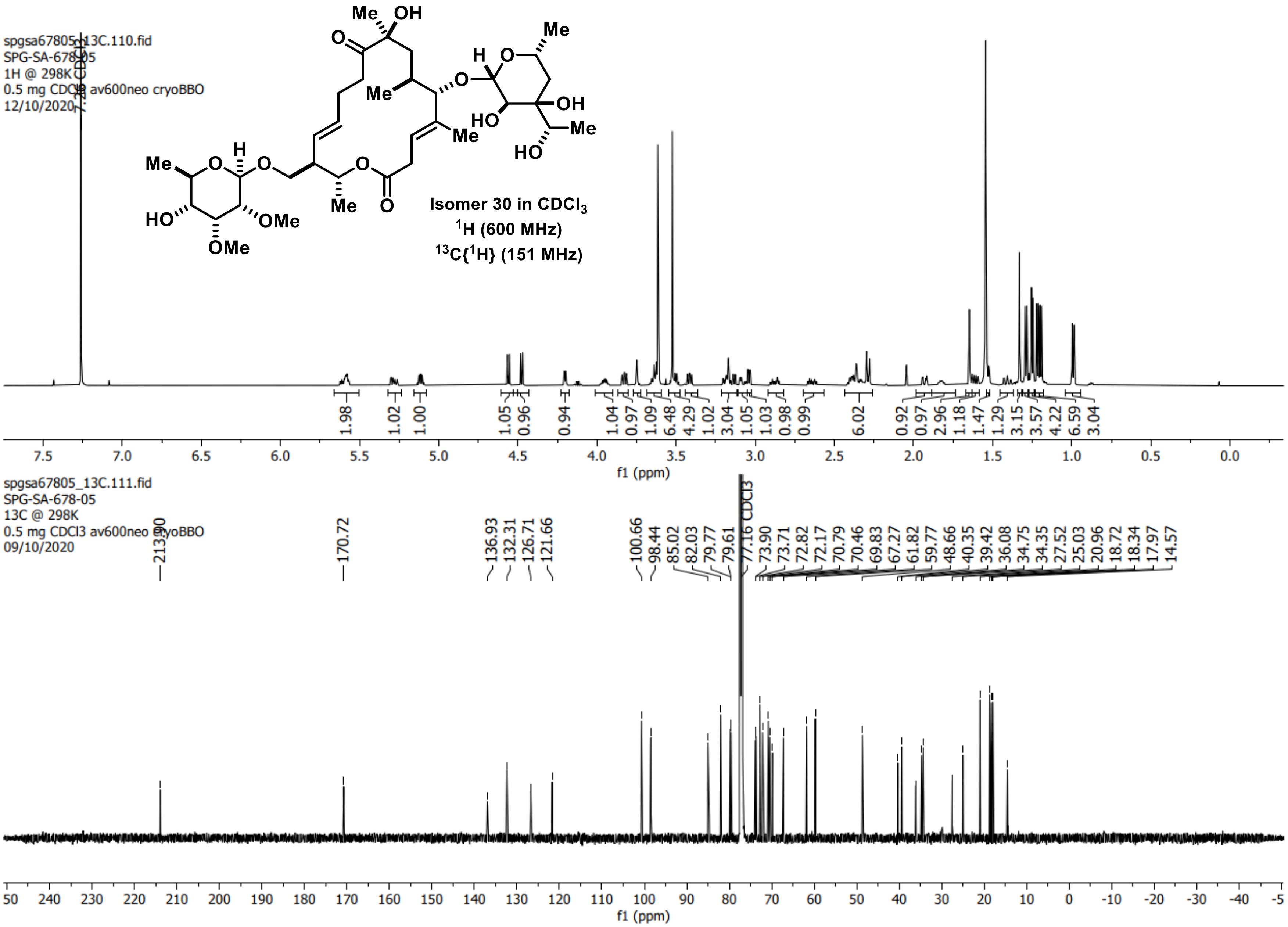
spgsa67805_13C.110.fid
SPG-SA-678-05
1H @ 298K
0.5 mg CDCl3 av600neo cryoBBO
12/10/2020
Isomer 30 in CDCl3
1H (600 MHz)
13C{1H} (151 MHz)
1.98
1.02
1.00
1.05
0.96
0.94
1.04
0.97
1.09
6.48
4.29
1.02
3.04
1.05
1.03
0.98
0.99
6.02
0.92
0.97
2.96
1.18
1.47
1.29
3.15
3.57
4.22
6.59
3.04
f1 (ppm)
spgsa67805_13C.111.fid
SPG-SA-678-05
13C @ 298K
0.5 mg CDCl3 av600neo cryoBBO
09/10/2020
213.90
170.72
136.93
132.31
126.71
121.66
100.66
98.44
85.02
82.03
79.77
79.61
77.16 CDCl3
73.90
73.71
72.82
72.17
70.79
70.46
69.83
67.27
61.82
59.77
48.66
40.35
39.42
36.08
34.75
34.35
27.52
25.03
20.96
18.72
18.34
17.97
14.57
f1 (ppm)

References

[1] S. Hiraoka, S. Harada, A. Nishida, *J. Org. Chem.* **2010**, *75*, 3871 – 3874.

[2] A. G. Dossetter, T. F. Jamison, E. N. Jacobsen, *Angew. Chem. Int. Ed*. **1999**, *38*, 2398-2400.

[3] J. F. Kerwin, S. Danishefsky, *J. Org. Chem.* **1982**, *47*, 1597 – 1598.

[4] I. S. Young, M. W. Haley, A. Tam, S. A. Tymonko, Z. Xu, R. L. Hanson, A. Goswami, *Org. Process Res. Dev*. **2015**, *19*, 1360 – 1368.

[5] H. Paulsen, H. Redlich, *Chem. Ber*. **1974**, *107*, 2992 – 3012.

[6] J. A. Hoskins, D. J. Brown, *J. Chem Soc., Perkin Trans. 1*, **1972**, 522 – 527.

[7] C.-X. Wang, R. Ding, S.-T. Jiang, J.-S. Tang, D. Hu, G.-D. Chen, F. Lin, K. Hong, X.-S. Yao, H. Gao, *J. Nat. Prod*. **2016**, *79*, 2446-2454.
